# Autophagy in major human diseases

**DOI:** 10.15252/embj.2021108863

**Published:** 2021-08-30

**Authors:** Daniel J Klionsky, Giulia Petroni, Ravi K Amaravadi, Eric H Baehrecke, Andrea Ballabio, Patricia Boya, José Manuel Bravo‐San Pedro, Ken Cadwell, Francesco Cecconi, Augustine M K Choi, Mary E Choi, Charleen T Chu, Patrice Codogno, Maria Isabel Colombo, Ana Maria Cuervo, Vojo Deretic, Ivan Dikic, Zvulun Elazar, Eeva‐Liisa Eskelinen, Gian Maria Fimia, David A Gewirtz, Douglas R Green, Malene Hansen, Marja Jäättelä, Terje Johansen, Gábor Juhász, Vassiliki Karantza, Claudine Kraft, Guido Kroemer, Nicholas T Ktistakis, Sharad Kumar, Carlos Lopez‐Otin, Kay F Macleod, Frank Madeo, Jennifer Martinez, Alicia Meléndez, Noboru Mizushima, Christian Münz, Josef M Penninger, Rushika M Perera, Mauro Piacentini, Fulvio Reggiori, David C Rubinsztein, Kevin M Ryan, Junichi Sadoshima, Laura Santambrogio, Luca Scorrano, Hans‐Uwe Simon, Anna Katharina Simon, Anne Simonsen, Alexandra Stolz, Nektarios Tavernarakis, Sharon A Tooze, Tamotsu Yoshimori, Junying Yuan, Zhenyu Yue, Qing Zhong, Lorenzo Galluzzi, Federico Pietrocola

**Affiliations:** ^1^ Life Sciences Institute University of Michigan Ann Arbor MI USA; ^2^ Department of Radiation Oncology Weill Cornell Medical College New York NY USA; ^3^ Department of Medicine University of Pennsylvania Philadelphia PA USA; ^4^ Abramson Cancer Center University of Pennsylvania Philadelphia PA USA; ^5^ Department of Molecular, Cell and Cancer Biology University of Massachusetts Medical School Worcester MA USA; ^6^ Telethon Institute of Genetics and Medicine Pozzuoli Italy; ^7^ Department of Translational Medical Sciences Section of Pediatrics Federico II University Naples Italy; ^8^ Department of Molecular and Human Genetics Baylor College of Medicine, and Jan and Dan Duncan Neurological Research Institute Texas Children Hospital Houston TX USA; ^9^ Margarita Salas Center for Biological Research Spanish National Research Council Madrid Spain; ^10^ Faculty of Medicine Department Section of Physiology Complutense University of Madrid Madrid Spain; ^11^ Center for Networked Biomedical Research in Neurodegenerative Diseases (CIBERNED) Madrid Spain; ^12^ Kimmel Center for Biology and Medicine at the Skirball Institute New York University Grossman School of Medicine New York NY USA; ^13^ Department of Microbiology New York University Grossman School of Medicine New York NY USA; ^14^ Division of Gastroenterology and Hepatology Department of Medicine New York University Langone Health New York NY USA; ^15^ Cell Stress and Survival Unit Center for Autophagy, Recycling and Disease (CARD) Danish Cancer Society Research Center Copenhagen Denmark; ^16^ Department of Pediatric Onco‐Hematology and Cell and Gene Therapy IRCCS Bambino Gesù Children's Hospital Rome Italy; ^17^ Department of Biology University of Rome ‘Tor Vergata’ Rome Italy; ^18^ Division of Pulmonary and Critical Care Medicine Joan and Sanford I. Weill Department of Medicine Weill Cornell Medicine New York NY USA; ^19^ New York‐Presbyterian Hospital Weill Cornell Medicine New York NY USA; ^20^ Division of Nephrology and Hypertension Joan and Sanford I. Weill Department of Medicine Weill Cornell Medicine New York NY USA; ^21^ Department of Pathology University of Pittsburgh School of Medicine Pittsburgh PA USA; ^22^ Institut Necker‐Enfants Malades INSERM U1151‐CNRS UMR 8253 Paris France; ^23^ Université de Paris Paris France; ^24^ Laboratorio de Mecanismos Moleculares Implicados en el Tráfico Vesicular y la Autofagia‐Instituto de Histología y Embriología (IHEM)‐Universidad Nacional de Cuyo CONICET‐ Facultad de Ciencias Médicas Mendoza Argentina; ^25^ Department of Developmental and Molecular Biology Albert Einstein College of Medicine Bronx NY USA; ^26^ Institute for Aging Studies Albert Einstein College of Medicine Bronx NY USA; ^27^ Autophagy Inflammation and Metabolism (AIM Center of Biomedical Research Excellence University of New Mexico Health Sciences Center Albuquerque NM USA; ^28^ Department of Molecular Genetics and Microbiology University of New Mexico Health Sciences Center Albuquerque NM USA; ^29^ Institute of Biochemistry II School of Medicine Goethe University Frankfurt, Frankfurt am Main Germany; ^30^ Buchmann Institute for Molecular Life Sciences Goethe University Frankfurt, Frankfurt am Main Germany; ^31^ Department of Biomolecular Sciences The Weizmann Institute of Science Rehovot Israel; ^32^ Institute of Biomedicine University of Turku Turku Finland; ^33^ Department of Molecular Medicine Sapienza University of Rome Rome Italy; ^34^ Department of Epidemiology Preclinical Research, and Advanced Diagnostics National Institute for Infectious Diseases ‘L. Spallanzani’ IRCCS Rome Italy; ^35^ Department of Pharmacology and Toxicology School of Medicine Virginia Commonwealth University Richmond VA USA; ^36^ Department of Immunology St. Jude Children's Research Hospital Memphis TN USA; ^37^ Sanford Burnham Prebys Medical Discovery Institute Program of Development Aging, and Regeneration La Jolla CA USA; ^38^ Cell Death and Metabolism Center for Autophagy, Recycling & Disease Danish Cancer Society Research Center Copenhagen Denmark; ^39^ Department of Cellular and Molecular Medicine Faculty of Health Sciences University of Copenhagen Copenhagen Denmark; ^40^ Department of Medical Biology Molecular Cancer Research Group University of Tromsø—The Arctic University of Norway Tromsø Norway; ^41^ Institute of Genetics Biological Research Center Szeged Hungary; ^42^ Department of Anatomy, Cell and Developmental Biology Eötvös Loránd University Budapest Hungary; ^43^ Merck & Co., Inc. Kenilworth NJ USA; ^44^ Institute of Biochemistry and Molecular Biology ZBMZ Faculty of Medicine University of Freiburg Freiburg Germany; ^45^ CIBSS ‐ Centre for Integrative Biological Signalling Studies University of Freiburg Freiburg Germany; ^46^ Centre de Recherche des Cordeliers Equipe Labellisée par la Ligue Contre le Cancer Université de Paris Sorbonne Université Inserm U1138 Institut Universitaire de France Paris France; ^47^ Metabolomics and Cell Biology Platforms Institut Gustave Roussy Villejuif France; ^48^ Pôle de Biologie Hôpital Européen Georges Pompidou AP‐HP Paris France; ^49^ Suzhou Institute for Systems Medicine Chinese Academy of Medical Sciences Suzhou China; ^50^ Karolinska Institute Department of Women's and Children's Health Karolinska University Hospital Stockholm Sweden; ^51^ Signalling Programme Babraham Institute Cambridge UK; ^52^ Centre for Cancer Biology University of South Australia Adelaide SA Australia; ^53^ Faculty of Health and Medical Sciences University of Adelaide Adelaide SA Australia; ^54^ Departamento de Bioquímica y Biología Molecular Facultad de Medicina Instituto Universitario de Oncología del Principado de Asturias (IUOPA) Universidad de Oviedo Oviedo Spain; ^55^ Centro de Investigación Biomédica en Red de Cáncer (CIBERONC) Madrid Spain; ^56^ The Ben May Department for Cancer Research The Gordon Center for Integrative Sciences W‐338 The University of Chicago Chicago IL USA; ^57^ The University of Chicago Chicago IL USA; ^58^ Institute of Molecular Biosciences NAWI Graz University of Graz Graz Austria; ^59^ BioTechMed‐Graz Graz Austria; ^60^ Field of Excellence BioHealth – University of Graz Graz Austria; ^61^ Immunity, Inflammation and Disease Laboratory National Institute of Environmental Health Sciences NIH Research Triangle Park NC USA; ^62^ Biology Department, Queens College City University of New York Flushing NY USA; ^63^ The Graduate Center Biology and Biochemistry PhD Programs of the City University of New York New York NY USA; ^64^ Department of Biochemistry and Molecular Biology Graduate School of Medicine The University of Tokyo Tokyo Japan; ^65^ Viral Immunobiology Institute of Experimental Immunology University of Zurich Zurich Switzerland; ^66^ Institute of Molecular Biotechnology of the Austrian Academy of Sciences (IMBA) Vienna BioCenter (VBC) Vienna Austria; ^67^ Department of Medical Genetics Life Sciences Institute University of British Columbia Vancouver BC Canada; ^68^ Department of Anatomy University of California, San Francisco San Francisco CA USA; ^69^ Department of Pathology University of California, San Francisco San Francisco CA USA; ^70^ Helen Diller Family Comprehensive Cancer Center University of California, San Francisco San Francisco CA USA; ^71^ Department of Biology University of Rome “Tor Vergata” Rome Italy; ^72^ Laboratory of Molecular Medicine Institute of Cytology Russian Academy of Science Saint Petersburg Russia; ^73^ Department of Biomedical Sciences of Cells & Systems Molecular Cell Biology Section University of Groningen University Medical Center Groningen Groningen The Netherlands; ^74^ Department of Medical Genetics Cambridge Institute for Medical Research University of Cambridge Cambridge UK; ^75^ UK Dementia Research Institute University of Cambridge Cambridge UK; ^76^ Cancer Research UK Beatson Institute Glasgow UK; ^77^ Institute of Cancer Sciences University of Glasgow Glasgow UK; ^78^ Department of Cell Biology and Molecular Medicine Cardiovascular Research Institute Rutgers New Jersey Medical School Newark NJ USA; ^79^ Sandra and Edward Meyer Cancer Center New York NY USA; ^80^ Caryl and Israel Englander Institute for Precision Medicine New York NY USA; ^81^ Istituto Veneto di Medicina Molecolare Padova Italy; ^82^ Department of Biology University of Padova Padova Italy; ^83^ Institute of Pharmacology University of Bern Bern Switzerland; ^84^ Department of Clinical Immunology and Allergology Sechenov University Moscow Russia; ^85^ Laboratory of Molecular Immunology Institute of Fundamental Medicine and Biology Kazan Federal University Kazan Russia; ^86^ The Kennedy Institute of Rheumatology NDORMS University of Oxford Oxford UK; ^87^ Department of Molecular Medicine Institute of Basic Medical Sciences University of Oslo Oslo Norway; ^88^ Centre for Cancer Cell Reprogramming Institute of Clinical Medicine University of Oslo Oslo Norway; ^89^ Department of Molecular Cell Biology Institute for Cancer Research Oslo University Hospital Montebello Oslo Norway; ^90^ Institute of Molecular Biology and Biotechnology Foundation for Research and Technology‐Hellas Heraklion, Crete Greece; ^91^ Department of Basic Sciences School of Medicine University of Crete Heraklion, Crete Greece; ^92^ Molecular Cell Biology of Autophagy The Francis Crick Institute London UK; ^93^ Department of Genetics Graduate School of Medicine Osaka University Suita Japan; ^94^ Department of Intracellular Membrane Dynamics Graduate School of Frontier Biosciences Osaka University Suita Japan; ^95^ Integrated Frontier Research for Medical Science Division Institute for Open and Transdisciplinary Research Initiatives (OTRI) Osaka University Suita Japan; ^96^ Interdisciplinary Research Center on Biology and Chemistry Shanghai Institute of Organic Chemistry Chinese Academy of Sciences Shanghai China; ^97^ Department of Cell Biology Harvard Medical School Boston MA USA; ^98^ Department of Neurology Friedman Brain Institute Icahn School of Medicine at Mount Sinai New York NY USA; ^99^ Key Laboratory of Cell Differentiation and Apoptosis of Chinese Ministry of Education Department of Pathophysiology Shanghai Jiao Tong University School of Medicine (SJTU‐SM) Shanghai China; ^100^ Department of Dermatology Yale School of Medicine New Haven CT USA; ^101^ Université de Paris Paris France; ^102^ Department of Biosciences and Nutrition Karolinska Institute Huddinge Sweden

**Keywords:** aging, cancer, inflammation, metabolic syndromes, neurodegeneration, Autophagy & Cell Death

## Abstract

Autophagy is a core molecular pathway for the preservation of cellular and organismal homeostasis. Pharmacological and genetic interventions impairing autophagy responses promote or aggravate disease in a plethora of experimental models. Consistently, mutations in autophagy‐related processes cause severe human pathologies. Here, we review and discuss preclinical data linking autophagy dysfunction to the pathogenesis of major human disorders including cancer as well as cardiovascular, neurodegenerative, metabolic, pulmonary, renal, infectious, musculoskeletal, and ocular disorders.

## Graphical abstract

This review provides an exhaustive overview of the contribution of autophagy to multiple pathological phenotypes *in vivo*, and discusses the therapeutic potential of autophagy modulation in disease prevention and treatment.

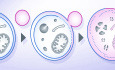

**Glossary**
AD: Alzheimer disease
ALS: amyotrophic lateral sclerosis
ARMD: age‐related macular degeneration
ATG: autophagy related
ATZ: mutant Z variant of SERPINA1/alpha‐1 antitrypsin
CF: cystic fibrosis
CMA: chaperone‐mediated autophagy
CNS: central nervous system
COPD: chronic obstructive pulmonary disease
CS: cigarette smoke
CTLs: cytotoxic T lymphocyte
DC: dendritic cell
DKD: diabetic kidney disease
FA: free fatty acid
FTD: frontotemporal dementia
GEMM: genetically engineered mouse model
HD: Huntington disease
HFD: high‐fat diet
IBD: inflammatory bowel disease
IFN: interferon
IOP: intraocular pressure
IRI: ischemia‐reperfusion injury
LANDO: LC3‐associated endocytosis
LAP: LC3‐associated phagocytosis
LDs: lipid droplets
LECs: lens epithelial cells
mtDNA: mitochondrial DNA
NAFLD: non‐alcoholic fatty liver disease
NK: natural killer
NTG: normal tension glaucoma
OA: osteoarthritis
PD: Parkinson disease
PDAC: pancreatic ductal carcinoma
PDB: Paget disease of bone
polyQ: polyglutamine
PtdIns3K: class III phosphatidylinositol‐3‐kinase
RGC: retinal ganglion cell
ROS: reactive oxygen species
RPE: retinal pigment epithelium
T2D: type 2 diabetes
TECs: epithelial tubular cells
TME: tumor microenvironment
T_REG_
: regulatory T cells
UUO: unilateral ureteral obstruction
WAT: white adipose tissue

## Introduction

The staggering increase in life expectancy that has characterized the last century has progressively attenuated, until reaching an apparent plateau over the last decade. Conversely, aging increases the susceptibility to many chronic illnesses, a condition that poses a major threat to the socioeconomic stability of high‐ and low‐income countries (Kehler, 2019; Melzer *et al*, 2020). Consequently, the trajectories of human lifespan and healthspan are estimated to diverge in the near future. During the last decade, investigators have endeavored to put forward a holistic view of the biological principles underlying the general concepts of “health” and “disease” at the cellular and organismal levels, by framing them into archetypical “hallmarks” (Lopez‐Otin *et al*, 2013; Kennedy *et al*, 2014; Lopez‐Otin & Kroemer, 2021). On these bases, it has been possible to separate the quintessential processes that operate to maintain individual cells and multicellular entities in a “healthy” state, from those that perturb the *status quo* of cells and tissues, thereby hastening the clinical onset of life‐threatening diseases.

In this context, the process of autophagy can be considered as a *bona fide* health‐modifying agent (Choi *et al*, 2013; Mizushima & Levine, 2020). Indeed, a large body of evidence from the literature supports the view of autophagy as a pro‐longevity mechanism (Morselli *et al*, 2009; Morselli *et al*, 2010; Rubinsztein *et al*, 2011; Kaushik & Cuervo, 2015b; Madeo *et al*, 2015; Fernandez *et al*, 2018; Hansen *et al*, 2018; Leidal *et al*, 2018; Markaki *et al*, 2018) and as a cardinal regulator of cellular and organismal fitness in response to multiple endogenous or exogenous sources of stress (Mizushima, 2018; Morishita & Mizushima, 2019). Conversely, time‐dependent loss of autophagy proficiency is thought to critically contribute to the aged phenotype (Lopez‐Otin *et al*, 2013; Kennedy *et al*, 2014; Lopez‐Otin & Kroemer, 2021). Furthermore, several of the lifestyle changes that have been attributed a positive role in the regulation of longevity (including calorie restriction and physical exercise) are commonly noted for their capacity to stimulate autophagy (Lopez‐Otin *et al*, 2016).

Autophagy is also key in preventing stresses as one of the major quality control guardians in the cell (Mancias & Kimmelman, 2016; Conway *et al*, 2020). Noteworthy, the autophagy pathways acquire physiological relevance even under basal, non‐stressful conditions. In line with this notion, autophagy takes direct part in the regulation of developmental programs (Mizushima & Levine, 2010; Allen & Baehrecke, 2020), maintenance of stem cell self‐renewal potential (Chen *et al*, 2018c; Dong *et al*, 2021a), cellular differentiation and plasticity (Boya *et al*, 2018; Clarke & Simon, 2019). Concordant with this notion, the appearance of the “diseased” state associated with autophagy dysregulation may occur as a result of alterations in these central aspects of multicellular organism biology. Indeed, tissues that are mainly composed of cells that lay in a post‐mitotic/quiescent state exhibit higher sensitivity to loss of autophagy competence.

The term “autophagy” refers to composite molecular pathways in which intracellular components are conveyed to the lysosomal compartment for degradation and recycling. To date, three major forms of autophagy have been described (Galluzzi *et al*, 2017a). Macroautophagy (henceforth referred to as autophagy; Box 1) is a form of autophagy in which the cellular cargo becomes sequestered within a double‐membraned vesicle, termed an autophagosome. The choice of the autophagosomal content can proceed in a relatively nonselective manner (known as “bulk autophagy”) or involve the tightly regulated elimination of individual cellular components (known as “selective autophagy”), depending on the inducing factor (He & Klionsky, 2009; Sica *et al*, 2015; Dikic & Elazar, 2018; Gohel *et al*, 2020). By contrast, chaperone‐mediated autophagy (CMA) operates as a protein‐exclusive type of autophagy in which KFERQ‐like motif‐bearing proteins are first recognized by the heat‐shock cognate protein HSPA8/HSC70 and enter the lysosome for degradation, upon binding LAMP2A (lysosomal‐associated membrane protein 2A) and translocation through a channel formed by oligomerization of this protein (Kaushik & Cuervo, 2018). Finally, microautophagy involves the sequestration of cellular material (including KFERQ‐flagged proteins or bulk cytoplasmic content) directly via membranous invaginations formed at the surface of late endosomes or lysosomes (Sahu *et al*, 2011; Uytterhoeven *et al*, 2015; Mejlvang *et al*, 2018), in an ESCRT‐dependent (Sahu *et al*, 2011) or ESCRT‐independent (McNally & Brett, 2018) mode. Besides representing the terminal effector of the autophagy cascade, the lysosome operates as a primary regulator of the autophagy process, in light of its active role in nutrient sensing and signaling via the MTOR (mechanistic target of rapamycin kinase) complex 1 (MTORC1)‐TFEB (transcription factor EB) axis (Ballabio & Bonifacino, 2020).

**Box 1. Core regulation of canonical autophagy**

Canonical autophagy is a multiphasic process that involves the sequential and selective recruitment of ATG (autophagy related) proteins (Galluzzi *et al*, 2017a). The initiation of the autophagic cascade is physiologically subjected to the repressive control of MTOR (mechanistic target of rapamycin kinase) complex 1 (MTORC1), which catalyzes the inactivating phosphorylation of ATG13 and ULK1 (unc‐51‐like autophagy‐activating kinase 1). ULK1 and ATG13 are found in a supramolecular complex that also contains RB1CC1 (RB1‐inducible coiled‐coil 1) and ATG101, which cooperates with ATG9 to promote autophagosome nucleation. The inhibitory action of MTORC1 is counterbalanced by AMP‐activated protein kinase (AMPK), which responds to dwindling ATP levels by phosphorylating ULK1 and BECN1 (Beclin 1). ULK1 favors the autophagic cascade by facilitating the phosphatidylinositol‐3‐kinase activity of a multiprotein complex formed by BECN1, PIK3C3/VPS34 (phosphatidylinositol‐3‐kinase catalytic subunit type 3), PIK3R4/VPS15 (phosphoinositide‐3‐kinase regulatory subunit 4), ATG14, and NRBF2 (nuclear receptor binding factor 2). Multiple regulatory interactors of the BECN1‐PIK3C3/VPS34 complex have been identified, including UVRAG (UV radiation resistance associated), SH3GLB1 (SH3 domain containing GRB2 like, endophilin B1), and AMBRA1 (autophagy and Beclin 1 regulator 1), which facilitate the catalytic activity of PIK3C3/VPS34, as well as RUBCN (rubicon autophagy regulator) and BCL2 (BCL2 apoptosis regulator), which inhibit it. The production of phosphatidylinositol‐3‐phosphate (PtdIns3P), followed by the engagement of PtdIns3P‐binding proteins of the WIPI (WD repeat domain, phosphoinositide interacting) family, is instrumental for the expansion of phagophores. This phase is promoted by two distinct ubiquitin‐like conjugation modules. The first relies upon the activity of ATG7 and ATG10 and enables the buildup of a multiprotein complex composed of ATG5, ATG12 and ATG16L1 (autophagy‐related 16‐like 1). The second one involves ATG3, ATG4, and ATG7 and is ultimately responsible for the cleavage of members of the Atg8‐family proteins, including mammalian MAP1LC3/LC3 (microtubule‐associated protein 1 light chain 3) and their conjugation to phosphatidylethanolamine (PE). Lipidated LC3 (LC3‐II; which is experimentally employed for quantifying autophagy *in vitro* and *in vivo*) serves as a receptor for LC3‐interacting region (LIR)‐containing proteins, including autophagy substrates and receptors such as SQSTM1/p62 (sequestosome 1). Upon closure of the phagophore, the resulting autophagosome fuses with a lysosome to form an autolysosome, culminating with the degradation of autophagic substrates by acidic lysosomal hydrolases. AKT1S1, AKT1 substrate 1; DEPTOR, DEP domain containing MTOR interacting protein; MLST8, MTOR‐associated protein, LST8 homolog; RPTOR, regulatory‐associated protein of MTOR complex 1.

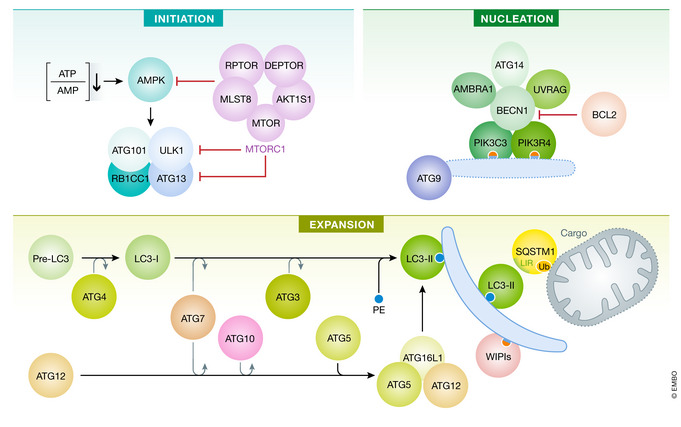

The complex molecular networks that underlie these distinct autophagic pathways, as well as other forms of canonical and non‐canonical autophagy that will be mentioned in this review, have been the object of thorough investigation and extensive reviewing over recent years (Dupont *et al*, 2017; Galluzzi *et al*, 2017a; Dikic & Elazar, 2018; Kaushik & Cuervo, 2018; Chu, 2019; Kirkin & Rogov, 2019; Nakatogawa, 2020; Klionsky *et al*, 2021). Whereas autophagy proceeds at a basal (yet cell type dependent) rate in virtually all eukaryotic cells—inherent to its housekeeping function in the turnover of superfluous or damaged organelles and long‐lived proteins—a prominent surge in the magnitude of the autophagic reaction occurs upon disturbance of the intracellular or environmental homeostasis (He & Klionsky, 2009; Mizushima & Komatsu, 2011). From an evolutionary perspective, autophagy primarily equips cells with the ability to maintain viability under nutrient‐restricted conditions, conferring autophagy‐competent cells a survival advantage over their autophagy‐defective counterparts (Galluzzi *et al*, 2014; Lahiri *et al*, 2019; Morishita & Mizushima, 2019). This notion is fully supported by the finding that whole‐body autophagy‐deficient mice undergo perinatal death due to their inability to withstand postnatal starvation (Kuma *et al*, 2004; Komatsu *et al*, 2005; Kuma *et al*, 2017). Moreover, insightful evidence generated from preclinical models of partial or tissue‐specific autophagy deficiency has contributed to broaden the physiological relevance of this pathway to several aspects of multicellular organism biology (Kuma *et al*, 2017; Levine & Kroemer, 2019). As selection pressure shifts from individual cell survival to reproductive fitness, however, autophagy regulation grows in complexity and the outcome of autophagy upregulation is less predictable (Cherra & Chu, 2008). For example, autophagy can engage in cell death (Fairlie *et al*, 2020; Miller *et al*, 2020), directly contributing to the pathogenesis of some human diseases (e.g., ischemia‐reperfusion injury, neuronal, and muscle atrophy) (Galluzzi *et al*, 2018b; Galluzzi *et al*, 2018c; Patel & Karch, 2020; Pervaiz *et al*, 2020).

The autophagy machinery participates in intercellular communication, mediating processes of non‐canonical protein secretion (an autophagy‐independent function of autophagy proteins) (Ponpuak *et al*, 2015; Zahoor & Farhan, 2018), regulation of tissue‐resident stem cells (Guan *et al*, 2013; Chang, 2020), modulation of immune cell functions (Deretic, 2021), and maintenance of tissue barrier integrity (Galluzzi & Green, 2019; Levine & Kroemer, 2019). As an example, in dendritic cells (DCs) autophagy and microautophagy serve the important role of feeding endogenous proteins to endosomal/lysosomal compartments for MHC class II molecule‐mediated immunosurveillance (Balan *et al*, 2019; Kotsias *et al*, 2019), and the biogenesis of endosomal microautophagy is tightly connected to exosomal production (Sahu *et al*, 2011). As yet another example, in phagocytic cells several components of the autophagy machinery (including the phosphatidylinositol‐3‐kinase [PtdIns3K] complex, but not ULK1 [unc‐51‐like autophagy‐activating kinase 1]) are recruited to the single‐layered phagosomal membrane, following the engagement of cell surface receptors (e.g., TLRs [Toll‐like receptors]) by pathogen‐associated molecules (Martinez *et al*, 2015), immune complexes (Henault *et al*, 2012), or phosphatidylserine exposed by apoptotic cells (Martinez *et al*, 2011). This process, defined as LC3‐associated phagocytosis (LAP) (Heckmann & Green, 2019), exquisitely relies upon CYBB/NOX2 (cytochrome b‐245, beta polypeptide), RUBCN (rubicon autophagy regulator), and the WD domain of ATG16L1 (autophagy‐related 16‐like 1), which are dispensable for the execution of canonical autophagy (Martinez *et al*, 2015).

**Figure 1:**
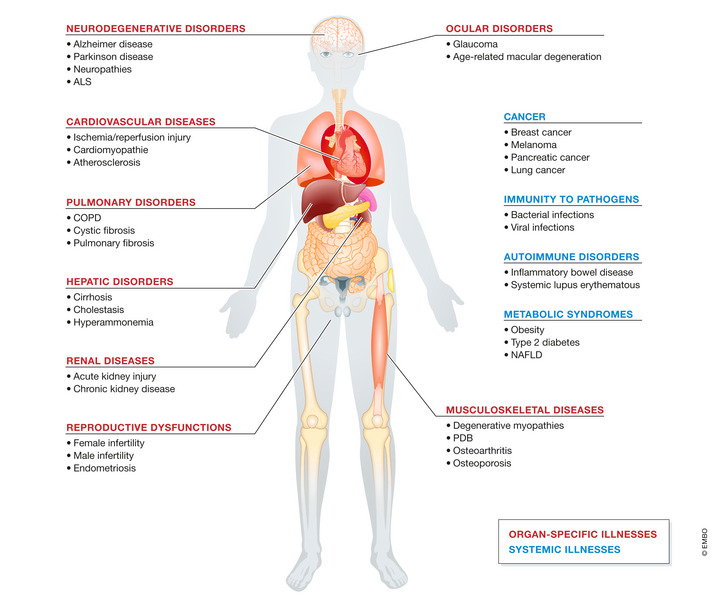
Common human disorders linked to dysregulated autophagic activity Representation of the main organ‐specific (red) and systemic (blue) human illnesses in which autophagy plays a critical role and that are discussed in this review. ALS, amyotrophic lateral sclerosis; COPD, chronic obstructive pulmonary disease; DKD, diabetic kidney disease; NAFLD, non‐alcoholic fatty liver disease; PDB, Paget disease of bone.

The multitiered repercussions of autophagy on organismal homeostasis have spurred considerable efforts toward the identification of clinically actionable targets to modulate the autophagic pathway to prevent or treat diseases, in multiple pathological circumstances (Galluzzi *et al*, 2017c). Our current understanding about the contribution of autophagy in human disorders mostly derives from (i) the implementation of several mouse models of autophagy deficiency (Kuma *et al*, 2004), through which the role of autophagy can be interrogated at the whole body, or in a cell type‐specific manner, and (ii) from the discovery that several components of the autophagic machinery have been found mutated in human diseases (van Beek *et al*, 2018; Levine & Kroemer, 2019). Here, we discuss recent insights on the role of autophagy in the most penetrant human illnesses (Fig 1), placing particular emphasis on preclinical findings obtained in murine models of diseases in which autophagy has been genetically dismantled. In this regard, the involvement of virtually all ATG (autophagy related) proteins in autophagy‐independent tasks imposes a note of caution on the attribution of specific phenotypic effects to the mere inhibition of the autophagy process (Galluzzi & Green, 2019).

## Neurodegenerative disorders

The autophagic process is essential in preserving the homeostatic requirements of post‐mitotic neurons, both at the central and at the peripheral nervous system levels (Menzies *et al*, 2017; Scrivo *et al*, 2018; Mallucci *et al*, 2020) (Table 1). Most neurodegenerative diseases are associated with the accumulation of aggregate‐prone proteins. Studies performed in diseases with Mendelian‐type inheritance suggest that these proteins are toxic drivers that are necessary and sufficient to cause pathology. A large body of evidence, supported by the demonstration that *ATG* genes are found mutated in multiple human neurodegenerative illnesses, indicates that autophagy directly intervenes in the clearance of those proteins (Nixon, 2013). In addition, MTOR p.Cys1483Tyr somatic mutation resulted in impaired autophagy, caused aberrant accumulation of OFD1, and disrupted neuronal ciliogenesis, which accounted for cortical dyslamination in Focal malformations of cortical development (Tang *et al*, 2013; Park *et al*, 2018). Furthermore, intact autophagy responses have been postulated to extinguish neuroinflammatory reactions, which directly contribute to the aetiopathogenesis of neurodegenerative disorders (Rubinsztein *et al*, 2015). For these reasons, upregulation of autophagy has attracted particular interest as a potential therapeutic strategy for various neurodegenerative conditions (Menzies *et al*, 2017; Thangaraj *et al*, 2020).

**Table 1:** Neurodegenerative disorders associated with genetic intervention of autophagy in mice.

Setting	Genetic intervention	Effects on disease phenotype	Ref.
Alzheimer disease	Myeloid cell‐specific deletion of *Trim16*	Exacerbated endomembrane damage post‐infection with *Mycobacterium tuberculosis*	Jia *et al* (2020)
Alzheimer disease	Whole‐body deletion of *Sqstm1*/*p62*	Accumulation of hyperphosphorylated MAPT/tau and neurodegeneration	Ramesh Babu *et al* (2008)
Alzheimer disease	Whole‐body deletion of *Nrf2*	Aberrant accumulation of phosphorylated and sarkosyl‐insoluble tau protein	Jo *et al* (2014)
Alzheimer disease	Conditional excitatory neuron‐specific deletion of *Atg7*	Reduced extracellular Aβ plaque burden, linked to cognitive dysfunction in APP transgenic mice	Nilsson *et al* (2013)
Alzheimer disease	Whole‐body deletion of *Nrbf2*	Impaired cognitive fitness and increased Aβ plaque accumulation	Lachance *et al* (2019)
Alzheimer disease	Whole‐body deletion of *Trem2*	Impaired metabolic fitness and increased accumulation of autophagic vesicles in the microglia of 5XFAD mice	Ulland *et al* (2017)
Alzheimer disease	Conditional myeloid cell‐specific deletion of *Atg5* or *Rubcn*	Exacerbated Aβ plaque accumulation and inflammation within the hippocampus of young 5xFAD mice	Heckmann *et al* (2019)
Alzheimer disease	Whole‐body deletion of *Atg16L^ΔWD^*	Exacerbated Aβ plaque accumulation, neuroinflammation and Tau hyperphosphorylation	Heckmann *et al* (2020)
Alzheimer disease	Neuron‐specific deletion of *Lamp2*	Exacerbated Tau acetylation, extraneuronal release and propagation, linked to accelerated disease progression	Bourdenx *et al* (2021), Caballero *et al* (2021)
Amyotrophic lateral sclerosis	Whole‐body deletion of *Epg5*	Muscle denervation, myofiber atrophy, late‐onset progressive paralysis, and reduced survival	Zhao *et al* (2013)
Amyotrophic lateral sclerosis	Conditional motoneuron‐specific deletion of *Tbk1*	Accelerated early disease onset in SOD1^G93A^ mice, linked to increased accumulation of ubiquitinated aggregates	Gerbino *et al* (2020)
Amyotrophic lateral sclerosis	Whole‐body knock‐in of mutant *Tbk1^G217R^* or *Tbk1^R228H^*	Accelerated early disease onset but extended lifespan in SOD1^G93A^ mice, linked to reduced microglia IFN response	Gerbino *et al* (2020)
Amyotrophic lateral sclerosis	Whole‐body deletion of *Grn*	Exacerbated symptomatology linked to increased accumulation of pathological TDP‐43 in neurons	Chang *et al* (2017)
Amyotrophic lateral sclerosis	Conditional neuron‐specific deletion of *Xbp1*	Reduced disease onset in SOD1^G93A^ mice after inducing autophagy in motoneurons	Hetz *et al* (2009)
Amyotrophic lateral sclerosis	AAV‐mediated hippocampal‐specific deletion of *C9orf72*	Exacerbated cognitive and motor deficits, hippocampal neuron loss, and DPR protein accumulation, after autophagy inhibition	Zhu *et al* (2020)
Amyotrophic lateral sclerosis	Whole‐body allelic loss of *Becn1*	Increased lifespan of mutant SOD1 transgenic mice	Nassif *et al* (2014)
Focal malformations of cortical development	Brain somatic mutations in MTOR	Cortical abnormalities that are highly associated with medically intractable epilepsy, intellectual disability, developmental delay, and autism‐spectrum disorders	Park *et al* (2018)
Axon growth	POMC neuron‐specific deletion of *Atg7*	Abnormal development of POMC neuronal projections, associated with metabolic dysregulations	Coupe *et al* (2012)
Cognitive fitness	shRNA‐dependent hippocampal‐specific deletion of *Becn1, Atg12* or *Rb1cc1*	Impaired capacity to generate novel memories	Glatigny *et al* (2019)
Food intake and energy balance	AgRP neuron‐specific deletion of *Atg7*	Increased neuronal lipid accumulation, associated with altered energy balance and food intake after starvation	Kaushik *et al* (2011)
Huntington disease	Conditional whole‐body deletion of *WDFY3*/*ALFY*	Accumulation of proteinaceous deposits, linked to accelerated onset and progression of Huntington disease pathogenesis	Fox *et al* (2020)
Ischemic brain damage	Whole‐body allelic loss of *Sod2*	Increased infarct volume under hyperglycemic conditions, linked to increased oxidative DNA damage	Mehta *et al* (2011)
Ischemic brain damage	Neuron‐specific deletion of *Atg7*	Complete protection from neonatal hypoxic/ischemic brain injury	Koike *et al* (2008), Xie *et al* (2016)
Nerve injury	Schwann cell‐specific deletion of *Atg7*	Delayed myelin degradation and generation of repair cells after injury	Gomez‐Sanchez *et al* (2015)
Neurodegeneration	Neural cell‐specific deletion of *Atg5*	Development of progressive deficits in motor function linked to cytoplasmic inclusion body accumulation in neurons	Hara *et al* (2006)
Neurodegeneration	Conditional CNS‐specific deletion of *Atg7*	Behavioral defects and premature death, linked to massive neuronal loss and cytoplasmic inclusion body accumulation	Komatsu *et al* (2006)
Neurodegeneration	Conditional radial glial cell‐specific deletion of *Rb1cc1*	Progressive loss of NSCs pool and impaired neuronal differentiation in the postnatal brain	Wang *et al* (2013)
Neurodegeneration	Conditional CNS‐specific deletion of *Wdr45*	Reduced motor coordination, impaired learning and memory, and extensive axon swelling	Zhao *et al* (2015)
Neurodegeneration	Conditional neuron‐specific deletion of *Wipi3*	Behavioral defects and cerebellar neuronal loss after non‐canonical autophagy inhibition	Yamaguchi *et al* (2020)
Neurodegeneration	Conditional telencephalon‐specific deletion of *Vps15*	Severe progressive cortical atrophy associated with caspase‐induced apoptosis	Gstrein *et al* (2018)
Neurodegeneration	Whole‐body knock‐in of hypomorphic *Atg16l1*	Developmental retention due to delayed differentiation of stem cells in the brain	Wu *et al* (2016)
Neurodegeneration	Conditional NSC‐specific co‐deletion of *FoxO1, FoxO3 and FoxO4*	Initial proliferation of neural progenitor cells in early postnatal life, followed by NSC pool decline in adult brains	Paik *et al* (2009)
Neurodegeneration	Purkinje cell‐specific deletion of *Atg7*	Progressive cell autonomous dystrophy and degeneration of the axon terminals	Komatsu *et al* (2007)
Neurodegeneration	Whole‐body deletion of *TAX1BP1*	Aberrant accumulation of high molecular weight ubiquitin conjugates and lipofuscin	Sarraf *et al* (2020)
Neuropathies	Whole‐body deletion of *Fam134b*	Degeneration of sensory neurons after inhibition of ER‐phagy	Khaminets *et al* (2015)
Neuropathies	Whole‐body deletion of *Tecpr2*	Exacerbated age‐dependent behavioral aberrations and neuroaxonal dystrophy, after accumulation of autophagosomes	Tamim‐Yecheskel *et al* (2020)
Neurotransmission	Post‐mitotic excitatory neuron‐specific deletion of *Atg5*	Increased accumulation of tubular ER in axons, linked to increased excitatory neurotransmission and premature death	Kuijpers *et al* (2021)
Parkinson disease	Microglia‐specific deletion of *Atg7*	Increased α‐synuclein accumulation and neurodegeneration	Choi *et al* (2020)
Parkinson disease	Whole‐body deletion of *Rubcn*	Reduced α‐synuclein accumulation in the brain, linked to reduced age‐related interstitial fibrosis in kidney	Nakamura *et al* (2019)
Parkinson disease	Conditional SN neuron‐specific deletion of *Atg7*	Resistance to retrograde axonal degeneration	Cheng *et al* (2011)
Parkinson disease	AAV‐mediated SN‐specific knock‐in of dominant‐negative Ulk1	Attenuated MPTP‐induced axonal neurodegeneration	Balke *et al* (2020)
Parkinson disease	Whole‐body deletion of *Prkn*	Impaired striatal neural plasticity, linked to increased sensitivity to oxidative damage and mitochondrial dysfunction (exacerbated in Mutator mice but rescued by loss of STING)	Goldberg *et al* (2003), Palacino *et al* (2004), Kitada *et al* (2009), Pickrell *et al* (2015), Sliter *et al* (2018)
Parkinson disease	Whole‐body deletion of *Pink1*	Increased sensitivity to oxidative damage and mitochondrial dysfunction	Gautier *et al* (2008)

AAV, adeno‐associated viral vector; AgRP, agouti‐related protein; APP, amyloid precursor protein; CNS, central nervous system; DPR, dipeptide‐repeated; MPTP, 1‐methyl‐4‐phenyl‐1,2,3,6‐tetrahydropyridine; NSCS, neural stem cell; OGD, oxygen glucose deprivation; POMC, proopiomelanocortin; SN, substantia nigra; TDP‐43, transactive response DNA‐binding protein of 43 kD.

The neuroprotective functions attributed to autophagy are estimated to transcend its well‐defined roles as proteostasis keeper and organelle turnover regulator. Indeed, several findings have underscored that the ATG machinery is functionally implicated in compartment‐specific tasks along the soma‐axon axis that include, among others, (i) the regulation of synaptic transmission (Kuijpers *et al*, 2021), (ii) the degradation of synaptic cargoes and vesicles, (iii) the anterograde/retrograde crosstalk between cell body and synaptic terminal, and (iv) myelination/demyelination events (Hill & Colon‐Ramos, 2020). With these compartment‐specific physiological functions, it is no surprise that both insufficient and overactive nonselective or selective autophagy responses contribute to neurodegeneration (Chu, 2019).

Due to perinatal lethality related to ubiquitous inhibition of autophagy, our current degree of knowledge regarding the relevance of autophagy within the neural lineage mostly stems from fruit flies (Juhasz *et al*, 2007; Simonsen *et al*, 2008) and mouse models in which essential (i.e., *Atg5, Atg7, Rb1cc1*/*Fip200* [RB1‐inducible coiled‐coil 1]) (Hara *et al*, 2006; Komatsu *et al*, 2006; Wang *et al*, 2013) or non‐essential (i.e., *Wdr45*/*Wipi4* [WD repeat domain 45], and *Wdr45b*/*Wipi3*) (Zhao *et al*, 2015; Ji *et al*, 2020; Yamaguchi *et al*, 2020) autophagic genes have been obliterated at the embryonic stage by virtue of *Nes* (nestin)‐driven Cre recombinase expression. Compared to their wild‐type littermates, mice that developmentally lack autophagy in the neuronal compartment display shortened lifespan and early‐onset neurodegenerative pathologies (whose severity varies depending on the targeted gene), associated with the pathological accumulation of proteinaceous aggregates in multiple neuronal populations (Hara *et al*, 2006; Komatsu *et al*, 2006; Metcalf *et al*, 2012). Neuronal dysfunctions account for the lethality associated with systemic autophagic deficiency, as testified to by the fact that overexpression of *Atg5* in the neuronal compartment rescues perinatal mortality of *atg5^−^* ^/^^−^ mice (Yoshii *et al*, 2016). Blunted expression of *PIK3R4*/*VPS15* (phosphoinositide‐3‐kinase regulatory subunit 4) is associated with neurodevelopmental impairment and cortical atrophy, matching the phenotype of patients bearing loss‐of‐function mutations in this gene (Gstrein *et al*, 2018). Along similar lines, *de novo* mutations in the autophagy gene *WDR45* have been found in causal association with static encephalopathy of childhood with neurodegeneration in adulthood (also known as neurodegenerative disease β‐propeller protein‐associated neurodegeneration [BPAN]), a subtype of neurodegeneration with brain iron accumulation (NBIA) (Saitsu *et al*, 2013) and with human neurodegeneration (Suleiman *et al*, 2018). Supporting the possible involvement of autophagy in this pathology, abnormal early autophagosomal structures have been identified in patient‐derived lymphoblastoid cell lines (LCLs) (Saitsu *et al*, 2013). In concordance with this result, CNS‐specific *wdr45* knockout mice are defined by BPAN‐like features, including cognitive defects and impaired axonal homeostasis, but not other ones like iron accumulation in basal ganglia (Zhao *et al*, 2015). More recently, a mutation in *Wipi2* (WD‐repeat protein interacting with phosphoinositide 2) has been identified, linking defective autophagy to the appearance of complex neurodevelopmental defects (Jelani *et al*, 2019). Impaired autophagosome–lysosome fusion, associated with loss‐of‐function mutations in *EPG5* (ectopic P‐granule autophagy protein 5 homolog), causes autosomal recessive Vici syndrome (VICIS), pathologically defined by severe neurodevelopmental defects (Hori *et al*, 2017). The suppression of ATG5 expression during early brain development alters the differentiation trajectories and the rate of proliferation of neuronal progenitor cells, which eventually reflect into morphological defects in differentiated neurons. By analogy, a comparable phenotype has been described in *Atg16l1* hypomorphic mice (Lv *et al*, 2014; Wu *et al*, 2016; Menzies *et al*, 2017). Recently, a missense mutation in *ATG5* has been found in causal association with the manifestation of ataxia, with neurodevelopmental delay in human patients. Notably, the introduction of human mutated *ATG5* in flies is sufficient to recapitulate the clinical feature of the human disorders (Kim *et al*, 2016).

Disturbance in the autophagic process also has an impact on neurogenesis, which testifies to the central role of autophagy in the maintenance of adult neural stem cell pools within the sub‐ventricular zone (SVZ) of the lateral ventricle wall and subgranular zone (SGZ) of the dentate gyrus (Fleming & Rubinsztein, 2020). Consistent with this finding, inhibition of autophagy elicited by *Rb1cc1* ablation reduces differentiation potential and number of adult neural stem cells (Wang *et al*, 2013). Likewise, combined conditional deletion of genes coding for FOXO (forkhead box, sub‐group O; *Foxo1, Foxo3,* and *Foxo4*) in adult neural stem/progenitor cells correlates with abnormal morphological features of differentiated neurons (Paik *et al*, 2009).

Throughout the last decade, several mouse models of conditional autophagy disruption in specific populations of the CNS and peripheral nervous system have been implemented, revealing the cell type‐specific contribution of autophagy. These encompass Purkinje cells in the cerebellum (leading to progressive dystrophy) (Komatsu *et al*, 2007), hypothalamic AGRP (agouti‐related neuropeptide) neurons (evoking altered energy balance and food intake after starvation) (Kaushik *et al*, 2011), POMC (proopiomelanocortin) neurons (perturbing axon growth and decreasing α‐melanocyte‐stimulating hormone [MSH] levels) (Coupe *et al*, 2012; Kaushik *et al*, 2012), and Schwann cells (delaying the process of demyelination after injury) (Gomez‐Sanchez *et al*, 2015).

Functional autophagic responses are instrumental for preserving neuronal integrity upon circumstances of acute injury (Galluzzi *et al*, 2016). For example, it has been shown that a central role of autophagy is restraining the life‐threatening effect tied to brain ischemic challenge. In mice in which cerebral stroke was induced by transient middle carotid occlusion (MCAO), genetic interventions that undermine autophagy, including *Sod2* (superoxide dismutase 2, mitochondrial) inactivation (Mehta *et al*, 2011) or shRNA‐mediated silencing of *Tsc1* (TSC complex subunit 1) (Papadakis *et al*, 2013), aggravate the neurological sequelae instigated by the stroke episode. In apparent contrast with this finding, pharmacological inhibition of autophagy with 3‐methyladenine or bafilomycin A_1_ was observed to limit infarct size in a permanent MCAO, suggesting that autophagy may rather aggravate the ischemic injury (Zhang *et al*, 2013; Galluzzi *et al*, 2016). Although the reduced specificity of these pharmacological modulators limits the mechanistic interpretation of these results, it is nonetheless reasonable to propose that the actual contribution of autophagy in stroke‐associated neurotoxicity would vary depending upon the cerebral compartment affected and the developmental stage in which the ischemic episode occurs (Galluzzi *et al*, 2016). In support of this concept, brain‐specific deletion of *Atg7* confers protection against neonatal hypoxia–ischemia injury in mice (Koike *et al*, 2008; Xie *et al*, 2016).

Intact hippocampal autophagy sustains the elevated degree of synaptic plasticity required to generate novel memories, as demonstrated by the fact that stereotactic delivery of shRNA targeting key autophagy genes (including *Becn1* [Beclin 1, autophagy related], *Rb1cc1*, and *Atg12*) impairs cognitive fitness in mice (Glatigny *et al*, 2019). This effect, which can be phenocopied by pharmacological inhibition of autophagy (e.g., with spautin‐1, leupeptin, or chloroquine) and reversed by pharmacological activation of the ATG machinery with a Tat‐Beclin 1 peptide, supports the essential role of autophagy in dendritic spine formation and long‐term potentiation after stimuli (Glatigny *et al*, 2019). Of note, loss of autophagy performance may causally underlie the age‐dependent decline in memory tasks, as demonstrated by the fact that treatment of old mice with plasma derived from young donors improves cognitive fitness and restores normal levels of autophagy in the hippocampus (Glatigny *et al*, 2019). Further corroborating this result, dietary supplementation with spermidine, which also acts as an autophagy stimulator, mitigates age‐dependent cognitive impairment in mouse hippocampus and *Drosophila* heads, contingent upon intact autophagy and mitophagy responses (Schroeder *et al*, 2021).

In the recent past, autophagy has gained attention for its potential involvement in the pathogenesis of late‐onset neurodegenerative pathologies, owing to the historically rooted view of this pathway as a major determinant of long‐lived/aggregation‐prone protein disposal within the lysosome (Nixon, 2013; Menzies *et al*, 2017). Supporting this view, it has been demonstrated that the lack of the autophagic receptor TAX1BP1 (Tax1‐binding protein 1) results in aberrant protein aggregation in the brain (Sarraf *et al*, 2020). Although these disorders mainly follow a multifactorial pattern, evidence obtained from inherited variants of neurodegenerative illnesses has shed new light on the contribution of autophagy to the progressive loss of neural function.

### Alzheimer disease

Alzheimer disease (AD) represents the most common form of dementia in humans, caused by the pathologically relevant accumulation of proteinaceous aggregates, i.e., intracellular MAPT/tau tangles and/or extracellular beta amyloid peptide [Aβ] plaques, which progressively leads to neuronal cell death and decline in cognitive functions. Connections between autophagy and AD originate from the observation of expansion of autophagic compartments in AD brains (Nixon *et al*, 2005). As recently revealed by multilayer brain proteomics analysis performed at different stages of AD in humans, the autophagic substrate SQSTM1/p62 (sequestosome 1) accumulates in AD, suggestive of impaired autophagic flux (Bai *et al*, 2020) similar to the one reported in AD experimental models (Yu *et al*, 2005). In support of this notion, functional autophagy is required to degrade soluble and aggregated variants of MAPT/tau (Berger *et al*, 2006; Silva *et al*, 2020). Lysosomal membrane lesions caused by MAPT/tau oligomers instigate an LGALS3 (galectin 3)‐coordinated program, which leads to autophagy activation (Jia *et al*, 2020). Genetic inactivation of SQSTM1/p62 in mice leads to accumulation of hyperphosphorylated MAPT/tau and neurodegeneration (Ramesh Babu *et al*, 2008). Supraphysiological accumulation of MAPT/tau tangles perturbs the retrograde axonal transport of autophagosomes by interfering with the dynein–DCTN (dynactin) complex, eventually instigating the detrimental accumulation of MAPT/tau‐containing autophagic vesicles (Butzlaff *et al*, 2015).

Notably, the NFE2L2/NRF2 (nuclear factor, erythroid‐derived 2, like 2)‐dependent transcription of the autophagy regulator CALCOCO2/NDP52 (calcium binding and coiled‐coil domain 2) is instrumental in promoting the degradation of MAPT/tau in response to oxidative stress (Jo *et al*, 2014). SQSTM1/p62 is also a target gene for NFE2L2/NRF2 (Jain *et al*, 2010), and it has been reported to mediate degradation of aggregated MAPT/tau (Xu *et al*, 2019b). In recent years, dysfunction of the endosomal‐sorting complex, the retromer, has been linked to a number of neurodegenerative diseases, including AD. Reduced expression of the retromer proteins and variants of the core retromer component VPS35 (vacuolar protein sorting 35) are associated with neurodegenerative diseases, often overlapping with MAPT/tau aggregation in the brain (Carosi *et al*, 2021; Seaman, 2021). Recent data demonstrate that the autophagy–lysosomal axis is central for the clearance of aggregated MAPT/tau and depletion of VPS35 blocks autophagy, whereas VPS35 overexpression has the opposite effect (Carosi *et al*, 2020; Carosi *et al*, 2021). Thus, the retromer–autophagy axis may play a relevant function in preventing multiple neurodegenerative diseases by ensuring that pathogenic protein aggregates are cleared as they arise.

In addition, multitiered connections have been established between autophagy and Aβ plaque formation. Aβ is targeted for autophagy‐dependent degradation within the lysosome, explaining why activation of autophagy reduces the burden of Aβ plaques in rodents (Boland *et al*, 2008; Menzies *et al*, 2017; Meng *et al*, 2019). However, autophagy appears to be causally implicated in the PSEN1 (presenilin 1)‐mediated conversion of APP (amyloid beta precursor protein) into Aβ (Yu *et al*, 2005), as well as in the non‐canonical secretion of Aβ into the extracellular space (Nilsson *et al*, 2013; Menzies *et al*, 2017). Mutations that alter PSEN1 function have been associated with defective autophagic vesicle clearance and early‐onset AD, due to impaired autophagosome–lysosome fusion and defective lysosomal acidification (Lee *et al*, 2010b; Chong *et al*, 2018). Similarly, loss‐of‐function mutations affecting PICALM (phosphatidylinositol‐binding clathrin assembly protein) impair autophagy dynamics, thus augmenting the risk for developing AD (Tian *et al*, 2013).

Additional autophagy modulators determine the cellular levels of Aβ protein. As an example, NRBF2 (nuclear receptor‐binding factor 2; a component of the PtdIns3K complex I) interacts with APP and favors its lysosomal disposal, as demonstrated by the fact that NRBF2 depletion leads to excessive levels of intracellular APP in cells (Yang *et al*, 2017b) and Aβ accumulation in AD mouse models (Lachance *et al*, 2019), whereas overexpression of NRBF2 reduces Aβ levels and improves mouse memory (Lachance *et al*, 2019). Recently, a possible link between autophagy activation in the microglial compartment and AD has been proposed. Importantly, ablation of the gene coding for TREM2 (triggering receptor expressed on myeloid cells 2), a surface receptor required for microglial responses to neurodegeneration, results in maladaptive accumulation of autophagosomes and disarray of microglia clustering around plaques (Ulland *et al*, 2017). This effect has been attributed to dysregulated MTORC1 activation, in turn evoking metabolic abnormalities in microglial cells. Consistent with this notion, normalization of autophagic flux by cyclocreatine decreases neuronal dystrophy in murine models of AD (5XFAD mice) (Ulland *et al*, 2017). In this landscape, defective mitophagy appears to be a major determinant of the functional decay of neurons in AD, in that its pharmacological stimulation (through NAD^+^ supplementation, urolithin A, and actinonin) is sufficient to retard memory impairment, while reducing the burden of amyloid aggregates upon stimulating microglial phagocytic capacity for extracellular Aβ plaques (Fang *et al*, 2019). In addition, non‐canonical functions of the ATG machinery in microglia contribute to alleviate the toxic effects associated with Aβ plaque deposition in the 5XFAD mouse model. Notably, the genetic ablation of *Atg5* or *Rubcn* (but not that of *Rb1cc1*) in myeloid cells correlates with exacerbated Aβ plaque formation and aberrant production of inflammatory cytokines, while contributing to accelerate neuronal decay and cognitive impairment. Mechanistically, ATG5 and RUBCN take part in events of MAP1LC3/LC3 (microtubule‐associated protein 1 light chain 3) conjugation to Aβ–containing endosomal membranes positively marked by RAB5 and clathrin. This process, named LC3‐associated endocytosis (LANDO), appears to promote the recycling of putative Aβ receptors (e.g., TLR4, TREM2 [triggering receptor expressed on myeloid cells 2]) from internalized endosomes to the plasma membrane of microglial cells. While it remains to be clarified whether LANDO mediates Aβ receptor degradation, its activation is instrumental to reduce Aβ burden and limit neuroinflammation in AD (Heckmann *et al*, 2019). Along similar lines, LANDO deficiency imposed on aged mice by deletion of the WD domain of ATG16L1 (which is dispensable for canonical autophagy), exacerbates the neuroinflammatory phenotype associated with an AD‐like symptomatology (Heckmann *et al*, 2020).

Chaperone‐mediated autophagy also contributes to degradation of a large fraction of neuronal MAPT/tau under physiological conditions (Caballero *et al*, 2018; Caballero *et al*, 2021). However, mutations and posttranslational modifications of this protein, such as acetylation, not only prevent MAPT/tau degradation by CMA but also inhibit normal CMA functioning (Caballero *et al*, 2018; Caballero *et al*, 2021). Blockage of CMA leads to rerouting of some of the pathogenic forms of MAPT/tau toward endosomal microautophagy, as both pathways share the same chaperone, HSPA8, and this promotes fusion of late endosomes with the plasma membrane and subsequent extraneuronal release of the MAPT/tau variants, thus contributing to MAPT/tau propagation (Caballero *et al*, 2021). Reduction in neuronal CMA activity has been recently shown in AD patient's brains (Bourdenx *et al*, 2021; Caballero *et al*, 2021), and pharmacological activation of CMA has been linked to ameliorated pathology in two different experimental models of tauopathies (Bourdenx *et al*, 2021).

### Parkinson disease

Parkinson disease (PD) is pathologically defined by (i) the loss of dopaminergic neurons in the substantia nigra (SN) and (ii) the prevalence of proteinaceous Lewy bodies, mainly composed of SNCA/α‐synuclein (synuclein alpha) and other polyubiquitinated proteins but also vesicular structures. PD symptomatology is characterized by prominent motor and autonomic dysfunction, sometimes accompanied by cognitive and psychological deficits. Early evidence suggested roles for CMA and macroautophagy in degrading SNCA/α‐synuclein (Webb *et al*, 2003; Cuervo *et al*, 2004). High expression of wild‐type SNCA/α‐synuclein, mutations or unwanted posttranslational modifications on this protein (such as formation of dopamine adducts) is toxic to CMA by preventing multimerization of LAMP2A and subsequent lysosomal internalization of cargo proteins (Cuervo *et al*, 2004; Martinez‐Vicente *et al*, 2008). Recent evidence has demonstrated that selective autophagy clears neuron‐released SNCA/α‐synuclein through the autophagy receptor SQSTM1/p62 in microglia, offering protection of dopaminergic neurons (Choi *et al*, 2020). Consistent with this result, the activation of autophagy decreases the accumulation of SNCA/α‐synuclein (Nakamura *et al*, 2019). Conversely, uncontrolled expression of wild‐type or mutated variants of SNCA/α‐synuclein reduces autophagic flux or disturbs TFEB‐mediated lysosomal biogenesis by preventing the nuclear translocation of TFEB (Decressac *et al*, 2013). Pathologically meaningful levels of SNCA/α‐synuclein affect the intracellular localization of ATG9 via RAB1A (RAB1A, member RAS oncogene family), thereby perturbing autophagy dynamics in the brain of transgenic mice overexpressing SNCA/α‐synuclein (Winslow *et al*, 2010). Mutations in the gene *GBA* (glucosylceramidase beta) represent the most common genetic risk factor for PD. Of note, loss‐of‐function mutations in *GBA* disrupt the autophagic flux and lead to the aggregation of SNCA/α‐synuclein (Murphy *et al*, 2014). Likewise, an autosomal‐dominant mutation affecting VPS35 curtails autophagy by altering ATG9 localization (Zavodszky *et al*, 2014). A similar phenotype has also been described in the context of loss‐of‐function mutations in the P‐type ATPase gene *ATP13A2*, in which recessive, early‐onset PD has been linked to defective acidification of lysosomes and insufficient autophagy (Ramirez *et al*, 2006). Decreased autophagy in ATP13A2‐deficient neurons in turn leads to accumulation of damaged mitochondria with increased leakage of reactive oxygen species (ROS) (Gusdon *et al*, 2012).

Dysregulated autophagy has also been associated with the expression of dominant mutants of LRRK2 (leucine‐rich repeat kinase 2) (Ramonet *et al*, 2011), the most common cause of familial PD. While it remains controversial whether LRRK2^G2019S^ elicits increased or decreased autophagic flux, these differences may reflect the compartment (soma vs. dendrites vs. axons) being studied. Although autophagy upregulation may contribute to clearance of protein aggregates, the axo‐dendritic arbor is susceptible to autophagy‐mediated degeneration in cultured dopaminergic, sympathetic, and cortical neurons and in the axons of dopaminergic neurons *in vivo* as evidenced by *Atg7* knockdown/knockout (Plowey *et al*, 2008; Cheng *et al*, 2011), expression of dominant‐negative ULK1 (Balke *et al*, 2020), or expression of an autophagy‐deficient LC3 phosphomimic, which protects against dendritic atrophy elicited by disease‐linked LRRK2 mutations and the PD toxin MPP^+^ (Cherra *et al*, 2010). Increased mitophagy, due to post‐synaptic mitochondrial calcium dysregulation, may contribute to dendritic degeneration (Verma *et al*, 2017). Emerging roles for LRRK2 in regulating RAB GTPases and other aspects of endolysosomal and vesicular transport may also complicate interpretation due to compensatory responses (Kuwahara & Iwatsubo, 2020).

A causal association has been established between autosomal recessive forms of PD and mutations affecting the mitophagy regulators PINK1 (PTEN‐induced putative kinase 1) and PRKN/PARK2 (Parkin RBR E3 ubiquitin protein ligase) (Kitada *et al*, 1998; Valente *et al*, 2004; Narendra *et al*, 2008; Matsuda *et al*, 2010). Mouse models to monitor mitophagy show elevated basal mitophagy in dopaminergic neurons (McWilliams *et al*, 2018). Although PINK1 (McWilliams *et al*, 2018) and PRKN (Goldberg *et al*, 2003; Perez & Palmiter, 2005) deficiency do not elicit major defects under baseline conditions, defective striatal neural plasticity is observed in *prkn^−^* ^/^^−^ mice (Kitada *et al*, 2009). Importantly, mitophagy deficiency favored by ablation of *Prkn* (Palacino *et al*, 2004; Pickrell *et al*, 2015) or *Pink1* (Gautier *et al*, 2008) sensitizes mice to oxidative stress, while worsening neural damage when combined with mitochondrial dysfunction (mitochondrial DNA [mtDNA] mutator‐*prkn*/*parkin*‐KO mice) (Pickrell *et al*, 2015). However, there are other pathways of mitophagy in neurons (Chu *et al*, 2013), and ablation of *Pink1* or *Prkn* in mouse and fly mitophagy biosensor models suggests that neither protein is necessary to maintain normal basal levels of brain mitophagy (Lee *et al*, 2018a; McWilliams *et al*, 2018). Furthermore, serological markers of inflammation, which are also observed in individuals with *Prkn* mutations, are reduced leading to reversal of neuronal degeneration when these mice are crossed to STING1/STING (stimulator of interferon response cGAMP interactor 1)‐deficient mice (Sliter *et al*, 2018). These results match the original observation indicating a close association between PD and serum or cerebrospinal fluid markers of inflammation, further reinforcing the concept that neuroinflammation directly contributes to the pathogenesis of PD (Dzamko *et al*, 2015).

### Polyglutamine diseases

Extensive experimental evidence has highlighted the role of autophagy in disorders caused by polyglutamine (polyQ) expansion, including Huntington disease (HD) and several forms of spinocerebellar ataxias (Jimenez‐Sanchez *et al*, 2012). The polyQ expansion in HTT (huntingtin) is the etiological driver of HD (Zheng *et al*, 2010), and the severity thereof is a direct function of polyQ length. Importantly, a significant dichotomy has emerged between the functions of wild‐type and mutated HTT toward the regulation of the autophagic process (Martin *et al*, 2015; Ashkenazi *et al*, 2017). Wild‐type HTT participates in the regulation of basal autophagy due to its role in the selection of the autophagic cargo (Ochaba *et al*, 2014; Rui *et al*, 2015). However, expression of mutant HTT (i) negatively affects autophagosomal cargo recognition through dysregulated interaction with SQSTM1/p62 (Martinez‐Vicente *et al*, 2010; Rui *et al*, 2015); (ii) sequesters the BECN1 interactor RASD2/RHES in the striatum (Mealer *et al*, 2014) and inhibits BECN1‐PIK3C3/VPS34 and ULK1 kinase activities (Lim *et al*, 2015; Wold *et al*, 2016); (iii) interferes with the regulatory interaction between ATXN3 (ataxin 3) and BECN1, compromising the response of neurons to starvation (Ashkenazi *et al*, 2017); (iv) disturbs axonal autophagosome transport (Wong & Holzbaur, 2014b); (v) drives a maladaptive unfolded protein response, which leads to ERN1/IRE1 (endoplasmic reticulum to nucleus signaling 1)‐dependent inhibition of autophagy (Lee *et al*, 2012); and (vi) disrupts the ability of wild‐type HTT to bind ULK1 and release it from the negative regulation of MTOR in order to activate autophagy (Rui *et al*, 2015). Notably, overexpression of wild‐type HTT in cells expressing its mutated variants restores autophagy and fosters the clearance of mutated HTT (Zheng *et al*, 2010). Of note, defective autophagy imposed by heterozygous depletion of the autophagy scaffold/adaptor WDFY3/ALFY (WD repeat and FYVE domain containing 3) accelerates the onset (and worsens the sequelae) of HD in mice (Fox *et al*, 2020). Interestingly, experimental rerouting of mutant HTT for degradation by CMA has proven effective in ameliorating disease phenotype in mice (Bauer *et al*, 2010).

### Neuropathies

Neuropathies are disorders caused by the progressive degeneration and death of peripheral sensory (e.g., hereditary sensory and autonomic neuropathy [HSAN]) and motor (hereditary spastic paraplegia [HSP], Spastic paraplegia type 49 [SPG49]) neurons. Mutations in genes encoding several ER proteins involved in ER‐remodeling have been associated with hereditary neuropathies (Hubner & Dikic, 2020). For example, loss‐of‐function mutations in the reticulon type ER membrane protein RETREG1/FAM134B (reticulophagy regulator 1) are associated with the development of HSAN type II (HSAN2) (Kurth *et al*, 2009; Murphy *et al*, 2012), whereas mutations in RTN2 (reticulon 2) are linked with HSP (SPG12) (Montenegro *et al*, 2012). RETREG1 was identified as the first mammalian receptor for selective ER autophagy (reticulophagy) implicated in the delivery of ER fragments via autophagosomes for lysosomal degradation (Khaminets *et al*, 2015). RETREG1 also plays a role in the clearance of ER‐to‐lysosome‐associated degradation (ERAD)‐resistant SERPINA1/alpha‐1 antitrypsin Z variant polymers (Fregno *et al*, 2018) as well as endogenous procollagen (Forrester *et al*, 2019) within the ER. Some patients with mutations in RETREG1 suffer from cardiac arrhythmia, an‐ or hypohydrosis and other symptoms of autonomic malfunctions overlapping with amyotrophic lateral sclerosis (ALS) and myopathies (Eggermann *et al*, 2018). The HSAN‐related ATL3 (atlastin GTPase 3) Y192C mutation has been connected to reduced complexity of the endoplasmic reticulon network, disturbed connections between ER and mitochondria, and impaired mitochondrial function (Kornak *et al*, 2014; Behrendt *et al*, 2019; Krols *et al*, 2019; Xu *et al*, 2019a). Mutations in ATL1 paralog can also result in autosomal‐dominant spastic paraplegia (SPG3) (Zhao *et al*, 2001) or in HSAN type I (HSAN1) (Guelly *et al*, 2011). Atlastins in general are thought to remodel the ER for efficient autophagosomal degradation and functioning downstream of the reticulophagy receptor RETREG1 (Liang *et al*, 2018). As a caveat, it is worth mentioning that ATL1 and ATL3 are implicated in multiple ER‐related pathways. Therefore, additional studies are required to validate the hypothesis that dysfunctional autophagy primarily contributes to the phenotypic aberrations associated with mutations affecting these genes.

Spastic paraplegia type 49 (SPG49) is a severe neurodegenerative disorder that starts in infancy and is caused by several mutations in the *TECPR2* (tectonin beta‐propeller repeat containing 2) gene. Frame‐shift mutations in exon 8 and exon 16 of *TECPR2* (c.1319delT, c.3416delT) terminate in a premature stop codon (Oz‐Levi *et al*, 2012; Heimer *et al*, 2016), and an initial link between this gene to defects in autophagy was reported (Behrends *et al*, 2010; Oz‐Levi *et al*, 2012). All SPG49 patients share unique dysmorphic features such as microcephaly, dental crowding, short chubby appearance and a short, broad neck, and suffer from evolving spasticity, moderate to severe intellectual disability, decreased pain sensitivity and infantile onset of chronic respiratory disease (Oz‐Levi *et al*, 2012; Heimer *et al*, 2016). TECPR2 is a multi‐domain protein comprised of three WD repeats at the N terminus, the mostly unstructured middle region and six TECPR2 repeats terminating with an LC3‐interacting region (LIR) motif at its C terminus (Behrends *et al*, 2010; Stadel *et al*, 2015). TECPR2 was originally identified as an interactor of the Atg8‐family proteins, a detailed interactome of TECPR2 validated its interaction with Atg8‐family proteins through its functional LIR motif, and in addition identified its interaction with the biogenesis of lysosomal organelles complex 1 (BLOC1) and the homotypic fusion and protein sorting (HOPS) complex, two tethering protein complexes that mediate autophagosome–lysosome fusion (Stadel *et al*, 2015). A model for SPG49 was recently developed by creating a *tecpr2* knockout mouse using CRISPR‐Cas9 (Tamim‐Yecheskel *et al*, 2020). This mouse exhibits behavioral aberrations accompanied by neuroaxonal dystrophy and autophagosome accumulation in the brainstem and spinal cord that is exacerbated in an age‐dependent manner. The accumulation of autophagosomes upon *tecpr2* knockout suggests compromised targeting to lysosomes. Consistently, SPG49‐derived primary skin fibroblasts also exhibit accumulation of autophagosomes, strictly under basal growing conditions (Fraiberg *et al*, 2020). This phenotype is recovered by ectopically expressing the six carboxy‐terminal TECPR2 repeats, the full length TECPR2 protein or by inhibition of MTOR (Fraiberg *et al*, 2020). Mechanistically, TECPR2 has been suggested to facilitate targeting of autophagosomes to lysosomes, a process that is dependent on its C‐terminal LIR motif.

Recent studies of rare movement disorders have also provided links to autophagy. *VPS13D* is a rare disease gene, with mutations in *VPS13D* being associated with pediatric and young adult spastic ataxia or spastic paraplegia (Gauthier *et al*, 2018; Seong *et al*, 2018). Significantly, VPS13D is a regulator of autophagy, mitochondrial size, and mitochondrial clearance (Anding *et al*, 2018). These cellular phenotypes appear to be caused by altered mitochondria and ER contact, a phenotype that is conserved between flies and patient‐derived cells (Shen *et al*, 2021). Furthermore, a recent study indicated that mutations in VPS13D occur in 3 out of 64 children with Leigh syndrome features (Lee *et al*, 2020).

Further, a very recent study has identified a novel role for TRK‐fused gene (TFG) in autophagy (Carinci *et al*, 2021). TFG is an essential protein in the regulation of vesicular trafficking between endoplasmic reticulum and Golgi, and several *TFG* mutations have been associated with different neurological disorders, including hereditary motor and sensory neuropathy with proximal dominant involvement (HMSN‐P), Charcot‐–Marie–Tooth disease, and recessive hereditary spastic paraparesis (Yagi *et al*, 2016). Indeed, under starvation conditions, TFG controls proper ULK1 localization and steady‐state levels by interacting with LC3C *via* a canonical LIR motif; this, in turn, regulates autophagy progression. These defects are also recapitulated in fibroblasts from a patient carrying an R106C TFG variant that has been previously associated with a complicated hereditary spastic paraplegia (HSP) phenotype (Beetz *et al*, 2013).

### Amyotrophic lateral sclerosis

Amyotrophic lateral sclerosis is etiologically associated with the aberrant amassing of misfolded proteins, including SOD1 (superoxide dismutase 1), TARDBP/TDP‐43 (TAR DNA binding protein), or with the translation of dipeptide repeat proteins from the C9orf72 expanded repeat (the latter accounting for the most common variant of ALS) in motor neurons. ALS forms a genetic and pathological continuum with frontotemporal dementia (FTD). Interestingly, several FTD‐ALS genes code for autophagy receptors, including SQSTM1/p62 and OPTN (optineurin), lowering the capacity of neural cells to clear protein aggregates, as do mutations in VCP (valosin containing protein). As an example, SQSTM1/p62 mutants fail to dispose of aggregation‐prone SOD1 and TARDBP (Gal *et al*, 2009; Brady *et al*, 2011; Goode *et al*, 2016; Deng *et al*, 2020). Likewise, defective OPTN, leading to impaired binding to MYO6 (myosin VI), compromises autophagosomal trafficking (Tumbarello *et al*, 2012; Wong & Holzbaur, 2014a). Further supporting the role of OPTN in ALS, mutations in *TBK1* (TANK binding kinase 1), which phosphorylates OPTN and promotes mitophagy, lead to detrimental accumulation of damaged mitochondria (Moore & Holzbaur, 2016). Of note, loss of TBK1 activity in SOD1^G93A^ mouse models of ALS curtails autophagy and accelerates the clinical manifestation of ALS (Gerbino *et al*, 2020).

The strict nexus between ALS and autophagy is further strengthened by experimental evidence indicating that genetic deletion of central (e.g., VCP) (Johnson *et al*, 2010) or ancillary regulators of the autophagic cascade (e.g., GRN/progranulin, ALS2/alsin‐2) precipitate ALS symptomatology in mice and human patients (Yang *et al*, 2001; Chang *et al*, 2017). VCP also cooperates with PINK1 in regulating mitophagy and promoting PINK1‐dependent neuronal dendritogenesis through an independent mechanism (Kim *et al*, 2013b; Wang *et al*, 2018b). Mutations in the ESCRT‐III subunit CHMP2B (charged multivesicular body protein 2B)—required to sort integral membrane proteins into intraluminal vesicles of the multivesicular body (MVB)—have been causally linked to frontotemporal dementia and ALS. Mechanistically, mutated CHMP2B undermines autophagy‐mediated degradation, resulting in an elevated burden of SQSTM1/p62‐ and WDFY3‐containing protein aggregates in neurons. Further corroborating the central role of MVBs in the maintenance of neuronal proteostasis, MVBs are essential for the clearance of ubiquitinated TARDBP, which accumulates in ALS and frontotemporal lobar degeneration (Filimonenko *et al*, 2007). Mitophagy also appears to be defective in ALS (Wong & Holzbaur, 2014a). As result and in a non‐mutually exclusive manner, an impairment of ESCRT‐III function in phagophore sealing during mitophagy could contribute the ALS pathophysiology (Smith *et al*, 2019; Zhen *et al*, 2020). While these experimental observations suggest that defective autophagy may directly contribute to the phenotypic alterations linked to mutations in these genes, the fact that these proteins are involved in several autophagy‐unrelated processes imposes a note of caution on the interpretation of these results.

Conversely, genetic interventions that promote autophagy, such as the inactivation of the transcription factor XBP1 (X‐box binding protein 1) or restoration of HSPB8 expression in the nervous system, counteract ALS symptomatology by promoting the autophagy‐dependent disposal of SOD1^G93A^ (Hetz *et al*, 2009; Crippa *et al*, 2010). Mutated forms of C9orf72 lead to the clinical manifestation of ALS through a number of different mechanisms. Because wild‐type C9orf72 is involved in central aspects of autophagosomes formation, maturation, and trafficking, it is likely that perturbations in autophagy contribute to the detrimental action of mutated C9orf72 in motor neuron dysfunction (Webster *et al*, 2016; Ho *et al*, 2019). Supporting this notion, genetic ablation of *C9orf72* correlates with an increased burden of SQSTM1/p62 and TARDBP protein aggregation and synergizes with polyQ ATXN2 to induce the demise of motor neurons (Sellier *et al*, 2016). Consistently, it has been recently observed that loss of wild‐type C9orf72 function exacerbates the neurotoxic effects of a *C9orf72* mutant allele, bearing hexanucleotide expansions, by repressing autophagy (Zhu *et al*, 2020). Conversely, the unexpected increase in lifespan elicited by BECN1 haploinsufficiency in the mutant SOD1 transgenic mouse model of ALS (Nassif *et al*, 2014) is difficult to reconcile. As for all the diseases discussed in this review, apparently conflicting, context‐dependent conclusions indicate a nuanced relationship between autophagy dysregulation and neurodegeneration.

## Cardiovascular diseases

Cardiovascular disorders represent the leading cause of death worldwide. Cardiomyocytes, the essential cellular constituents of the cardiovascular system, mostly lay in the post‐mitotic state, implying that they are highly dependent upon intact autophagy and mitophagy to preserve their physiological functions and cope with harmful insults (Lavandero *et al*, 2015; Kaludercic *et al*, 2020) (Table 2). In view of the reduced regenerative potential of the cardiovascular system, autophagy operates at the forefront to promote survival of quiescent cells in the cardiovascular compartment, while counteracting events of apoptotic or necrotic cell death after injury (Henning & Brundel, 2017; Sciarretta *et al*, 2018).

**Table 2:** Cardiovascular diseases associated with genetic intervention of autophagy in mice.

Setting	Genetic intervention	Effects on disease phenotype	Ref.
Atherosclerosis	Macrophage‐specific deletion of *Atg5*	Enhanced atherogenic plaque progression due to hyperactivation of macrophage‐mediated inflammation and impaired lipid droplets catabolism	Ouimet *et al* (2011), Liao *et al* (2012), Razani *et al* (2012)
Atherosclerosis	Macrophage‐specific deletion of *Rptor*	Reduced development of atherogenic plaque upon high protein diet after restoration of mitophagy in macrophages	Zhang *et al* (2020)
Atherosclerosis	Macrophage‐specific overexpression of *Tfeb*	Reduced development of atherogenic plaque after stimulation of lysosomal biogenesis in macrophages	Sergin *et al* (2017)
Atherosclerosis	Vascular smooth muscle cell‐specific deletion of *Atg7*	Enhanced atherogenic plaque progression, linked to increased CCL2‐mediated macrophage recruitment	Osonoi *et al* (2018)
Atherosclerosis	Endothelial cell‐specific deletion of *Atg7* or *Atg5*	Enhanced atherogenic plaque progression in hypercholesterolemic mice, linked to endothelial apoptosis, senescence, and inflammation	Vion *et al* (2017)
Atherosclerosis	Macrophage‐specific deletion of *Rptor*	Decreased atherogenic plaque formation with concomitant reductions in plaque macrophage content in *Apoe^−^* ^/^^−^ mice	Zhang *et al* (2020)
Cardiomyopathies	Conditional cardiomyocyte‐specific deletion of *Atg5*	Exacerbated cardiac abnormalities and premature death, linked to increased ubiquitination and mitochondrial misalignment	Nakai *et al* (2007), Taneike *et al* (2010), Eisenberg *et al* (2016)
Cardiomyopathies	Whole‐body allelic loss of *Atg5*	Exacerbated Ang‐II‐induced cardiac hypertrophy, linked to increased ROS production and NF‐κB activation in macrophages	Zhao *et al* (2014)
Cardiomyopathies	Cardiomyocyte‐specific overexpression of miR‐212/132	Pathological cardiac hypertrophy, heart failure, and premature death, after impaired autophagic response upon starvation	Ucar *et al* (2012)
Cardiomyopathies	Cardiomyocyte‐specific overexpression of miR‐199a	Pathological cardiac hypertrophy, heart failure and premature death, after impaired autophagic response upon starvation	Li *et al* (2017)
Cardiomyopathies	Cardiomyocyte‐specific knock‐in of mutant *TSC2^S1365A^*	Exacerbated cardiac hypertrophy and premature death from sustained PO after mTORC1 hyperactivation	Ranek *et al* (2019)
Cardiomyopathies	Cardiomyocyte‐specific deletion of *Tsc2*	Exacerbated cardiac hypertrophy and premature death after mTORC1 hyperactivation	Taneike *et al* (2016)
Cardiomyopathies	Whole‐body deletion of *Lamp2*	Accelerated development of a vacuolar cardioskeletal myopathy similar to Danon disease	Nishino *et al* (2000), Tanaka *et al* (2000)
Cardiomyopathies	Whole‐body deletion of *Fbxo32*	Development of severe cardiomyopathy, with interstitial fibrosis, reduced diastolic function, and arrhythmias, after impaired autophagy	Zaglia *et al* (2014)
Cardiomyopathies	Conditional cardiomyocyte‐specific overexpression of *Atg7*	Ameliorated signs of desmin‐related cardiomyopathy and prolonged survival after autophagy activation in *CryAB^R120G^* Mice	Bhuiyan *et al* (2013)
Cardiomyopathies	Whole‐body allelic loss of *Becn1*	Exacerbated signs of desmin‐related cardiomyopathy and reduced survival after autophagy inhibition in *CryAB^R120G^* Mice	Bhuiyan *et al* (2013)
Cardiomyopathies	Whole‐body deletion of *Tp53*	Decelerated cardiac aging, linked to improved mitophagic responses after stabilization of PRKN	Hoshino *et al* (2013)
Cardiomyopathies	Conditional cardiomyocyte knock‐in of mutant *MNF2^AA^*	Development of perinatal cardiomyopathy and premature death, after inhibition of mitochondrial PRKN translocation at birth	Gong *et al* (2015)
Cardiomyopathies	Conditional cardiomyocyte‐specific deletion of *Prkn*	Development of perinatal cardiomyopathy and premature death, linked to impaired mitochondrial biogenesis	Gong *et al* (2015)
Cardiomyopathies	Whole‐body deletion of *Pink1*	Left ventricular dysfunction and cardiac hypertrophy by 2 months of age, linked to mitochondrial dysfunction	Billia *et al* (2011)
Cardiomyopathies	Cardiomyocyte‐specific co‐deletion of *Bnip3* and *Bnip3l*	Cardiac hypertrophy and contractile dysfunction, linked to atypical mitochondrial morphology	Dorn (2010)
Cardiomyopathies	Cardiomyocyte‐specific deletion of *Mnf2*	Progressive cardiomyopathy due to accumulation of morphologically and functionally abnormal mitochondria	Chen and Dorn (2013)
Cardiomyopathies	Conditional cardiomyocyte‐specific co‐deletion of *Mnf2* and *Mnf1*	Impaired myocardial contractile function due to malfunctional mitochondria, but protection against acute myocardial infarction	Hall *et al* (2016)
Cardiomyopathies	Cardiomyocyte‐specific deletion of *Dnase2a*	Left ventricular dilatation, severe contractile dysfunction, inflammation and premature death from sustained PO, linked to mitochondrial misalignment and aggregation	Oka *et al* (2012)
IRI	Whole‐body allelic loss of *Becn1*	Reduced size of myocardial infarction/area after IRI But: Exacerbated ischemic damage upon HFD and resistance to rapamycin	Matsui *et al* (2007), Sciarretta *et al* (2012)
IRI	Conditional cardiomyocyte‐specific deletion of *mTORC1*	Exacerbated hypoxic injury and cardiomyocyte apoptosis after autophagy restoration	Sciarretta *et al* (2012)
IRI	Conditional cardiomyocyte‐specific overexpression of *Rheb*	Exacerbated hypoxic injury and cardiomyocyte apoptosis after autophagy restoration	Sciarretta *et al* (2012)
IRI	Whole‐body deletion of *Mst1*	Reduced myocardial infarction after autophagy restoration	Maejima *et al* (2013)
IRI	Cardiomyocyte‐specific overexpression of DN‐Mst1	Reduced myocardial infarction after autophagy restoration	Maejima *et al* (2013)
IRI	Whole‐body deletion of *Pgam5*	Exacerbated necroptosis and ischemic injury after inhibition of mitophagy and accumulation of abnormal mitochondria	Lu *et al* (2016)
IRI	Conditional cardiomyocyte‐specific deletion of *Dnm1l*	Exacerbated size of myocardial infarction/area after inhibition of mitophagy	Cahill *et al* (2015), Ikeda *et al* (2015)
IRI	Whole‐body deletion of *Prkn*	Exacerbated size of myocardial infarction/area and reduced survival, after inhibition of mitophagy	Kubli *et al* (2013)
IRI	AAV‐mediated deletion of Atg7 with Mir188‐3p)	Reduced size of myocardial infarction/area	Wang *et al* (2015)
IRI	Cardiac‐specific overexpression of DN‐GSK‐3β	Exacerbated size of myocardial infarction/area after prolonged ischemia, after autophagy activation	Zhai *et al* (2011)
IRI	Cardiomyocyte‐specific deletion of *Rubcn*	Reduced IRI linked to autosis inhibition after restoration of normal autophagic flux	Nah *et al* (2020)

AAV, adeno‐associated viral vector; Ang‐II, angiotensin II; DN, dominant negative; IRI, ischemia‐reperfusion injury; PO, pressure overload; ROS, reactive oxygen species.

### Cardiomyopathies

As best illustrated by the genetic inhibition of essential or ancillary genes within the ATG machinery, autophagy deficiency renders mice prone to develop early‐onset cardiomyopathies, either under basal conditions or upon pre‐pathological circumstances of stress (e.g., pressure overload) (Bravo‐San Pedro *et al*, 2017). Consistently, mice with a cardiomyocyte‐specific conditional inactivation of *Atg5*, and challenged with transverse aortic constriction, display defects in sarcomere structure, aberrant aggregation of misfolded proteins, and altered mitochondrial dynamics, followed by prominent cardiac abnormalities (contractile dysfunction, maladaptive hypertrophy, left ventricular dilation) and early mortality (Nakai *et al*, 2007; Taneike *et al*, 2010). Likewise, the deletion of a single copy of *Atg5* worsens angiotensin II‐induced cardiac hypertrophy (Zhao *et al*, 2014; Bravo‐San Pedro *et al*, 2017). Along similar lines, the cardiomyocyte‐specific overexpression of miRNAs invalidating the transcriptional activity of FOXO3 (Ucar *et al*, 2012) or activating MTORC1 (Li *et al*, 2017) precipitates cardiac function, leading to heart failure. In addition, broad‐spectrum autophagic defects tied to the systemic ablation of LAMP2 (causing Danon disease) account for the early development of hypertrophic cardiomyopathy (Nishino *et al*, 2000; Tanaka *et al*, 2000). In this scenario, the persistent activation of MTORC1 lowers the capacity of cardiomyocytes to sustain pressure overload‐induced stress, as testified to by the fact that mice bearing knock‐in mutation in the MTORC1 inhibitor *Tsc2* (TSC complex subunit 2) develop heart disease (Taneike *et al*, 2016), while succumbing to pressure overload (Ranek *et al*, 2019).

The detrimental effects associated with the inactivation of autophagy in cardiomyocytes are largely due to its involvement in the regulation of proteostatic adaptations and in the maintenance of mitochondrial fitness. Thus, the genetic knockout of the muscle‐specific ubiquitin ligase *Fbxo32*/*atrogin‐1* (F‐box protein 32) prevents the proteasomal degradation of the autophagy regulator CHMP2B, possibly resulting in insufficient autophagic flux and aberrant protein aggregation, which are etiologically associated with the development of severe cardiomyopathy (Zaglia *et al*, 2014). Similarly, the overexpression of ATG7 ameliorates signs of DES (desmin)‐related cardiomyopathy in mice expressing the R120G mutant of CRYAB (crystallin, alpha B) (Bhuiyan *et al*, 2013), whereas the heterozygous loss of *Becn1* accelerates heart failure under the same pathological setting (Tannous *et al*, 2008). However, defective mitophagy calls for major cardiac abnormalities. In particular, *Trp53* (transformation‐related protein 53, for simplicity referred to as TP53) whole‐body deletion restrains the age‐dependent decline in cardiac performance by promoting the stabilization of the central mitophagy regulator PRKN (Hoshino *et al*, 2013). Accordingly, (i) cardiomyocyte‐restricted deletion of *Prkn* at birth (but not after weaning) hastens the manifestation of cardiac hypertrophy (Gong *et al*, 2015); (ii) whole‐body knockout of *Pink1*, another modulator of mitophagy, links to left ventricular defects and compensatory cardiac hypertrophy (Billia *et al*, 2011); and (iii) simultaneous deletion of genes coding for the mitophagy regulators BNIP3 (BCL2/adenovirus E1B interacting protein 3) and BNIP3L (BCL2/adenovirus E1B interacting protein 3‐like) leads to cardiac hypertrophy and impaired contractile functions, tied to ultrastructural mitochondrial alterations (Dorn, 2010).

Further highlighting the central role of proficient mitophagy in cardiac homeostasis, cardiomyocyte‐specific ablation of the gene encoding the PRKN regulator MFN2 (mitofusin 2) phenotypically manifests as lethal cardiomyopathy associated with insufficient mitophagy (Chen & Dorn, 2013), and co‐deletion of *Mfn1* and *Mfn2* in adult cardiomyocytes compromises optimal mitochondrial fusion, igniting dilated cardiomyopathy and heart failure (Hall *et al*, 2016). Moreover, mice lacking *Dnase2* (deoxyribonuclease II alpha), a gene coding for a lysosomal enzyme that catalyzes the autophagy‐dependent degradation of DNA released from damaged mitochondria), display major cardiac alterations when challenged with protocols of pressure overload (Oka *et al*, 2012). Finally, PINK1‐mediated mitophagy and PRKN‐mediated mitophagy are defective in the hearts of Duchenne muscular dystrophy model mice (Kang *et al*, 2018). Taken together, these data lay significant emphasis on the primordial role of autophagy in the safeguard of cardiovascular homeostasis. This concept is further reinforced by the demonstration that pharmacological preclinically harnessed to correct cardiovascular dysfunctions (e.g., spermidine, rapamycin) cannot prescind from intact autophagy to mediate their pro‐health effects (Sciarretta *et al*, 2012; Eisenberg *et al*, 2016).

### Ischemia‐reperfusion injury

Pathological episodes that lead to the occlusion of coronary arteries impose on cardiomyocytes ischemic stress, peculiarly defined by temporally limited shortage of nutrients and exacerbated production of ROS, followed by a (mal)adaptive phase of reperfusion. Extensive evidence supports the view that autophagy is etiologically implicated in settings of ischemia‐reperfusion injury (IRI) (Martins *et al*, 2011; Lavandero *et al*, 2015; Bravo‐San Pedro *et al*, 2017; Sciarretta *et al*, 2018; Kaludercic *et al*, 2020). For example, a prominent surge in the autophagic flux, paralleling the inhibition of MTORC1, which in turn follows the activation of AMP‐activated protein kinase (AMPK) or the inhibition of RHEB (Ras homolog enriched in brain), occurs upon ischemic injury (Matsui *et al*, 2007; Sciarretta *et al*, 2012). Consistently, mice engineered to restore RHEB and MTORC1 functions display exacerbated hypoxic injury and cardiomyocyte apoptosis, suggesting that functional autophagy equips cardiomyocytes with a superior capacity to sustain the ischemic shock (Sciarretta *et al*, 2012). Likewise, cardiac‐selective deletion of *Nox4* (NADPH oxidase 4), which impairs the autophagy response, aggravates the ischemic injury (Sciarretta *et al*, 2013). Conversely, mice lacking the pro‐apoptotic kinase MST1 show improved activation of cytoprotective autophagy and resistance to ischemic stress (Maejima *et al*, 2013).

In agreement with the notion that altered mitochondrial dynamics etiologically contribute to the ischemic damage, functional mitophagy appears to be required to support the survival of cardiomyocytes, presumably by limiting the burden of oxidative stress that accompanies the ischemic episode (Saito & Sadoshima, 2015; Bravo‐San Pedro *et al*, 2017). Consistently, whole‐body deletion of the mitophagy regulator *Pgam5* (phosphoglycerate mutase family member 5) worsens the pathological outcome of myocardial infarction, inasmuch as it promotes events of necroptotic cell death (Lu *et al*, 2016). Furthermore, the cardiomyocyte‐specific ablation of the mitochondrial fission regulator *Dnm1l*/*Drp1* (dynamin 1‐like) compromises optimal mitophagy and exacerbates the IRI (Cahill *et al*, 2015; Ikeda *et al*, 2015), and *prkn^−^* ^/^^−^ mice subjected to permanent ligation of the left descending cardiac artery exhibit more severe ischemic damage compared with their wild‐type littermates (Kubli *et al*, 2013). While these data lend robust support to the hypothesis that functional autophagy mitigates ischemic damage, this process appears to play a maladaptive role in the reperfusion phase, as demonstrated by the leading observation that *Becn1* ^+/−^ mice display enhanced resistance to reperfusion damage compared with their autophagy‐competent counterparts (Ma *et al*, 2012a; Ma *et al*, 2012b). Of note, this finding can be functionally recapitulated by (i) the downregulation of *Atg7* achieved via adenoviral delivery of *Mir188‐3p*, which appears to limit the size of myocardial infarction (Wang *et al*, 2015); and (ii) GSK3B (glycogen synthase kinase 3 beta) inhibition, which suppresses autophagy in an MTORC1‐dependent manner (Zhai *et al*, 2011). Conversely, it has been proposed that the accumulation of autophagosomes that defines the reperfusion stage may instead reflect defective autophagosomal clearance (Ma *et al*, 2012a; Ma *et al*, 2012b). The accurate assessment of the autophagy flux is hence instrumental to resolve this conundrum. In addition, IRI has been causally connected with autosis, a type of cell death ignited by the excessive activation of autophagy (Liu *et al*, 2013c). Autosis is upregulated during the reperfusion stage, alongside the enhanced expression of the negative autophagy regulator RUBCN, which results in the aberrant pile‐up of autophagosomes in cardiomyocytes (Nah *et al*, 2020). *De facto*, the genetic suppression of *RUBCN*, or the inhibition of autosis by treatment with cardiac glycosides, normalizes the autophagic flux and improves the response to IRI (Nah *et al*, 2020).

### Atherosclerosis

As suggested above, persistent nutritional imbalance or overindulgent lifestyle behaviors undermine basal autophagy, thereby accelerating the occurrence of metabolic disorders. Importantly, excessive calorie intake impairs cardiovascular autophagy, in part accounting for the accrued propensity to manifest diabetic cardiomyopathy and atherosclerosis. Supporting this finding, *Becn1^+^* ^/^^−^ mice receiving a high‐fat diet (HFD) exhibit heightened ischemic damage compared with wild‐type littermates in settings of prolonged ischemia (Sciarretta *et al*, 2012). Noteworthy, stimulation of BECN1‐dependent autophagy by physical exercise is sufficient to correct defects in the autophagic flux mediated by HFD feeding in cardiomyocytes (He *et al*, 2012).

Data obtained from preclinical models support the tenet that autophagy is a major disease‐modifying process during the different phases of atherogenesis (Martinet & De Meyer, 2009; Kaludercic *et al*, 2020). In *apoe* (apolipoprotein E)‐knockout mice fed a westernized diet, the macrophage‐specific ablation of *Atg5* (Razani *et al*, 2012) or the vascular smooth muscle cell‐specific deletion of *Atg7* (Osonoi *et al*, 2018) accelerates the acquisition of the atherogenic phenotype, linked to detrimental inflammasome activation or increased CCL2 (chemokine (C‐C motif) ligand 2)‐mediated macrophage recruitment, respectively. This result matches the original observation, indicating that undissolved cholesterol crystals instigate lysosomal damage and promote NLRP3 inflammasome activation (Duewell *et al*, 2010). In line with the atheroprotective role of autophagy, the stimulation of autophagy in macrophage foam cells limits plaque buildup by favoring cholesterol efflux. Mechanistically, autophagy promotes the delivery of lipid droplets (LDs) to the lysosome, where resident lysosomal acid lipases hydrolyze cholesterol esters to free cholesterol prior to the ABCA1 (ATP‐binding cassette, subfamily A (ABC1), member 1)‐dependent release (Ouimet *et al*, 2011). Moreover, it has recently been observed that an excess of dietary proteins is sufficient to drive the atherogenic phenotype in *apoe* and *ldlr* (low‐density lipoprotein receptor) knockout mice, due to the overactivation of MTORC1 signaling and the consequent inhibition of mitophagy in macrophages (Zhang *et al*, 2020). In advanced stages of atherosclerosis, autophagy contributes to maintain plaque integrity by promoting macrophage survival, as witnessed by the fact that *atg5* deletion in macrophages of *ldlr^−^* ^/^^−^ mice fed a HFD worsens the atherosclerotic phenotype due to exacerbated oxidative stress, impaired efferocytosis, and enhanced macrophage apoptosis (Liao *et al*, 2012). Corroborating this finding, stimulation of lysosomal biogenesis in macrophages by TFEB activation mitigates the atherogenic phenotype (Sergin *et al*, 2017). The atheropreventive functions of autophagy are not limited to macrophages. Indeed, defective endothelial autophagy in hypercholesterolemic mice dissipates the antiatherogenic effect of blood‐flow‐derived shear stress, worsening the burden of atherogenic plaques and exacerbating inflammatory reactions (Vion *et al*, 2017).

## Musculoskeletal disorders

The proper functioning of the musculoskeletal system depends upon the tightly coordinated integration of signals that operate to maintain an adequate balance between mass and structural requirements of the skeletal muscles, but also bone and cartilage. Of note, defects in the musculoskeletal system yield tangible systemic consequences, due to (i) the pivotal role of skeletal muscle in the systemic regulation of INS (insulin) signaling and (ii) the hormone‐mediated crosstalk between the renal and osseous systems for Ca^2+^ homeostasis.

### Muscular diseases

As briefly discussed above, intact autophagy is essential for the preservation of muscle structure and fitness at basal conditions (Sebastian & Zorzano, 2020) (Table 3). This observation is fully supported by experimental evidence revealing that autophagy‐incompetent muscle progressively degenerates as a direct consequence of aberrant proteostasis, leading to the development of severe myopathies (Masiero *et al*, 2009). Conversely, the stimulation of autophagy partially underlies the beneficial actions of physical exercise in maintaining muscle mass (He *et al*, 2012; Liu *et al*, 2020b), while retarding age‐dependent loss of muscle mass (sarcopenia) (Fan *et al*, 2016). In this regard, time‐dependent decline in autophagy proficiency has been functionally connected to accrued senescence of muscle satellite cells, suggesting that impaired autophagy is a key determinant of the sarcopenic phenotype (Garcia‐Prat *et al*, 2016). This tenet is further reinforced by recent observations demonstrating that suppression of the prostaglandin‐degrading enzyme HPGD/15‐PGDH (15‐hydroxyprostaglandin dehydrogenase) restrains sarcopenia progression through the activation of autophagy (Palla *et al*, 2021) and that the anti‐atrophy action of SESNs (sestrins) depends on autophagy activation (Segales *et al*, 2020). Noteworthy, impaired mitochondrial dynamics play a central role in age‐dependent muscle decay, with levels of most fusion genes falling during aging and other atrophy conditions (Hood *et al*, 2019), as witnessed by the fact that age‐dependent loss or genetic ablation of *Mfn2* in murine muscle precipitates sarcopenia via inhibition of mitophagy (Sebastian *et al*, 2016). However, the clinical relevance of mitochondrial dynamics in general in aging sarcopenia is unclear. In a cohort study, only levels of *OPA1* (OPA1 mitochondrial dynamin‐like GTPase), a gene essential for inner mitochondrial membrane fusion and cristae remodeling (Giacomello *et al*, 2020), correlate with muscle mass, and its inducible deletion in the adult mouse triggers FOXO3‐dependent sarcopenia and FGF21 (fibroblast growth factor 21)‐induced systemic aging (Tezze *et al*, 2017).

**Table 3:** Musculoskeletal disorders associated with genetic intervention of autophagy in mice.

Setting	Genetic intervention	Effects on disease phenotype	Ref.
Bone loss	Chondrocyte‐specific deletion of *Atg7*	Reduced femoral and tibia lengths, linked to increased ER storage of PC2 and defective secretion of COL2A1, at the postnatal stage	Cinque *et al* (2015)
Bone loss	Osteoblast‐specific deletion of *Atg5*	Reduced trabecular bone volume in 9‐month‐old mice, linked to reduced mineralization capacity	Nollet *et al* (2014)
Bone loss	Conditional osteoblast‐specific deletion of *Fip200*	Exacerbated osteopenia due to defective osteoblast terminal differentiation	Liu *et al* (2013a)
Bone loss	Conditional osteoblast progenitor‐specific deletion of *Atg7*	Reduced bone mass at both developmental and adult age, linked to reduced mineralization capacity and promoted ER stress	Li *et al* (2018)
Bone loss	Conditional osteocyte‐specific deletion of *Atg7*	Reduced bone mass in 6‐month‐old mice linked to increased ROS levels and reduced osteoclast number	Onal *et al* (2013)
Bone loss	Osteoclast‐specific deletion of *Atg5*	Increase trabecular bone volume and reduced ovariectomy‐induced bone loss	DeSelm *et al* (2011)
Bone loss	Myeloid cell‐specific deletion of *Atg7*	Reduced glucocorticoid‐ and ovariectomy‐induced osteoclast differentiation and bone loss	Lin *et al* (2016)
Exercise intolerance	Whole‐body allelic loss of *Becn1*	Decreased endurance and altered glucose metabolism during acute exercise, impaired exercise‐stimulated protection against HFD‐induced glucose intolerance	He *et al* (2012)
Exercise intolerance	Whole‐body knock‐in of mutant *Bcl2^AAA^*	Decreased endurance and altered glucose metabolism during acute exercise, impaired exercise‐stimulated protection against HFD‐induced glucose intolerance	He *et al* (2012)
Muscular dystrophy	Whole‐body deletion of *Col6a1*	Myopathic defects associated with impaired autophagic flux and aberrant organelle accumulation	Grumati *et al* (2010) Chrisam *et al* (2015)
Muscular dystrophy	Muscle‐specific knock‐in of *Akt*	Exacerbated muscular dystrophy after autophagy inhibition	Grumati *et al* (2010)
Muscular dystrophy	Whole‐body deletion of *Trim32*	Exacerbated muscular atrophy associated with impaired autophagic induction	Di Rienzo *et al* (2019)
Osteoarthritis	Articular cartilage‐specific deletion of *FoxO1*	Development of osteoarthritis‐like pathologies	Wang *et al* (2020a)
Osteoporosis	Whole‐body deletion of *Optn*	Early elevated osteoporotic bone loss, senescence of MSCs, and enhanced adipogenesis	Liu *et al* (2020c)
PDB	Whole‐body deletion of *Optn*	Bone lesions similar to PDB observed in patients, linked to increased osteoclastogenic potential and decreased type I IFN production	Wong *et al* (2020)
PDB	Whole‐body knock‐in of mutant *p62^P392L^*	Increased osteoclastogenic potential of bone microenvironment, but histologically normal bones	Hiruma *et al* (2008)
Sarcopenia	Muscle‐specific deletion of *Atg7*	Exacerbated muscle loss during denervation and fasting, and abolished sestrin‐mediated protection against disuse‐induced muscle atrophy	Masiero *et al* (2009), Segales *et al* (2020)
Sarcopenia	shRNA‐mediated muscle‐specific deletion of *15‐PGDH*	Increased aged muscle mass, strength, and exercise performance	Palla *et al* (2021)
Sarcopenia	Whole‐body deletion of *Sesn1*	Exacerbated disuse‐induced muscle atrophy after constitutive mTORC1‐signaling activation	Segales *et al* (2020)
Sarcopenia	Muscle‐specific deletion of *Mfn2*	Enhanced muscle atrophy and sarcopenia, linked to age‐induced mitochondrial dysfunction and ROS production, after mitophagy inhibition	Sebastian *et al* (2016)
Sarcopenia	Conditional muscle‐specific deletion of *Opa1*	Accelerated muscle atrophy linked to a precocious senescence phenotype and premature death	Tezze *et al* (2017)
Sarcopenia	Whole‐body deletion of *Trim32*	Exacerbated muscle atrophy associated with impaired autophagic flux	Di Rienzo *et al* (2019)
XLMTM	Whole‐body deletion of *Mtm1*	Myopathic defects associated with impaired autophagic flux and abnormal mitochondria	Fetalvero *et al* (2013)

HFD, high‐fat diet; MSC, mesenchymal stem cell; PC2, type II procollagen; PDB, Paget disease of bone; XLMTM, X‐linked myotubular myopathies.

In the light of these studies, whether autophagy ameliorates or exacerbates pathological settings of sarcopenia, remains controversial. Indeed, studies reported (i) pathological contexts in which deficient autophagy is pathognomonic to the disease; (ii) muscular illnesses in which supraphysiological levels of autophagy aggravate the degenerative phenotype; (iii) musculo‐degenerative conditions (e.g., lysosomal storage disorders) in which the lysosomal system is aberrantly altered (Vainshtein *et al*, 2014; Castets *et al*, 2016); and (iv) conditions in which pharmacological activation of muscular autophagy reinstalls functionality of the muscle (Chrisam *et al*, 2015).

In degenerative myopathies, such as collagen type VI‐related myopathies, failure in autophagy initiation is observed in the muscle of *col6a1* (collagen, type VI, alpha‐1)*‐*knockout mice, resulting in aberrant organelle accumulation, mainly due to reduced expression of BECN1 (Grumati *et al*, 2010). More recently, a pathological role has been ascribed to dysfunctional autophagy in (i) Duchenne muscular dystrophy, as autophagy induction is hampered in adult mice displaying muscular dystrophy (*Dmd^mdx^* mutant mice) (De Palma *et al*, 2014); and (ii) X‐linked myotubular myopathies, as defective autophagy is detected in *Mtm1* (X‐linked myotubular myopathy gene 1)*‐*deficient mouse muscle (Fetalvero *et al*, 2013). Limb‐girdle muscular dystrophy 2H (LGMD2H) is a muscle dystrophy caused by mutations in the ubiquitin ligase TRIM32, characterized by impaired muscle regrowth following atrophy (Kudryashova *et al*, 2012). Recently, it has been reported that *TRIM32*‐mutant muscle cells show a defective autophagy response to atrophic stimuli, associated with increased ROS and TRIM63/MuRF1 levels. The pro‐autophagy function of TRIM32 depends on its ability to bind to AMBRA1 (autophagy/Beclin 1 regulator 1) and ULK1 and stimulate ULK1 activity via unanchored K63‐linked polyubiquitin (Di Rienzo *et al*, 2019). In contrast with these findings, activated autophagy seems to accelerate the muscular dystrophic alterations observed in congenital myotonic dystrophy type I patients (Beffy *et al*, 2010). A large body of evidence supports the notion that impaired fusion of autophagosomes with lysosomes exerts detrimental effects at the muscular level. This tenet has been confirmed in Danon disease, X‐linked myopathy with excessive autophagy and Pompe disease mouse models, in which autophagosomes are aberrantly accumulated due to impaired lysosomal degradation (Lieberman *et al*, 2012). Of note, strategies based on the enhancement of cellular waste disposal capacity (i.e., TFEB‐TFE3 gene therapy) hold promise of preclinical benefits in these pathological scenarios (Spampanato *et al*, 2013; Bajaj *et al*, 2019).

### Bone disorders

Autophagy has a well‐recognized impact on the regulation of numerous aspects of bone biology, acting as a primary determinant of bone mass, structure, and functional remodeling (Shapiro *et al*, 2014; Yin *et al*, 2019) (Table 3). This is mainly due to the fact that autophagy is essential for the survival and landmark functions of osteoblasts and osteoclasts, which operate antagonistically to maintain a constant equilibrium between events of bone mineralization and bone resorption, respectively (Shapiro *et al*, 2014; Vrahnas *et al*, 2019; Yin *et al*, 2019). Furthermore, autophagy positively regulates chondrocyte functions, directly contributing to the secretion of COL2A1 (collagen, type II, alpha‐1; the major component of the cartilage matrix) in response to FGF18 at the postnatal stage (Cinque *et al*, 2015). Additionally, the autophagy pathway is directly modulated in response to hormonal and soluble signals (including bone morphogenetic proteins, TNFSF11/RANKL [tumor necrosis factor (ligand) superfamily, member 11], and CTNNB1/β‐catenin) that intercept the central signaling pathway involved in bone mineralization dynamics. Based on this premise, it is not surprising that conditions that directly or indirectly disturb these processes evoke conditions of osteopetrosis, osteopenia, or osteoporosis (Shapiro *et al*, 2014; Dallas *et al*, 2018; Yin *et al*, 2019).

In line with the involvement of autophagy in events of bone mineralization, apatite crystals are detected within autophagic vacuoles in osteoblasts *in vitro* prior to their secretion. Furthermore, osteoblast‐restricted *Atg5* ablation dampens their mineralization capacity, culminating in decreased trabecular bone mass (Nollet *et al*, 2014). In addition, several components of the ATG machinery support osteoclast secretory functions by promoting the polarized fusion of lysosomes with the plasma membrane. This phenomenon, which relies upon intact ATG5 and RAB7 expression, suggests that non‐canonical tasks of ATG proteins may contribute to osteoclast‐dependent bone resorption (DeSelm *et al*, 2011).

Moreover, deletion of *Rb1cc1* compromises the differentiation of osteoblasts into osteocytes, instigating episodes of osteopenia (Liu *et al*, 2013a). Likewise, *atg7* knockout in differentiated osteoblasts or osteoblast precursors in the bone marrow impairs mineralization, due to ramping ER stress in target cells (Li *et al*, 2018). Along similar lines, alterations in the activity of the transcription factor ATF4, which has been found mutated in two genetic diseases of the skeletal system (such as Coffin‐Lowry syndrome and neurofibromatosis type I), reduce the expression of key *Atg* genes and impair bone mineralization (Li *et al*, 2018). Aside from its role in osteoblasts, genetic inhibition of autophagy in terminally differentiated osteocytes, which primarily act as mechanosensors within the skeletal system, results in a significant bone mass reduction (Onal *et al*, 2013). A significant body of experimental evidence suggests that autophagy also affects bone resorptive capacity, by virtue of its involvement in the differentiation (which seems to rely on the HIF1A/HIF1α [hypoxia‐inducible factor 1, alpha subunit]‐BNIP3 axis, but is unaffected by *atg5* deletion) (Zhao *et al*, 2012) and activity of osteoclasts (Shapiro *et al*, 2014; Dallas *et al*, 2018; Yin *et al*, 2019). In this regard, genetic inhibition of several autophagy genes in osteoclasts undermines the chain of events that lead to the release of acidic lysosomes at the contact site between bony surface and podosomes, resulting in increased bone volume (DeSelm *et al*, 2011). In view of the myriad actions in the skeletal tissue, researchers have investigated the role of autophagy in the pathogenesis of osteoporosis, which represents a significant health concern, especially among the elderly or post‐menopausal women. A genome‐wide association study established a correlation between genetic variants in several ATG proteins and wrist bone mineral density, suggesting that altered autophagy may predispose to the osteoporotic phenotype (Zhang *et al*, 2010). Considering that osteoporosis is a multifactorial disorder, establishing an etiological connection between autophagy and the onset of the disease remains a challenging task. In a rat model of osteoporosis, reduced levels of autophagy in osteoblasts have been reported (Tang *et al*, 2019). *optn^−^* ^/^^−^ mice show reduced ability to eliminate FABP3 (fatty acid binding protein 3, muscle and heart) by selective autophagy linked to impaired osteogenesis and increased bone loss, thus supporting the notion that decreased expression of OPTN during aging might lead to osteoporosis (Liu *et al*, 2020c). In contrast, genetic inactivation of autophagy in myeloid cells prevents osteoclastogenesis, while mitigating bone loss in mice treated with glucocorticoids or subjected to ovariectomy (Lin *et al*, 2016). This result fits well with the observation that exacerbated inflammatory signals, typified by TNF/TNF‐α‐mediated activation of autophagy in osteoclasts, are detrimental for bone loss (Lin *et al*, 2013).

A possible connection has also been put forward between disturbance in autophagy and Paget disease of bone (PDB), an age‐dependent pathology defined by altered bone turnover due to aberrant osteoclast activity. Mutations in the gene coding for SQSTM1/p62 have been found in approximately 10% of PDB patients, and a mouse model carrying the P394L mutation exhibits a PDB‐like bone disorder with focal bone lesions, linked to enhanced autophagy activation in osteoclasts and detrimental bone remodeling (Hiruma *et al*, 2008). Recently, genetic ablation of *Optn* in mice has been found to recapitulate the clinical features observed in human PBD patients. Mechanistically, OPTN deficiency maps to defective IFNB1/IFNβ1 (interferon beta 1) production and signaling, in turn linked to enhanced osteoclast differentiation and survival (Wong *et al*, 2020). Furthermore, mutations in VCP cause early‐onset Paget disease in conjunction with frontotemporal dementia and inclusion body myositis. The hallmark pathology of familial or sporadic inclusion body myositis consists of a massive accumulation of autophagy vacuoles and polyubiquitinated aggregates large enough to be visualized by routine histology as rimmed vacuoles (Nogalska *et al*, 2010).

Finally, dampened levels of ATG proteins (including ULK1, LC3, and BECN1) have been described in a mouse model of osteoarthritis (OA), the most prevalent joint pathology (Carames *et al*, 2010). This result lends further ground to the evidence that autophagy regulates central functions in chondrocytes, even at the adult stage. In support of this result, the induction of autophagy mediated by FOXO1 is instrumental for the activation of TFGB signaling and protects against OA. Conversely, the postnatal ablation of *FoxO1* or its cartilage‐restricted suppression in adult mice is sufficient to drive an OA‐like symptomatology (Wang *et al*, 2020a). In this context, intact autophagy responses are instrumental to counteract the inflammatory burden that delineates OA pathogenesis, while concomitantly limiting IL1 (interleukin 1)‐induced erosion of cartilage matrix through efficiently dismantling inflammasomes and improving mitochondrial turnover (Sasaki *et al*, 2012; Kim *et al*, 2017). Because cellular senescence is functionally implicated in OA pathogenesis, it is plausible to speculate that defective autophagy contributes to OA by promoting chondrocyte senescence (Coryell *et al*, 2021).

## Pulmonary disorders

Functional autophagy responses are required to fulfill multiple homeostatic tasks within the variety of cell types that forms the pulmonary tissue, thus ensuring a functional gas exchange in the lung. Of note, autophagy elicits cytoprotective or disease‐supporting roles in the most common pathologies affecting the lung tissues (Table 4).

### Chronic obstructive pulmonary disease

Chronic obstructive pulmonary disease (COPD) is a progressively debilitating disease caused by chronic exposure to cigarette smoke (CS), currently representing the fourth leading cause of death worldwide. The pathogenic features of COPD encompass airway obstruction and loss of alveolar cells (called emphysema), which lead to an aberrant remodeling of the lung parenchyma and irreversible decline of lung function. Preclinical models of CS exposure have delineated the pathological relevance of autophagy in COPD development (Nakahira *et al*, 2016). Consistently, partial autophagy deficiency imposed by *map1lc3b* deletion reduces signs of emphysema after 3‐month exposure to CS (Chen *et al*, 2010). In similar experimental settings, *map1lc3b^−^* ^/^^−^ and *Becn1^+^* ^/^^−^ animals display enhanced resistance to CS‐induced mucociliary disruption, suggesting that autophagy‐dependent degradation of bronchial cilia (known as “ciliophagy”) elicits detrimental outcomes in COPD (Lam *et al*, 2013). Further corroborating the negative role of cilia resorption in COPD, genetic, or pharmacological inhibition of HDAC6 (histone deacetylase 6) with tubastatin A leads to decreased autophagy, followed by reduced cilia shortening and protection from CS‐induced lung dysfunction (Lam *et al*, 2013). In agreement with these results, *mir21^−^* ^/^^−^ mice exposed to CS exhibit improved pulmonary fitness compared with their wild‐type counterparts, alongside a reduction in markers of autophagy activation and decreased apoptosis of bronchiolar cells (Zeng *et al*, 2018). Recently, a possible correlation between selective lysosomal degradation of ferritin (known as “ferritinophagy”) and COPD has emerged, suggesting that NCOA4 (nuclear receptor coactivator 4)‐dependent ferritinophagy occurring upon CS exposure accelerates COPD progression by instigating parenchymal lung cell ferroptosis (Yoshida *et al*, 2019). Besides sensitizing parenchymal lung cells to death, the stimulation of autophagy by CS exposure precipitates neutrophil death, in turn resulting in the detrimental release of elastase in the lung. Mechanistically, this effect relies on the CS‐dependent activation of PAFR (platelet‐activating factor receptor), which in turn leads to autophagy upregulation in neutrophils (Lv *et al*, 2017).

In the recent past, a number of studies have investigated the contribution of mitophagy to COPD pathogenesis, leading to discordant findings (Cloonan & Choi, 2016). Defective mitophagy imposed on mice by *pink1* deletion or by treatment with the mitophagy inhibitor Mdivi‐1 protects lung epithelial cells from CS‐induced necroptotic cell death, while improving lung function (Mizumura *et al*, 2014). Nonetheless, the inhibition of mitophagy associated with the genetic deletion of *Prkn* worsen the effect of CS, as it promotes the entry of epithelial alveolar cells in the senescent state (Ahmad *et al*, 2015). Because senescence operates as a major pathogenic mechanism in COPD and settings of derailed autophagy facilitate the installation of the senescent program (Antony & Thannickal, 2018), it is tempting to speculate that prolonged suppression of autophagy and mitophagy may instead contribute to the clinical manifestation of COPD. Further studies, addressing autophagy/mitophagy incompetency in selected cell types within the lung tissues, and triggered by additional manipulations, will be instrumental to clarify this conundrum.

### Pulmonary fibrosis

Unlike COPD, autophagy appears to elicit protective functions in murine models of pulmonary fibrosis induced by chemotherapeutics (i.e., bleomycin) or silica (Patel *et al*, 2012; Zhao *et al*, 2019; Zhao *et al*, 2020). Of note, induction of lung injury produced by these agents leads to adverse inflammatory events, which may causally contribute to an excessive healing process and fibrogenesis (Racanelli *et al*, 2018). Although these preclinical systems present inherent limitations, because they fail to recapitulate key features of human interstitial lung disorders, they are currently employed to study the pathological underpinnings of idiopathic pulmonary fibrosis, sarcoidosis, and lung injury. Partial autophagy incompetency driven by type II alveolar epithelial cell‐specific knockdown of *Tsc1* or whole‐body *atg4b* knockout exacerbates bleomycin‐induced lung injury (Cabrera *et al*, 2015; Gui *et al*, 2015). Moreover, activation of MTORC1 in macrophages by selective deletion of *Tsc2* leads to excessive granuloma formation, a clinical implication for sarcoidosis (Linke *et al*, 2017). In addition, defective autophagy in progenitor alveolar type 2 (AT2) cells aggravates bleomycin‐induced lung injury, as it reduces AT2 cell stemness by reprogramming their metabolism (Li *et al*, 2020a). Consistently, bleomycin‐induced upregulation of ANXA2 (Annexin A2) perturbs the autophagic flux by limiting TFEB nuclear translocation (Wang *et al*, 2018a). Supporting these results, TLR4‐dependent activation of autophagy in a mouse model of silicosis is required to resolve chronic lung injury (Yang *et al*, 2012).

The antifibrotic properties attributed to autophagy in the context of acute or chronic lung injury are presumably tied to (i) enhanced resistance of alveolar epithelial cells to programmed death; (ii) reduced TGFB/TGFβ (transforming growth factor, beta)‐dependent fibroblast differentiation; and (iii) suppression of the inflammatory cascade (Patel *et al*, 2012; Mora *et al*, 2017; Zhao *et al*, 2019; Zhao *et al*, 2020). As an example, mice characterized by autophagy deficiency in myeloid cells display exacerbated inflammation and fibrosis compared with their autophagy‐competent littermates in the context of bleomycin‐ or silica‐induced fibrosis (Abdel Fattah *et al*, 2015; Jessop *et al*, 2016). Derailed mitochondrial fitness participates in the fibrogenic process in pulmonary fibrosis. In accordance with this notion, genetic loss of *Pink1* and *Prkn* accelerates the development of the fibrotic phenotype in bleomycin‐treated mice, linked to alveolar epithelial cell II (AECII) loss and accrued inflammation (Bueno *et al*, 2015; Kobayashi *et al*, 2016). Of note, the levels of PINK1 decline with age, suggesting that a time‐dependent drop in mitophagy proficiency may contribute to the development of pulmonary fibrosis in aged individuals (Bueno *et al*, 2015).

**Table 4:** Pulmonary disorders associated with genetic intervention of autophagy in mice.

Setting	Genetic intervention	Effects on disease phenotype	Ref.
COPD	Whole‐body deletion of *Map1* *lc3b*	Decreased signs of emphysema and resistance to cilia shortening after CS exposure	Chen *et al* (2010), Lam *et al* (2013)
COPD	Whole‐body allelic loss of *Becn1*	Resistance to cilia shortening after CS exposure	Lam *et al* (2013)
COPD	X chromosome deletion of *Hdac6*	Resistance to cilia shortening after CS exposure	Lam *et al* (2013)
COPD	Whole‐body deletion of *miR‐21*	Improved pulmonary fitness after CS exposure by reducing autophagy activation in bronchiolar cells	Zeng *et al* (2018)
COPD	Whole‐body deletion of *Pink1*	Improved lung function after subchronic CS exposure, linked to impaired mitophagy	Mizumura *et al* (2014)
Pulmonary fibrosis	Whole‐body deletion of *Atg4b*	Exacerbated bleomycin‐induced lung injury linked to increased lung inflammation	Cabrera *et al* (2015)
Pulmonary fibrosis	Conditional AEC‐specific deletion of *Tsc1*	Exacerbated bleomycin‐induced lung injury after mTORC1 overactivation	Gui *et al* (2015)
Pulmonary fibrosis	Conditional A2T progenitor cell‐specific deletion of *Atg5*	Exacerbated bleomycin‐induced lung injury by reducing A2T stemness	Li *et al* (2020a)
Pulmonary fibrosis	Whole‐body deletion of *Anxa2*	Mitigated bleomycin‐induced lung injury via TFEB‐mediated autophagy activation	Wang *et al* (2018a)
Pulmonary fibrosis	Whole‐body deletion of *Tlr4*	Exacerbated bleomycin‐induced lung injury and pulmonary inflammation after autophagy inhibition	Yang *et al* (2012)
Pulmonary fibrosis	Conditional myeloid cell‐specific deletion of *Atg5* or *Atg7*	Exacerbated bleomycin‐induced fibrosis and spontaneous lung inflammation by enhancing inflammasome activation	Abdel Fattah *et al* (2015), Jessop *et al* (2016)
Pulmonary fibrosis	Whole‐body deletion of *Pink1*	Accelerated development of bleomycin‐induced lung fibrosis linked to accumulation of dysfunctional mitochondria in AEC cells	Bueno *et al* (2015)
Pulmonary fibrosis	Whole‐body deletion of *Prkn*	Accelerated development of bleomycin‐induced lung fibrosis after mitophagy inhibition	Kobayashi *et al* (2016)
Sarcoidosis	Conditional myeloid cell‐specific deletion of *Tsc2*	Exacerbated granuloma formation after mTORC1‐mediated hypertrophy and proliferation in macrophages	Linke *et al* (2017)
Cystic fibrosis	CFTRdel506 transgenic mice	Impaired autophagy through TG2‐mediated BECN1 inhibition	Luciani *et al* (2010)

AEC, alveolar epithelial cell; A2T, alveolar type 2; COPD, chronic obstructive pulmonary disease, CS, cigarette smoke.

### Cystic fibrosis

Cystic fibrosis (CF) is a genetic autosomal recessive disease, due to mutations in the *CFTR* (cystic fibrosis transmembrane conductance regulator) gene, with the most frequent one being *CFTRdel506* (Rowntree & Harris, 2003). Loss‐of‐function mutations of *CFTR* lead to its reduced expression or affect its correct transport to the plasma membrane. The production of abnormally viscous mucus, associated with declining functions of lung epithelial cells and macrophages, renders CF patients susceptible to infections and aberrant inflammation, which eventually account for the fatal outcome of this disease. Of note, a large body of evidence indicates that CFTR defects impair autophagy, through mechanisms that include the sequestration of BECN1 (and its interactome) in aggresomes (Luciani *et al*, 2010; Luciani *et al*, 2011) and an impairment in xenophagy. Treatment of mice bearing the *Cftrdel506* mutation with a combination of EGCG (an inhibitor of the autophagy repressor EP300) and cysteamine (which restores the trafficking of CFTRdel506 to the membrane by inhibiting TGM2 [transglutaminase 2, C polypeptide]) yield to tangible clinical and preclinical benefits in autophagy‐competent mice, yet fail to do so in their autophagy‐deficient counterparts, further emphasizing the key involvement of autophagy in CF pathogenesis (Tosco *et al*, 2016). Mechanistically, it has been demonstrated that TGM2 triggers the trimerization and activation of HSF1 (heat‐shock transcription factor 1) regulating adaptation to stress and proteostasis impairment. TG2 loss of function correlates with a defect in the nuclear translocation of HSF1 and restores the imbalance in the HSF1‐HSPA/HSP70 pathway in CF leading to an increase in approximately 40% in CFTR function in a CF mouse model lacking TGM2 (Rossin *et al*, 2018). Interestingly, mice bearing defective CFTR are abnormally susceptible to a celiac disease‐like enteropathy as a consequence of inflammatory response induced by oral challenge with the gluten‐derivative gliadin (Villella *et al*, 2019b). Further, stimulation of autophagy by restored expression of BECN1 attenuates this gliadin‐induced inflammation (Villella *et al*, 2019a).

## Kidney diseases

Intact autophagic responses are essential to regulate baseline functions of resident kidney cells, while exerting renoprotective effects under conditions of acute or chronic damage (Choi, 2020; Tang *et al*, 2020) (Table 5). Unlike the conditional deletion of essential autophagic genes at the embryonic stage, which does not significantly have an impact on normal kidney development, the promoter‐specific invalidation of autophagy in adult mice severely affects kidney physiology, depending upon the targeted cell type. As an example, the *Six2* (sine oculis‐related homeobox 2) promoter‐driven expression of Cre recombinase in *Atg5^fl^* ^/^ *^fl^* or *Atg7^fl^* ^/^ *^fl^* mice, which renders the entire nephron incompetent for autophagy, is accompanied by the detrimental remodeling of tubular and glomerular structures and leads to irreversible renal failure (Kawakami *et al*, 2015). Likewise, *atg5* deletion in both distal and proximal tubular epithelial cells (TECs) results in progressive kidney damage and tubulointerstitial fibrosis (Liu *et al*, 2012). The same result is not observed in settings of autophagy deficiency in distal TECs only, suggesting that proximal TECs are more reliant upon basal autophagy than their distal counterparts (Liu *et al*, 2012). Importantly, disturbance of the autophagy flux in podocytes, by podocyte‐specific deletion of *Atg5* (Hartleben *et al*, 2010), *Pik3c3*/*Vps34* (Bechtel *et al*, 2013), or *Ctsd* (cathepsin D) (Yamamoto‐Nonaka *et al*, 2016), underpins events of glomerulosclerosis and proteinuria, culminating in severe glomerulopathy and kidney dysfunction. Of note, the phenotypic alterations associated with the suppression of autophagy within multiple components of the renal system become clinically manifest (or exhibit worsened features) with age, implying that defective autophagy is a primary driver of kidney aging (Tang *et al*, 2020). This result seems to corroborate the observations that the expression of the autophagy suppressor protein RUBCN increases over time, alongside exacerbated markers of defective lysosomal function (Matsuda *et al*, 2020).

### Acute kidney injury

The capacity of tubular cells to activate autophagy elicits protection against various forms of acute kidney injury, including IRI driven by kidney artery clamping, cisplatin treatment, oxalate crystals, and infectious agents (Kaushal & Shah, 2016; Choi, 2020; Nakamura *et al*, 2020; Tang *et al*, 2020). Regardless of the experimental setting, inactivation of autophagy in TECs exacerbates the noxious effects of IRI, sensitizing kidney‐resident cells to death (Kaushal & Shah, 2016; Choi, 2020; Tang *et al*, 2020). By contrast, uncontrolled activation of autophagy as mediated by *rubcn* deletion fails to elicit renoprotective effects against IRI, possibly indicating autophagy‐independent function of the protein or because of autosis induction (Matsuda *et al*, 2020). The maintenance of mitochondrial integrity is central to mount an adequate response to kidney IRI, as demonstrated by the observations that mitophagy is robustly activated in proximal TECs during IRI and that defective mitophagy imposed by *pink1* or *prkn* deletion aggravates kidney damage (Tang *et al*, 2018; Choi, 2020).

### Diabetic kidney disease

Diabetic kidney disease (DKD) represents one of the most common forms of chronic kidney pathologies. Dysfunctional autophagy plays a major contributing role in the pathogenesis of DKD. For example, streptozotocin‐induced chronic hyperglycemia leads to glomerulopathy, whose phenotypic manifestation is more severe in *Atg5*‐deficient podocytes than their wild‐type counterparts (Lenoir *et al*, 2015). In proximal TEC, an inverse correlation has been established between autophagy levels and the expression of SLC5A2/SGLT2 (solute carrier family 5 member 2), which mediates glucose reabsorption. Accordingly, *slc5a2* deletion reduces the pathological accumulation of SQSTM1/p62 in streptozotocin‐treated mice (Vallon *et al*, 2013). Supporting this notion, recent results indicate that autophagy is impaired in DKD through TP53‐*Mir214*‐dependent downregulation of ULK1 (Ma *et al*, 2020). Ablation of *Mir214* from proximal TEC or TP53 block rescues kidney hypertrophy and albuminuria, restoring autophagy (Ma *et al*, 2020). Furthermore, HDAC6‐mediated deacetylation of TFEB, which triggers transcriptional autophagy activation, improves the outcome of DKD in rats (Brijmohan *et al*, 2018). Along similar lines, OPTN‐dependent activation of mitophagy improves signs of diabetic nephropathy by counteracting premature senescence (Chen *et al*, 2018b) and reducing NRLP3 inflammasome activation (Chen *et al*, 2019), hence supporting the hypothesis that autophagy may exert beneficial effects via the suppression of inflammatory reactions. Along similar lines, OPTN‐dependent activation of mitophagy improves signs of diabetic nephropathy by counteracting premature senescence (Chen *et al*, 2018b) and reducing NRLP3 inflammasome activation (Chen *et al*, 2019), hence supporting the hypothesis that autophagy may exert beneficial effects via the suppression of inflammatory reactions.

**Table 5:** Kidney diseases associated with genetic intervention of autophagy in mice.

Setting	Genetic intervention	Effects on disease phenotype	Ref.
Acute kidney injury	Distal and proximal TEC‐specific deletion of *Atg5*	Impaired kidney function and increased sensitivity to ischemic injury, linked to accumulation of damaged mitochondria	Liu *et al* (2012)
Acute kidney injury	Proximal TEC‐specific deletion of *Atg5*	Exacerbated nephropathy induced by oxalate crystals	Nakamura *et al* (2020)
Acute kidney injury	Proximal TEC‐specific deletion of *Rubcn*	Increased sensitivity to ischemic injury, linked to increased fat efflux from cells to circulation, after autophagy activation	Matsuda *et al* (2020)
Acute kidney injury	Whole‐body deletion of *Pink1* and/or *Prkn*	Increased sensitivity to ischemic injury linked to damaged mitochondria, ROS production, and inflammatory response, after mitophagy inhibition	Tang *et al* (2018)
Acute kidney injury	Proximal TEC‐specific deletion of *Tfeb*	Enhanced progression of kidney injury induced by oxalate crystals, linked to exacerbation of lysosomal damage.	Nakamura *et al* (2020)
Diabetic kidney disease	Podocyte‐specific deletion of *Atg5*	Accelerated diabetes‐induced podocytopathy with a leaky GFB and glomerulosclerosis	Lenoir *et al* (2015)
Diabetic kidney disease	Proximal TEC‐specific deletion of *Atg7*	Exacerbated renal hypertrophy, tubular damage, fibrosis, inflammation, and albuminuria in diabetic mice	Lenoir *et al* (2015), Ma *et al* (2020)
Diabetic kidney disease	Whole‐body deletion of *Sglt2*	Reduced glomerular hyperfiltration, linked to decreased accumulation of SQSTM1 in streptozotocin‐treated mice	Vallon *et al* (2013)
Diabetic kidney disease	Proximal TEC‐specific deletion of *miR‐214* or *Tp53*	Reduced renal hypertrophy and albuminuria, by preventing autophagy impairment in diabetic kidneys	Ma *et al* (2020)
Focal segmental glomerulosclerosis	Nephron‐specific deletion of *Atg5* or *Atg7*	Development of kidney dysfunction by 2 months and organ failure by 6 months	Kawakami *et al* (2015)
Focal segmental glomerulosclerosis	Podocyte‐specific deletion of *Atg5*	Development of early glomerulopathy and proteinuria in aging mice, resulting in late‐onset glomerulosclerosis	Hartleben *et al* (2010)
Focal segmental glomerulosclerosis	Conditional podocyte‐specific deletion of *Vps34*	Premature death, development of early‐onset proteinuria and glomerulosclerosis	Bechtel *et al* (2013)
Focal segmental glomerulosclerosis	Podocyte‐specific deletion of *Ctsd*	Development of late‐onset glomerulosclerosis and proteinuria in aging mice	Yamamoto‐Nonaka *et al* (2016)
Kidney fibrosis	Proximal TEC (S3 segment)‐specific deletion of *Atg5*	Reduced tubular atrophy, senescence, and inflammation, linked to superior renal function 30 days after IRI	Baisantry *et al* (2016)
Kidney fibrosis	Conditional proximal TEC‐specific deletion of *Atg7*	Reduced tubular atrophy, nephron loss, and macrophages infiltration, during UUO‐induced fibrosis	Livingston *et al* (2016)
Kidney fibrosis	Whole‐body deletion of *Map1* *lc3b*	Exacerbated UUO‐induced fibrosis, linked to increased collagen deposition and TGF‐β production	Ding *et al* (2014)
Kidney fibrosis	Whole‐body allelic loss of *Becn1*	Exacerbated UUO‐induced fibrosis, linked to increased collagen deposition and TGF‐β production	Ding *et al* (2014)
Kidney fibrosis	Conditional distal TEC‐specific deletion of *Atg7*	Exacerbated UUO‐induced fibrosis, linked to accumulation of damaged mitochondria and TGF‐β production	Nam *et al* (2019)
Kidney fibrosis	Whole‐body deletion of *Pink1* or *Prkn*	Exacerbated UUO‐induced fibrosis, linked to impaired macrophage mitochondrial homeostasis	Bhatia *et al* (2019)
Kidney fibrosis	Myeloid cell‐specific deletion of *Mfn2*	Exaggerated kidney fibrosis after inhibition of macrophage mitophagy	Bhatia *et al* (2019)
Kidney fibrosis	Whole‐body *αKlotho* haploinsufficiency	Exacerbated renal fibrosis and accelerated CKD progression upon high phosphate diet following UNX	Shi *et al* (2016)
Kidney insufficiency	Conditional proximal TEC‐specific deletion of *Vps34*/*PI3KC3*	Impaired autophagy flux, causing a Fanconi‐like syndrome and renal insufficiency	Grieco *et al* (2018)
Proteinuria	Podocyte‐specific deletion of *Atg7*	Higher levels of proteinuria and ultrastructural changes following UNX	Oliva Trejo *et al* (2014)

CKD, chronic kidney disease; IRI, ischemia‐reperfusion injury; GFB, glomerular filtration barrier; ROS, reactive oxygen species; TEC, tubular epithelial cell; UNX, unilateral nephrectomy; UUO, unilateral ureteric obstruction.

### Polycystic kidney disease

Autosomal‐dominant polycystic kidney disease (ADPKD) is the most common genetic form of chronic renal disease. The appearance of the pathological phenotype is causally linked to mutations in the cilia‐regulating genes *PKD1* (polycystin 1, transient receptor potential channel interacting) or *PKD2*, coding for calcium channels (Choi, 2020), which have been linked to functional autophagy and maintenance of a physiological catabolic state (Pena‐Oyarzun *et al*, 2020). Cyst expansion observed in the ADPKD mouse model occurs along with an elevated MTOR activity, which is counteracted by treatment with rapamycin (Zafar *et al*, 2010; Choi, 2020). In keeping with this result, rapamycin treatment mitigates the pathological phenotype in a rat model of ADPKD when administered to male animals, yet fails to elicit renoprotective effects in female rats (Belibi *et al*, 2011). Interestingly, in a *pkd1* mutant zebrafish model of ADPKD, the genetic suppression of autophagy accelerates cystogenesis, whereas pharmacological stimulation of autophagy by BECN1‐activating peptide, rapamycin, or carbamazepine ameliorates kidney function (Zhu *et al*, 2017).

### Kidney fibrosis

In stark contrast with settings of acute kidney injury, the role of autophagy in the transition from acute to chronic kidney disease, which comes along with aberrant tissue repair and fibrosis, remains to be clarified. Because the recovery of kidney architecture entails a proliferative burst of resident kidney tubular cells, the suppression of autophagy responses after acute injury may be instrumental for regenerative repair (Li *et al*, 2014; Choi, 2020; Tang *et al*, 2020). Consistently, prolonged activation of autophagy during the reperfusion phase has been associated with events of autophagy‐dependent cell death and kidney fibrosis (Baisantry *et al*, 2016). Further corroborating the biphasic role of autophagy during IRI, whereas *atg5* deletion in TECs within the S3 segment predisposes proximal TECs to death, the inhibition of autophagy during the reperfusion phase instead facilitates the recovery of kidney function, accompanied by reduced markers of tubular cellular senescence (Baisantry *et al*, 2016). Hence, the pro‐fibrotic role of autophagy during the reperfusion phase seems to be tied to pro‐senescence actions of autophagy, possibly linked to the TOR‐autophagy spatial coupling compartment (TASCC)‐mediated production of pro‐fibrotic soluble mediators (Narita *et al*, 2011).

The contribution of autophagy to events of tubulointerstitial fibrosis has been extensively investigated in mouse models subjected to unilateral ureteral obstruction (UUO) or settings of TGFB administration/overexpression. The role of autophagy in the establishment of kidney fibrosis is controversial (Choi, 2020; Tang *et al*, 2020). Numerous reports validate the hypothesis that autophagy activation in UUO‐treated mice (Li *et al*, 2010; Livingston *et al*, 2016) or in murine models of TGFB overexpression in proximal TECs promotes fibrotic injury (Koesters *et al*, 2010). These results are supported by the observation that genetic or pharmacological inhibition of autophagy by chloroquine and 3‐methyladenine reduces the fibrotic burden in the kidney, suggesting that autophagy retains pro‐fibrotic effects in these pathological circumstances (Livingston *et al*, 2016; Tang *et al*, 2020).

By contrast, antifibrotic functions of autophagy have also been reported in mouse models of UUO‐induced fibrosis. Of note, *map1* *lc3b* deletion in proximal TECs leads to accrued COL1A (collagen, type I, alpha) production and severe fibrotic injury compared with autophagy‐competent animals (Ding *et al*, 2014). It is plausible to speculate that this effect could be associated with the anti‐inflammatory properties of autophagy, inasmuch as intact autophagy restrains NF‐κB (nuclear factor kappa B) signaling and NRLP3 inflammasome activation in UUO‐treated mice, thereby limiting noxious infiltration of inflammatory cells and decreasing fibrotic damage (Nam *et al*, 2019). Notably, dysfunctional mitophagy evoked by single or double *pink* and *prkn* knockout aggravates the fibrotic phenotype in UUO‐treated mice, by promoting macrophage reprogramming toward a pro‐fibrotic “M2‐like” phenotype (Bhatia *et al*, 2019). Maladaptive compensatory renal hypertrophy following surgical procedures, modeled in mice through unilateral nephrectomy (UNX), accelerates the transition from acute to chronic kidney injury, while enhancing the burden of tubulointerstitial fibrosis. Convergent evidence indicates that the autophagy flux is reduced during UNX (Brown *et al*, 2021). Concordant with this result, podocyte‐specific *Atg7*‐deficient mice display higher levels of proteinuria and ultrastructural changes following UNX (Oliva Trejo *et al*, 2014). In addition, KL/αKlotho‐haploinsufficient mice (which display reduced levels of autophagy) subjected to UNX plus contralateral ischemia‐reperfusion injury, exhibit elevated levels of fibrosis compared with their wild‐type counterparts. Conversely, restauration of autophagy flux mediated by KL overexpression or recombinant KL administration improves kidney functions after UNX (Shi *et al*, 2016).

## Metabolic syndromes

The ATG machinery has been evolutionarily devised to react to minimal oscillations in the intracellular and extracellular metabolic rheostat, with the purpose of maintaining a tightly regulated balance between anabolic and catabolic pathways (Rabinowitz & White, 2010; Galluzzi *et al*, 2014). In support of this tenet, essential molecular players of the cellular energetic state, such as MTORC1 and AMPK, are epistatic to autophagy initiation induced by nutritional changes (Jewell *et al*, 2013; Galluzzi *et al*, 2014). Because the lysosomal disposal of intracellular macromolecules invariably leads to their breakdown into essential metabolic intermediates, including amino acids, glucose, nucleotides, and free fatty acids (FAs), autophagy stands out as a key coordinator of the response to energetic stresses, at both the tissue‐specific and systemic levels (Rabinowitz & White, 2010; Galluzzi *et al*, 2014). Thus, autophagy fulfills tissue‐inherent metabolic tasks within the major organs involved in the maintenance of organismal energetic balance, including adipose tissue, liver, and exocrine pancreas (Kim & Lee, 2014; Lim *et al*, 2014). Additionally, intact autophagic responses directly interfere with the composition of the extracellular metabolome, thus contributing to the metabolic interconnectedness between different tissues that is essential in fine tuning an efficient response to bioenergetics cues (Galluzzi *et al*, 2014; Kim & Lee, 2014). In this context, autophagy exerts a crucial role in the adaptation to short‐ and long‐term metabolic stresses, while paving the way to compensatory systemic responses. For example, depletion of acetyl‐CoA promotes autophagy and blocks anabolic reactions, via activation of AMPK and consequent MTORC1 inhibition (Pietrocola *et al*, 2015). Consistently, the autophagy‐dependent release of DBI/ACBP/acyl‐CoA‐binding protein (diazepam binding inhibitor), which occurs upon starvation, leads to paracrine inhibition of autophagy in target cells accompanied by enhanced lipogenesis and food intake (Bravo‐San Pedro *et al*, 2019).

Circumstances of sustained energetic unbalance (encompassing excessive calorie assumption, dysregulated macronutrient intake, and reduced energy expenditure), mirrored by the aberrant activation of trophic axes (e.g., insulin signaling), contribute to the clinical manifestation of metabolic syndromes. These infirmities include type II diabetes (T2D), obesity and non‐alcoholic fatty liver disease (NAFLD), and their associated complications.

Commensurate with the multipronged layers of control over cellular bioenergetics, alterations in the autophagic flux affect the pathogenesis and progression of metabolic disorders (Ryter *et al*, 2014; Zhang *et al*, 2018; Menikdiwela *et al*, 2020) (Table 6). A large body of evidence supports the view that insufficient autophagy is pathognomonic to metabolic syndromes. In agreement with this notion, the genetic invalidation of several autophagy‐associated genes, including *Atg7* (Lim *et al*, 2014), *Atg4b* (Fernandez *et al*, 2017), *Becn2* (He *et al*, 2013), and *Tfeb* (Settembre *et al*, 2013), at the whole‐body level or in a tissue‐restricted manner, predisposes to the occurrence of metabolic disorders, both under a normal dietary regimen and obesogenic diets. Conversely, experimental settings of autophagy induction, for example, by ATG5 (Pyo *et al*, 2013) or TFEB overexpression (Settembre *et al*, 2013), or genetic or antibody‐mediated neutralization of DBI/ACBP (Bravo‐San Pedro *et al*, 2019), are sufficient to alleviate the metabolic anomalies tied to systemic energetic dysregulation and to mitigate characteristic signs of metabolic syndromes. Although these results support the hypothesis that autophagy‐stimulating therapies may lead to therapeutic advantages for the prevention and treatment of metabolic disorders, it is worth mentioning that autophagy inhibition in specific tissues (e.g., adipose tissue) may instead antagonize metabolic anomalies (Romero & Zorzano, 2019). Therefore, the overall phenotypic features that emerge from the systemic ablation of *Atg* genes are likely the net result of specialized functions of autophagy in metabolically relevant tissues. In this respect, the causal nexus between autophagy and metabolic syndrome can be explained by the multitiered actions of autophagy on (i) adipocyte differentiation (Singh *et al*, 2009b; Romero & Zorzano, 2019), (ii) accumulation of fat deposits in the liver, (iii) maintenance of pancreatic β‐cell fitness (Jung *et al*, 2008), (iv) central nervous system (CNS)‐mediated regulation of food intake (Kaushik *et al*, 2011), (v) inflammatory reactions (Zhong *et al*, 2016; Zhang *et al*, 2018), among other processes.

**Table 6:** Metabolic syndromes associated with genetic intervention of autophagy in mice.

Setting	Genetic intervention	Effects on disease phenotype	Ref.
Diabetes	Whole‐body allelic loss of *Atg7*	Development of obesity‐induced diabetes linked to augmented inflammation and lipid accumulation	Lim *et al* (2014)
Diabetes	Whole‐body deletion of *Atg4b*	Development of experimentally induced type I diabetes, linked to increased body weight gain upon HFD	Fernandez *et al* (2017)
Diabetes	Whole‐body knock‐in of mutant *Becn1^F121A^*	Improved insulin sensitivity, but impaired glucose tolerance upon HFD, after autophagy hyperactivation	Yamamoto *et al* (2018)
Diabetes	β cell‐specific deletion of *Atg7*	Reduced glucose tolerance due to reduced β‐cell mass, and development of obesity‐induced diabetes	Ebato *et al* (2008), Jung *et al* (2008), Quan *et al* (2012)
Diabetes	shRNA‐mediated liver‐specific deletion of *Atg7*	Reduced systemic glucose tolerance in obese mice linked to aberrant ER stress	Yang *et al* (2010)
NAFLD	shRNA‐mediated liver‐specific deletion of *Tfeb*	Increased development of severe ethanol‐induced liver injury, steatosis, and impaired lysosomal biogenesis	Chao *et al* (2018)
NAFLD	siRNA‐mediated liver‐specific deletion of *Atg7*	Increased ethanol‐induced hepatocyte apoptosis and liver injury	Ding *et al* (2010)
NAFLD	Hepatocyte‐specific deletion of *Rubcn*	Ameliorated liver steatosis and injury upon HFD, linked to activation of lipophagy	Tanaka *et al* (2016)
NAFLD	Myeloid cell‐specific deletion of *Atg5*	Enhanced toxin‐induced liver injury linked to production of pro‐inflammatory cytokines	Ilyas *et al* (2016)
NAFLD	Hepatocyte‐specific deletion of *Rb1cc1*	Increased endotoxin‐induced liver injury, inflammation, and hepatic fibrosis in FILKO mice	Ma *et al* (2013a)
NAFLD / Obesity	Hepatocyte‐specific deletion of *Tfeb*	Increased body weight gain upon HFD due to defects in lipid degradation	Settembre *et al* (2013)
NASH	Endothelial cell‐specific deletion of *Atg5*	Development of NASH and liver fibrosis, linked to enhanced inflammation	Hammoutene *et al* (2020)
Hepatic fibrosis	Hepatic stellate cell‐specific deletion of *Atg7*	Reduced experimentally induced fibrogenesis and matrix accumulation in the liver	Hernandez‐Gea *et al* (2012)
Hepatic steatosis	Hepatocyte‐specific deletion of *Atg7*	Marked increase in liver size, linked to increased lipid accumulation and impaired FA oxidation	Singh *et al* (2009a), Saito *et al* (2019)
Hepatic steatosis	Conditional hepatocyte‐specific deletion of *Lamp2*	Exacerbation of liver steatosis due to impaired lipophagy and FA oxidation, after CMA inhibition	Schneider *et al* (2014), Kaushik and Cuervo (2015a)
Hepatic steatosis	Whole‐body deletion of BNip3	Reduced β‐oxidation of fatty acids and impaired response to fasting. Elevated, inflammation, and steatohepatitis.	Glick *et al* (2012)
Hepatic steatosis	Hepatocyte‐specific deletion of *Vsp15*	Exacerbation of liver steatosis due to mitochondrial depletion and impaired FA oxidation	Iershov *et al* (2019)
Hepatic steatosis	Hepatocyte‐specific deletion of *Acox1*	Reduced hepatic steatosis caused by starvation or HFD after induction of autophagy	He *et al* (2020)
Metabolic syndrome	Whole‐body allelic loss of *Becn2*	Increased body weight gain upon HFD, impaired glucose tolerance, and decreased insulin sensitivity	He *et al* (2013)
Metabolic syndrome	Whole‐body overexpression of *Atg5*	Anti‐aging phenotypes, including leanness and increased insulin sensitivity	Pyo *et al* (2013)
Metabolic syndrome	Conditional whole‐body deletion of *Acbp*	Increase ability to maintain glucose levels in the normoglycemic range, by inducing lipid catabolism	Bravo‐San Pedro *et al* (2019)
Obesity	AgRP neurons‐specific deletion of *Atg7*	Reduced food intake, body weight, total fat, and WAT mass	Kaushik *et al* (2011)
Obesity	Adipocyte‐specific deletion of *Atg7*	Reduced body weight and WAT mass linked to enhanced insulin sensitivity and features of brown adipocytes	Singh *et al* (2009b), Zhang *et al* (2009)
Obesity	Adipocyte‐specific deletion of *Atg5* or *Atg12*	Reduced adipogenesis and body weight gain upon HFD, linked to enhanced insulin sensitivity and maintenance of beige adipocyte	Baerga *et al* (2009), Altshuler‐Keylin *et al* (2016)
Obesity	Whole‐body deletion of *Prkn*	Reduced maintenance of beige adipocyte due to mitophagy inhibition	Lu *et al* (2018)
Obesity	Conditional adipocyte‐specific deletion of *Atg3* or *Atg16L1*	Reduced adipose and systemic insulin resistance, linked to dysfunctional mitochondria and increased adipose tissue inflammation	Cai *et al* (2018)
Obesity	Adipocyte‐specific deletion of *Rubcn*	Increased systemic fat atrophy and hepatic lipid accumulation, after induction of excessive autophagy	Yamamuro *et al* (2020)

AgRP, agouti‐related peptide; CMA, chaperone‐mediated autophagy; FA, fatty acid; HFD, high‐fat diet; NAFLD, non‐alcoholic fatty liver disease; NASH, non‐alcoholic steatohepatitis; WAT, white adipose tissue.

### Obesity

Convergent evidence supports the hypothesis that autophagy also co‐regulates the program of adipogenesis in white adipose tissue (WAT). Accordingly, adipocyte‐restricted knockout of *Atg5* (Baerga *et al*, 2009) or *Atg7* (Singh *et al*, 2009b; Zhang *et al*, 2009) correlates with decreased expression of adipogenic factors, significant reduction in fat mass and increased UCP1 (uncoupling protein 1 [mitochondrial, proton carrier])‐dependent thermogenic capacity, commonly known as “browning”, which systemically map to a lean phenotype and heightened insulin sensitivity (Cairo & Villarroya, 2020). The anti‐obesogenic effect observed upon experimental settings of autophagy inhibition appears to be linked to the overaccumulation of mitochondria in WAT due to the impairment in mitophagy (Wrighton, 2016). Owing to its capacity to dispose of aged or damaged mitochondria, autophagy favors the plastic transition of “beige” adipocytes (i.e., brown‐like adipocytes within WAT deposits) toward a “white” phenotype (Cairo & Villarroya, 2020). Therefore, the UCP1‐specific deletion of *Atg5* or *Atg12* compromises the “beige‐to‐white” conversion under β‐adrenergic stimuli withdrawal, enabling mice to better cope with conditions of diet‐induced obesity and insulin resistance (Altshuler‐Keylin *et al*, 2016). Supporting the pro‐whitening function of mitophagy, the systemic inactivation of the mitophagy regulator PRKN promotes the maintenance of the beige phenotype through a mechanism that involves the β‐3 adrenergic‐mediated stimulation of PRKA (protein kinase, cAMP dependent), independently of UCP1 (Lu *et al*, 2018). Consistently, downregulation of the transcriptional program of lysosomal biogenesis orchestrated by the transcription factor family MITF (melanogenesis‐associated transcription factor)‐TFE prevents beige‐to‐white adipocyte transition leading to higher thermogenic capacity and protection against diet‐induced obesity and insulin resistance (Altshuler‐Keylin *et al*, 2016). While the transient inactivation of autophagy in adipocytes is instrumental to foster the systemic response to nutritional dysregulation, prolonged autophagy inhibition may nonetheless precipitate the obese phenotype, ultimately leading to defective differentiation, proteotoxic stress, and accrued inflammation (Cai *et al*, 2018; Zhang *et al*, 2018). Indeed, a systemic partial autophagy defect, as observed in *Atg4b*‐deficient mice, predisposes to diet‐induced obesity (Fernandez *et al*, 2017), and obesity is associated with increased plasma levels of autophagy‐inhibitory factors including DBI/ACBP, both in humans and in mice (Bravo‐San Pedro *et al*, 2019; Joseph *et al*, 2020). Adding to the complexity, the overactivation of autophagy through adipocyte‐specific knockout of *Rubcn*, a negative regulator of autophagy, markedly impairs the systemic metabolic balance by promoting adipose tissue atrophy and detrimental pile‐up of fat deposits in the liver (Yamamuro *et al*, 2020).

### Non‐alcoholic fatty liver disease

In the liver, autophagy takes active part in the orchestration of the metabolic response to opposite instances of metabolic stress, because it gets activated under both conditions of nutrient excess and scarcity (Ueno & Komatsu, 2017; Allaire *et al*, 2019; Hazari *et al*, 2020; Springer *et al*, 2021). Under conditions of nutritional overload, the acute induction of autophagy appears to primarily serve (i) to counteract the lipotoxic effect of free FAs, in particular those linked to dietary intake of saturated and trans‐unsaturated FAs, thus preserving the proteostatic and mitochondrial fitness of hepatocytes (Niso‐Santano *et al*, 2015; Madrigal‐Matute & Cuervo, 2016; Nguyen & Olzmann, 2017; Hazari *et al*, 2020); (ii) to prevent the aberrant expansion of triglyceride‐containing LDs by promoting their selective breakdown in the lysosome (Singh *et al*, 2009a; Singh & Cuervo, 2012); (iii) to reduce the acute toxicity associated with elevated alcohol consumption (Ding *et al*, 2010; Chao *et al*, 2018); and (iv) to counteract excessive lipid accumulation in hepatitis C virus‐infected hepatocytes (Vescovo *et al*, 2012). *De facto*, sustained nutritional imbalance over time and aberrant activation of the insulin signaling route abrogates the autophagic flux in the liver, leading to the onset of NAFLD, whose clinical manifestations span from non‐alcoholic steatosis to fibrosing non‐alcoholic steatohepatitis (NASH) (Allaire *et al*, 2019). Dampened levels of ATG proteins have been described in the liver of NASH patients or animals fed a methionine‐choline‐deficient diet (Allaire *et al*, 2019). In line with this result, the levels of the negative autophagy regulator RUBCN and SQSTM1/p62 are found increased in these pathological contexts (Tanaka *et al*, 2016).

The genetic inhibition of autophagy in the parenchymal (Settembre *et al*, 2013), stromal (e.g., endothelial cells) (Hammoutene *et al*, 2020), and immune (Ilyas *et al*, 2016) compartment of the liver sensitizes mice to the development of NAFLD via both cell autonomous (Yang *et al*, 2010) and non‐cell autonomous effects, linked to aberrant inflammatory reactions (Aghajan *et al*, 2012). Similarly, excessive generation of hepatic acetyl‐CoA in the liver via peroxisomal β‐oxidation inhibits autophagy, while accelerating the manifestation of hepatic steatosis (He *et al*, 2020).

Conversely, genetic interventions that enhance the autophagic flux (such as the increased expression of *Tfeb*) mitigate the induction of NAFLD favored by HFD regimens through activation of PPARGC1A/PGC‐1α (peroxisome proliferative activated receptor, gamma, coactivator 1 alpha) and PPARA/PPARα (peroxisome proliferator‐activated receptor alpha) transcriptional programs (Settembre *et al*, 2013) and/or through activation of lipophagy (Tanaka *et al*, 2016). In spite of these experimental lines of evidence, controversy still exist about the role of selective ATG proteins in NAFLD pathogenesis. As an example, the hepatocyte‐restricted deletion of *Rb1cc1* reduces triglyceride accumulation in NAFLD mouse models (Ma *et al*, 2013a).

Whereas autophagy downregulation generally predisposes to the development of NAFLD, such downregulation appears to limit fibrogenic responses in the liver. In this respect, a proficient autophagy flux is required for the transdifferentiation of hepatic stellate cells into extracellular matrix‐producing myofibroblasts, as illustrated by the fact that hepatic stellate cell‐specific ablation of *Atg5* protects mice from hepatic fibrosis induced by carbon tetrachloride (Thoen *et al*, 2011; Hernandez‐Gea *et al*, 2012).

In response to nutrient deprivation, BNIP3‐dependent mitophagy also plays a critical role in GCG (glucagon)‐induced metabolic responses of the liver (Springer *et al*, 2021). Zonal expression of BNIP3 and zonal patterning of mitophagy in liver parenchyma in response to nutrient deprivation contributes to zonal metabolic compartmentalization in the liver, and BNIP3 loss causes increased mitochondrial mass and disruption of urea cycle and glutamate–glutamine metabolism in particular (Springer *et al*, 2021).

Under nutrient shortage, hepatic autophagy maintains the organismal energetic balance through its crucial action of energy mobilization from nutrient stores, by hydrolyzing glycogen granules (a process known as “glycophagy”) and LDs in the lysosome. Whereas glycophagy defines the early phases after nutrient shortage, lipophagy operates (along with cytosolic lipases) as a crucial mechanism of resistance to sustained fasting (Singh & Cuervo, 2012; Madrigal‐Matute & Cuervo, 2016). Of note, the CMA‐mediated removal of PLINs (perilipins; which cover LDs) is epistatic to the initiation of lipophagy (Kaushik & Cuervo, 2015a) and may explain the upregulation of this type of autophagy early after a lipid challenge (Rodriguez‐Navarro *et al*, 2012). Consistently, the liver‐specific deficiency of CMA precipitates hepatic steatosis (Schneider *et al*, 2014), and the suppression of hepatic autophagy correlates with defective ketogenesis linked to the accumulation of the autophagy substrate NCOR1 (nuclear receptor co‐repressor 1), which suppresses the PPARA‐dependent transcriptional program of free FA oxidation (Iershov *et al*, 2019; Saito *et al*, 2019).

### Type 2 diabetes

Type 2 diabetes (T2D) clinically manifests with the appearance of insulin resistance in insulin‐responsive target cells, progressively accompanied by compromised function of insulin‐producing pancreatic β cells in Langerhans islets. Notably, autophagy appears to be etiologically implicated in both aspects of T2D pathogenesis. Defective autophagy in insulin‐responsive tissues (e.g., liver) fails to counteract the exacerbated levels of oxidative stress and ER stress upon persistent stimulation of the insulin‐signaling axis (Yang *et al*, 2010; Yamamoto *et al*, 2018; Zhang *et al*, 2018; Pietrocola & Bravo‐San Pedro, 2021). Autophagy also operates as a pivotal process in the regulation of pancreatic β cell homeostatic functions (Ebato *et al*, 2008; Jung *et al*, 2008). Under basal conditions, a selective form of autophagy (known as “crinophagy”) dedicated to the degradation of insulin‐containing granules contributes to regulate physiological levels of insulin in β cells (Lee *et al*, 2019). Unlike in the majority of cell types, short‐term starvation inhibits autophagy in pancreatic β cells through mechanisms of starvation‐induced nascent granule degradation (Goginashvili *et al*, 2015) and Golgi membrane‐associated degradation (Yamaguchi *et al*, 2016), thus serving as a buffer against the production of insulin in nutrient‐depleted conditions. Interestingly, the cell surface pyruvate transporter SLC16A11 is associated with risk of T2D (Rusu *et al*, 2017), and regulates autophagy (Velentzas *et al*, 2018).

A prominent surge in autophagy is detected in pancreatic β cells under conditions of nutritional challenges (e.g., HFD) or genetic LEP (leptin) deficiency. Such an increase in autophagy is required for the compensatory increase in β cell mass and survival of insulin‐producing cells, as witnessed by the fact that genetic ablation of *Atg7* in β cells promotes their demise, leading to impaired insulin production and glucose intolerance (Ebato *et al*, 2008). Mechanistically, defective autophagy maps to the incapacity of β cells to mount an adequate unfolded protein response/UPR, which is instrumental to sustain the hypersecretory phenotype of insulin‐producing β cells (Quan *et al*, 2012). Additionally, proficient autophagic response may contribute to the anti‐oxidative program elicited by NFE2L2/NRF2 activation in β cells, thus enabling them to withstand accrued oxidative burden associated with HFD (Abebe *et al*, 2017). In agreement with the concept that autophagy is essential for β‐cell survival, the interaction between C3 (complement component 3) and ATG16L1 underlies the maintenance of a functional autophagic flux during T2D, limiting the deleterious effects of nutritional stress on pancreatic β cells (King *et al*, 2019). Along similar lines, functional autophagy allows pancreatic β cells to sustain the detrimental proteotoxic stress linked to the intracellular accumulation and aggregation of IAPP (islet amyloid polypeptide), which is co‐secreted with insulin (Shigihara *et al*, 2014; King *et al*, 2019). While these experimental lines of evidence emphasize the positive role of autophagy in the regulation of β‐cell homeostasis, it is worth mentioning that constitutive activation of autophagy, by the expression of the knock‐in *Becn1^F121A^* dominant mutant, produces the paradoxical outcomes in the context of diet‐induced T2D of reducing glucose tolerance (due to the uncontrolled degradation of insulin granules) yet improving the responsiveness to insulin in peripheral tissues (Yamamoto *et al*, 2018). Future investigation is warranted to clarify this unexpected duality and to assess the clinical impact of autophagy‐inducing interventions in the prevention and management of metabolic syndromes.

## Other liver pathologies

Autophagy mediates widespread actions of control over the activity of the parenchymal and stromal components of the liver. Therefore, alterations in the autophagy flux are sufficient to instigate or modify hepatic pathological phenotypes (Hazari *et al*, 2020) (Table 7). As a consequence, the pharmacological targeting of autophagy is progressively emerging as a valuable translational approach for the prevention or treatment of hepatic disorders (Allaire *et al*, 2019).

**Table 7:** Other liver pathologies associated with genetic intervention of autophagy in mice

Setting	Genetic intervention	Effects on disease phenotype	Ref.
AATD	Liver‐specific knock‐in of human *Tfeb*	Reduced liver apoptosis and fibrosis, lined to promoted clearance of hepatotoxic ATZ in PiZ mice after autophagy activation	Pastore *et al* (2013)
Acute liver failure	Conditional liver‐specific deletion of *Atg7*	Development of hepatomegaly and hepatic cell swelling, and enhanced APAP‐induced liver injury	Komatsu *et al* (2005), Igusa *et al* (2012)
Acute liver failure	Liver‐specific deletion of *Atg5*	Development of hepatomegaly and basal liver injury, but resistance to APAP‐induced liver injury due to compensatory Nrf2 activation	Ni *et al* (2012b)
Acute liver failure	Conditional whole‐body deletion of *Atg5*	Development of hepatomegaly and hepatic cell swelling	Cassidy *et al* (2018)
Acute liver failure	Liver‐specific co‐deletion of *Ulk1* and *Ulk2*	Resistance to APAP‐induced liver injury independently of the autophagic process	Ni *et al* (2012b)
Cirrhosis	Myeloid cell‐specific deletion of *Atg5*	Exacerbated CCl_4_‐induced liver fibrosis linked to enhanced inflammatory infiltrate	Lodder *et al* (2015), Habib *et al* (2019)
Cirrhosis	Myeloid cell‐specific deletion of *Rubcn*	Exacerbated CCl_4_‐induced liver fibrosis linked to enhanced inflammatory infiltrate	Wan *et al* (2020)
Hyperammonemia	HDAd‐mediated liver‐specific deletion of *Atg7*	Higher levels of serum ammonia after ammonium chloride challenge	Soria *et al* (2018)

AATD, Alpha‐1 antitrypsin deficiency; APAP, acetaminophen; ATZ, alpha‐1‐antitrypsin; HDAd, helper‐dependent adenoviral.

### Cirrhosis

Cirrhosis is a late‐stage liver disease and a major health problem worldwide, in which liver tissue is permanently replaced by scar tissue, known as “fibrosis”, starting as a pathological consequence of chronic liver injury (such as hepatitis or alcoholic liver disease). Advances in the understanding of liver fibrosis have identified (i) sustained inflammation originating from macrophages as a driving force in the fibrogenic process (Krenkel & Tacke, 2017) and (ii) autophagy as a limiting factor to a pro‐inflammatory phenotype in macrophages. In particular, *atg5* deletion (Lodder *et al*, 2015; Habib *et al*, 2019) and genetic inhibition of LAP components (Wan *et al*, 2020) in the myeloid compartment exacerbate hepatic inflammation in mice with chronic liver injury, thus enhancing liver fibrosis. Accordingly, pharmacological blockade of LAP increases the inflammatory signature in human monocytes from patients with cirrhosis (Wan *et al*, 2020). These data are in line with the reported role of autophagy in limiting the pro‐fibrotic effects of macrophages in models of kidney (Bhatia *et al*, 2019) and lung fibrosis (Abdel Fattah *et al*, 2015; Jessop *et al*, 2016), thus suggesting that canonical and non‐canonical forms of autophagy prevent the reprogramming of macrophages to a pro‐inflammatory phenotype during events of fibrosis.

### Acute liver failure

The genetic suppression of basal autophagy in hepatocytes leads to hepatomegaly and exacerbated liver injury (Komatsu *et al*, 2005; Ni *et al*, 2012b; Cassidy *et al*, 2018). In addition, the induction of autophagy is required to counteract the aberrant levels of oxidative stress induced by acetaminophen (APAP) overdose, thus preventing APAP‐mediated necrotic death (Ni *et al*, 2012a). Conversely, genetic removal of *Atg7* precipitates the demise of hepatocytes exposed to a high APAP dose (Igusa *et al*, 2012). In contrast with these findings, the hepatocyte‐restricted deletion of *Atg5* protects liver parenchymal cells from APAP‐induced toxicity, casting the hepatoprotective role of autophagy in APAP‐induced toxicity into doubt (Ni *et al*, 2012b). Adding to the complexity, autophagy‐independent functions of ULK1/2 kinases (which mediate activation of MAPK8/c‐Jun N‐terminal kinase) appear to support the damaging actions of APAP in the liver (Sun *et al*, 2018; Allaire *et al*, 2019). Hence, it is tempting to speculate that gene‐dependent effects dictate the role of autophagy in this pathological context. Likewise, the role of autophagy in ischemia‐reperfusion hepatic injury remains controversial. Whereas autophagy seems to prevent liver injury shortly after ischemia‐reperfusion, the positive or negative contribution of autophagy during the reperfusion phase largely varies depending upon the experimental setting of ischemia (e.g., warm vs. cold) adopted (Gracia‐Sancho & Guixe‐Muntet, 2018).

### Genetic liver disorders

Wilson disease (WD) is a genetically inherited condition characterized by the toxic accumulation of copper in hepatocytes, which lead to hepatocyte poisoning and death, and eventually culminates in liver failure. The pathological phenotype emerges as a consequence of loss‐of‐function mutations in the gene coding for the intracellular copper export transporter ATP7B. Copper overload perturbs mitochondrion structure and dynamics, leading to the detrimental accumulation of non‐disposable mitochondria within the cell (Zischka & Einer, 2018). A compensatory/cytoprotective surge in the autophagy flux occurs in the liver of WD patients and in ATP7B‐deficient animals (Polishchuk *et al*, 2019). Consistent with this result, the genetic obliteration of *Atg7* (or the pharmacological inhibition of autophagy by spautin‐1) in copper‐challenged hepatocytes precipitates their death, supporting the view that autophagy is required to promote hepatocyte survival in WD (Polishchuk *et al*, 2019). Intriguingly, treatment of mice with the copper chelator triethylenetetramine promotes the activation of autophagy in the liver, further reinforcing the idea that autophagy activation may improve liver phenotype in WD patients (Pietrocola *et al*, 2020).

Alpha‐1 antitrypsin deficiency (AATD) is caused by loss‐of‐function mutations in SERPINA1/alpha‐1 antitrypsin mutant Z protein (ATZ), which compromises the ability of ATZ to properly fold and leads to its accumulation in the ER of hepatocytes. The toxic effect of ATZ inclusions pathologically manifests as liver injury, progressively leading to fibrosing liver disease (Allaire *et al*, 2019). The compensatory increase in autophagy is insufficient to reduce the pathological accumulation of ATZ inclusions, whereas the genetic ablation of *Atg5* precipitates hepatocyte death (Kamimoto *et al*, 2006). In this scenario, the increase in lysosomal biogenesis imposed on hepatocytes by *Tfeb* gene transfer in mice (Pastore *et al*, 2013), or the pharmacological activation of autophagy by carbamazepine or rapamycin, reduces the burden of fibrotic lesions in AATD mouse liver (Allaire *et al*, 2019).

### Hyperammonemia

Hepatic urea biosynthesis is required to minimize the neurotoxic effects associated with excessive accumulation of nitrogen waste in the blood. In a mouse model of acute hyperammonemia induced by ammonium chloride administration, autophagy is required for ammonia detoxification (Soria *et al*, 2018). Mechanistically, autophagy promotes hepatic ureagenesis and ammonia clearance by providing key urea cycle intermediates. In keeping with this result, pharmacological stimulation of autophagy by rapamycin, Tat‐Beclin 1 peptide, or *Tfeb*‐hepatic gene transfer improves the fitness of ammonium chloride‐challenged animals. In line with these data, Tat‐beclin 1‐mediated activation of autophagy improves the hepatic phenotype in two distinct urea cycle disorder mouse models (Soria *et al*, 2021).

### Cholestasis

The detrimental accumulation of bile acids is associated with severe hepatic damage and systemic clinical sequalae. Reduced bile acid flow compromises autophagy in patients with cholestasis. Mechanistically, bile acid overload impairs autophagosome‐to‐lysosome fusion depending upon the activation of NR1H4/farnesoid X receptor (nuclear receptor subfamily 1 group H member 4), which in turn controls the expression of the negative autophagy regulator RUBCN. In support of this result, the genetic ablation of *RUBCN* corrects bile acid‐mediated impairment of autophagy in an *in vitro* model of cholestasis (Panzitt *et al*, 2020).

## Cancer

Autophagy operates at the homeostatic forefront to preserve the genomic integrity of quiescent and proliferating cells in tissues (Hewitt & Korolchuk, 2017). From a mere cell intrinsic standpoint, autophagy generally prevents the neoplastic transformation of healthy cells (Galluzzi *et al*, 2015b). In support of this notion, pharmacological or genetic interventions hampering autophagic flux result in the appearance of early neoplastic lesions in a variety or preclinical tumor models (Galluzzi *et al*, 2015b). Thus, it is likely that autophagy in healthy cells operates as a tumor suppressor mechanism to counteract the effects of pro‐oncogenic stimuli (Rybstein *et al*, 2018). Supporting this concept, the activation of autophagy appears to be an essential step for the activation of the oncogene‐induced senescence program (Young *et al*, 2009). However, this reductionist standpoint needs to be framed within a more complex scenario, in which the actual contribution of autophagy to the biology of cancer depends on several aspects, including tumor type, disease stage, and host factors (Santana‐Codina *et al*, 2017). Indeed, proficient autophagy fosters the metabolic fitness of neoplastic cells, endowing them with the ability to cope with dwindling levels of energetic supply within the tumor bed (White, 2015; Kimmelman & White, 2017; Mukhopadhyay *et al*, 2021). Variations in the magnitude of the autophagy flux have been reported in the context of tumor metastatic recurrence, although the final outcome of autophagy modulation in these conditions strongly varies depending upon the type of cancer and the *Atg* object of investigation (Dower *et al*, 2018; Vera‐Ramirez *et al*, 2018; Marsh *et al*, 2020). In addition, autophagy is thought to participate in events of tumor relapse and resistance to therapy (Huang *et al*, 2020; Mele *et al*, 2020), in light of its direct involvement in the maintenance of a functional pool of cancer stem cells (Nazio *et al*, 2019; Smith & Macleod, 2019). Adding a further layer of complexity, autophagy in non‐transformed cells in the tumor microenvironment (TME; including stromal cells and resident or infiltrating leukocytes) plays a critical role in supporting cancer growth (Sousa *et al*, 2016; Katheder *et al*, 2017; Poillet‐Perez *et al*, 2018; Yang *et al*, 2018; Amaravadi *et al*, 2019). Moreover, perturbations in autophagy in immune cells that infiltrate the tumor niche also affect cancer dynamics in a highly context‐dependent manner, evoking immunostimulatory or immunosuppressive effects depending upon leukocyte subtypes involved, tumor stage, and therapeutic regimen (Amaravadi *et al*, 2019; Xia *et al*, 2021; Yamazaki *et al*, 2021). The development of mouse models in which genes encoding molecules involved in the autophagy machinery are deleted, and the mice are challenged with established protocols of chemical carcinogenesis or they are crossed with genetically engineered mouse models (GEMMs) of oncogene‐driven cancers, has enabled investigators to delve into the pathophysiological functions of autophagy in oncogenesis, tumor progression, and response to anticancer therapy (Galluzzi *et al*, 2015b; Amaravadi *et al*, 2016; Santana‐Codina *et al*, 2017) (Table 8). Because whole‐body knockout of essential *Atg* genes leads to perinatal lethality (Kuma *et al*, 2004; Komatsu *et al*, 2005), whole‐body knockout strategies to study the role of autophagy in cancer are limited to heterozygous deletion models such as *Becn1^+^* ^/^^−^, which achieves only partial autophagy incompetence. In order to achieve complete autophagy suppression, conditional knockout mice and inducible conditional knockout mice have been used. As an important disclaimer, the vast majority of these studies is based on the deletion of *Atg* genes that are functionally implicated in the regulation of pathways other than autophagy (e.g., LAP) (Xia *et al*, 2021), opening the possibility that alternative mechanisms would underlie the tumor‐modulating properties of the autophagy pathway.

**Table 8:** Malignancies associated with genetic intervention of autophagy in mice.

Setting	Genetic intervention	Effects on disease phenotype	Ref.
Bladder cancer	Conditional whole‐body deletion of *Atg7* or *Atg5*	Impaired growth of allografted MB49 urothelial cancer cells, linked to reduced circulating arginine, and increased antitumor CD8^+^ T‐cell response	Poillet‐Perez *et al* (2018), Poillet‐Perez *et al* (2020)
Bone cancer	Deletion of *Atg7* or *Atg5* in transplantable MCA205 cells	Resistance to chemotherapy, linked to impaired release of immunogenic danger signals and reduced antitumor T‐cell response	Michaud *et al* (2011)
Breast cancer	Conditional deletion of *Atg5* or *Atg12* in transplantable PyMT‐driven MaEC cells	Increased recurrence and size of spontaneous metastases when injected intravenously in syngeneic mice	Marsh *et al* (2020)
Breast cancer	Whole‐body allelic loss of *Becn1*	Development of spontaneous mammary tumors, linked to augmented mammary stem and progenitor cell activities	Cicchini *et al* (2014)
Breast cancer	Whole‐body deletion of *Bnip3*	Accelerated PyMT‐driven tumor initiation, progression, and metastasis, linked to mitochondrial dysfunction	Chourasia *et al* (2015)
Breast cancer	Conditional deletion of *Fip200* in PyMT‐driven MaEC cells	Reduced PyMT‐driven tumor initiation, progression, and metastasis, linked to increased IFN‐mediated T‐cell infiltration in the TME	Wei *et al* (2011)
Breast cancer	Whole‐body allelic loss of *Becn1*	Reduced pro‐tumorigenic effect associated with *Palb2* ablation in *Tp53* wild‐type mice	Huo *et al* (2013)
Breast cancer	Deletion of *Becn1* in transplantable 4T1 cells	Improved NK‐mediated tumor regression	Baginska *et al* (2013), Li *et al* (2020b)
Breast cancer	Deletion of *Lamp2* in transplantable breast cancer cells	Reduced tumor growth and formation of metastasis when injected in nude mice	Han *et al* (2017)
Breast cancer	Deletion of *Atg5* in transplantable 4T1 cells	Accelerated tumor growth and resistance to T‐cell‐mediated antitumor immunity after ICIs treatment	Li *et al* (2020b)
Breast cancer	Deletion of *Atg5* or *Atg7* in transplantable TS/A cells	Improved radiosensitivity and control of non‐irradiated lesions, linked to enhanced type I IFN‐mediated antitumor immunity	Yamazaki *et al* (2020)
Breast cancer	Conditional whole‐body deletion of *Atg5* or *Atg16L1* or *Atg14*	Reduced tumor growth of allografted syngeneic E0771 breast cancer cells, coupled with increased antitumor CD8^+^ T‐cell response	DeVorkin *et al* (2019)
Colorectal cancer	Conditional deletion of *Atg7* in intestinal epithelial cells	Reduced *Apc* loss‐driven tumor development and progression, coupled with increased antitumor CD8^+^ T‐cell response	Levy *et al* (2015)
Colorectal cancer	Deletion of *Atg7* in transplantable CT26 cells	Reduced tumor growth, linked to increased antitumor CD8^+^ T‐cell response	Arensman *et al* (2020)
Colorectal cancer	Deletion of *Atg5* or *Becn1* in transplantable CT26 cells	Resistance to radiotherapy and chemotherapy, linked to impaired release of immunogenic danger signals, and reduced antitumor T‐cell response	Michaud *et al* (2011), Ko *et al* (2014)
Hepatic tumor	Liver‐specific mosaic deletion of *Atg5* or *Atg7*	Increased number of spontaneous tumors, linked to increased p62 accumulation and dysfunctional mitochondria	Takamura *et al* (2011)
Hepatic tumor	Liver‐specific deletion of *Lamp2*	Increased tumor incidence linked to increased vulnerability to oxidative stress	Schneider *et al* (2015)
Hepatic tumor	Knock‐in of *Lamp2* in transplantable HCC cells	Increased tumor growth when injected subcutaneously in nude mice	Ding *et al* (2016)
Intestinal cancer	Intestinal epithelia cell‐specific deletion of *Stat3*	Reduced initiation of sporadic intestinal tumorigenesis linked to enhanced mitophagy	Ziegler *et al* (2018)
Lung cancer	Deletion of *Ambra1* in transplantable iMEFs	Accelerated tumor development	Cianfanelli *et al* (2015)
Lung cancer	Conditional whole‐body deletion of *Atg7* or *Atg5*	Impaired growth of allografted 71.8 NSCLC cells, linked to reduced circulating arginine	Poillet‐Perez *et al* (2018)
Lung cancer	Conditional deletion of *Atg5* in *Kras^G12D^*‐driven lung tumors	Prolonged OS linked to dysfunctional mitochondria, but accelerated tumor development linked to increased tumor infiltration by T_REG_	Rao *et al* (2014), Pietrocola *et al* (2016)
Lung cancer	Conditional deletion of *Atg7* in *Kras^G12D^*‐driven lung tumors	Prolonged OS and reduced tumor progression of established tumors, linked to dysfunctional mitochondria and reduced FA oxidation	Guo *et al* (2013), Karsli‐Uzunbas *et al* (2014)
Lung cancer	Conditional deletion of *Atg7* in *Braf^V600E^*‐driven lung tumors	Prolonged OS and reduced tumor progression due to dysfunctional mitochondria, but accelerated tumor development	Strohecker *et al* (2013)
Lung cancer	Deletion of *Lamp2* in transplantable lung cancer cells	Reduced tumor growth and formation of metastasis when injected in nude mice	Kon *et al* (2011)
Lung cancer	Knock‐in of mutant *PKM2^K305Q^* in transplantable lung cancer cells	Increased tumor growth when injected in nude mice, linked to accumulation of glycolytic intermediates	Lv *et al* (2011)
Melanoma	Conditional whole‐body deletion of *Atg7* or *Atg5* deletion	Impaired growth of allografted YUMM1.1‐9 melanoma cells, linked to reduced circulating arginine, and increased antitumor CD8^+^ T‐cell response	Poillet‐Perez *et al* (2018), Poillet‐Perez *et al* (2020, 883)
Melanoma	Conditional deletion of *Atg7* in *Braf^V600E^*‐driven, *Pten*‐competent melanomas	Reduced OS and accelerated melanoma onset	Rosenfeldt *et al* (2021)
Melanoma	Conditional deletion of *Atg7* in *Braf^V600E^*‐driven, *Pten*‐null melanomas	Prolonged OS and reduced tumor development, linked to increased oxidative stress and senescence	Xie *et al* (2015)
Melanoma	Deletion of *Becn1* in transplantable B16–F10 cells	Improved NK‐mediated tumor regression in a CCL5‐dependent manner	Baginska *et al* (2013), Mgrditchian *et al* (2017)
Melanoma	Myeloid cell‐specific deletion of *Becn1* or *Atg5*	Reduced growth of subcutaneously engrafted murine B16F10 melanoma	Cunha *et al* (2018)
Melanoma	Whole‐body deletion of *Rubcn*	Reduced growth of subcutaneously engrafted murine B16F10 melanoma	Cunha *et al* (2018)
Multiple malignancies	Whole‐body allelic loss of *Becn1*	Development of spontaneous malignancies	Qu *et al* (2003), Yue *et al* (2003)
Multiple malignancies	Whole‐body allelic loss of *Ambra1*	Development of spontaneous malignancies	Cianfanelli *et al* (2015)
Multiple malignancies	Conditional whole‐body deletion of *Atg5*	Accelerated development of spontaneous tumors after temporal autophagy inhibition	Cassidy *et al* (2020)
Multiple malignancies	Conditional whole‐body deletion of *Atg7*	Accelerated development of p53 loss‐driven spontaneous tumors	Yang *et al* (2020)
Pancreatic cancer	Deletion of *Atg5* or *Atg7* in PSCs	Delayed tumor growth of co‐injected PDAC cells linked to reduced alanine production by PSCs	Sousa *et al* (2016)
Pancreatic cancer	Conditional whole‐body Knock‐in of mutant *Atg4B^C74A^*	Tumor regression in an autochthonous mouse model of PDAC	Yang *et al* (2018)
Pancreatic cancer	Pancreas‐specific mosaic deletion of *Atg7* or *Atg5*	Accelerated *KRAS^G12D^*‐driven tumor development in the absence of p53	Rosenfeldt *et al* (2013), Yang *et al* (2014)
Pancreatic cancer	Conditional knock‐in of mutant *Atg4b^C74A^* in transplantable PDAC cells	Reduced tumor growth, linked to enhanced expression of MHC class I molecules and a potentiated antitumor CD8^+^ T‐cell response	Yamamoto *et al* (2020)
Pancreatic cancer	Conditional pancreas‐specific deletion of Bnip3l	Delayed tumor progression, linked to restauration in mitochondrial content, and improved respiratory capacity	Humpton *et al* (2019)
Prostate cancer	Conditional whole‐body deletion of *Atg5*	Reduced tumor growth of allografted syngeneic Tramp‐C2 prostate cancer cells, coupled with increased antitumor CD8^+^ T‐cell response	DeVorkin *et al* (2019)
Renal cancer	Allelic loss of *Becn1* or deletion of *Atg5* in transplantable iBMK cells	Accelerated tumor growth linked to increased p62 accumulation and dysfunctional mitochondria	Mathew *et al* (2009)

FA, fatty acid; iBMK, immortalized baby mouse kidney; iMEF: immortalized mouse embryonic fibroblast; MaEC, mammary epithelial carcinoma; NSCLC, non‐small‐cell lung cancer; OS, overall survival; PDAC, pancreatic ductal adenocarcinoma; PSC, pancreatic stellate cell; PyMT, polyoma middle tumor antigen; TME, tumor microenvironment.

### Oncosuppressive functions of autophagy: cancer initiation

*Becn1^+^*^/^^−^ mice are more susceptible to develop spontaneous or oncogene‐activation‐driven malignancies than their wild‐type counterparts (Qu *et al*, 2003; Yue *et al*, 2003; Cicchini *et al*, 2014). In addition, the appearance of (in most cases benign) tumor lesions is accelerated by the deletion of multiple genes that intercept the autophagy pathway (White, 2015; Amaravadi *et al*, 2016; Amaravadi *et al*, 2019). Examples of autophagy genes for which this has been observed include (i) systemic deletion of *Ambra1* (Cianfanelli *et al*, 2015; Di Leo *et al*, 2021; Maiani *et al*, 2021), (ii) shRNA‐dependent temporal suppression of *Atg5* expression (Cassidy *et al*, 2020), (iii) liver‐specific mosaic deletion of *Atg5* (Takamura *et al*, 2011), or (iv) conditional knockout of *Atg5* or *Atg7* in the lung and the pancreas of GEMMs (Rosenfeldt *et al*, 2013; Strohecker *et al*, 2013; Rao *et al*, 2014). Whereas in specific circumstances (i.e., *Becn1^+^* ^/^^−^ mice, or temporal suppression of Atg5 expression), derailed autophagy evokes the appearance of advanced malignancies, in other cases neoplastic lesions originating from suppressed autophagy fail to transition from the benign to the malign state. Data inferred from patients affected by primary melanoma suggest that low expression levels of *Atg5* correlate with reduced progression‐free survival. Of note, *Atg5* downregulation hinders the induction of oncogene‐induced senescence promoting BRAF^V600E^‐driven melanogenesis *in vitro* (Liu *et al*, 2013b). As further corroboration of this result, deletion of *Atg7* accelerates melanogenesis in animals in which the expression of BRAF^V600E^ is restricted to the skin, depending upon the expression of functional *Pten* (phosphatase and tensin homolog) (Rosenfeldt *et al*, 2021).

In evaluating the sum total of these preclinical findings, the implications are that for patients who are treated with chemical autophagy inhibitors, it is unlikely that secondary cancers will arise during the earliest stages of treatment, but monitoring for polyp formation in certain organs may need to be considered if autophagy inhibitors are used for longer periods of time or as chemoprevention agents.

Autophagy‐dependent removal of selective organelles has been also linked to tumor‐preventive functions (Miller & Thorburn, 2021). As an example, the mitophagy regulator BNIP3 limits the formation and progression of primary polyomavirus middle T antigen/PyMT‐driven mammary tumors in mice (Chourasia *et al*, 2015). Recently, selective autophagy has also been reported to prevent genomic instability derived by aberrant mitoses, which are frequent in tumors. In this case, autophagy selectively targets the non‐membranous organelles centriolar satellites, which safeguard mitosis accuracy by preserving centrosome integrity (Holdgaard *et al*, 2019). In addition, alternative autophagy routes participate in the tumor‐preventive action of the autophagy pathway. Growing evidence supports the idea that chaperone‐mediated autophagy (CMA) contributes to the prevention of cellular malignant transformation under physiological conditions. Indeed, mouse models with selective blockage of CMA in the liver result in higher rates of malignant transformation in this organ (Schneider *et al*, 2015). CMA protects against oncogenic transformation, on the one hand by actively promoting degradation of pro‐oncogenic proteins such as MYC (MYC proto‐oncogene, bHLH transcription factor) (Gomes *et al*, 2017), TPT1/TCTP (tumor protein, translationally controlled 1) (Bonhoure *et al*, 2017), or MDM2 (Lu *et al*, 2010), and on the other hand by contributing to the immuno‐oncogenic response (Garg *et al*, 2013).

Besides the well‐recognized capacity to safeguard the homeostasis of parenchymal cells, it appears plausible to speculate that part of the oncosuppressive functions of autophagy are due to its ability to attenuate the inflammatory response (Zhong *et al*, 2016; Monkkonen & Debnath, 2018). In particular, autophagy counteracts the establishment of an inflammatory microenvironment (i) by disposing of dysfunctional mitochondria and the oxidatively damaged proteome (Cannizzo *et al*, 2012; Palikaras *et al*, 2018) and reducing SQSTM1/p62 accumulation (Mathew *et al*, 2009; Moscat *et al*, 2016), therefore dampening aberrant intracellular ROS burden, or (ii) by degrading inflammasomes (which are required for the maturation and secretion of IL1B/IL1β and IL18), or preventing their activation (e.g., through the elimination of cytosolic mtDNA) (Lamkanfi & Dixit, 2014; Matsuzawa‐Ishimoto *et al*, 2018). In addition, proficient mitophagy appears to be required to stimulate CD8^+^ T‐cell‐dependent immunity in the context of intestinal tumorigenesis, thereby enabling the establishment of anticancer immunosurveillance over pre‐cancerous lesions (Ziegler *et al*, 2018; Rao *et al*, 2019).

### Tumor‐promoting functions of autophagy: cancer initiation

Although the experimental lines of evidence mentioned above support the concept that autophagy limits neoplastic transformation, notable exceptions to this paradigm have been described. As an example, conditional deletion of the gene coding for the ULK1/Atg1 interactor RB1CC1/FIP200 in mammalian epithelial cells restrains the growth of mammary carcinoma tumors induced by polyomavirus middle T antigen, associated with the induction of a prominent type I IFN response (Wei *et al*, 2011). Likewise, allelic loss of *Becn1* suppresses the pro‐tumorigenic effect linked to the loss of the hereditary breast cancer susceptibility gene *Palb2* (partner and localizer of BRCA2), in the presence of an intact TP53 signaling (Huo *et al*, 2013). In addition, conditions of “leaky gut” associated with the conditional ablation of *Atg7* in epithelial colon cells predispose a local immune response that is instrumental for limiting the number of pre‐tumoral lesions in *Apc^+^* ^/^^−^ colonocytes (Levy *et al*, 2015). Consistently, CT26 cells knocked out for *Atg7* show increased expression of chemokines involved in the recruitment of CD8^+^ T lymphocytes, and depletion of CD8^+^ T cells significantly restores the growth of tumors in immunocompetent hosts (Arensman *et al*, 2020).

### Tumor‐promoting functions of autophagy: cancer progression

Compelling evidence obtained from a large variety GEMMs of cancer contributed to advocate the hypothesis that autophagy is required to sustain the increasing metabolic demand of cancer cells during the earliest stages of neoplastic transformation, explaining why the genetic inhibition of autophagy in malignant cells restrains progression from normal to benign tumors and arrests it into a benign state (Galluzzi *et al*, 2015b; Kimmelman & White, 2017). Such an effect seems to occur irrespectively of cancer type and driver mutation, as it has been documented in preclinical models of lung and pancreatic ductal carcinomas driven by *Kras^G12D^* (Guo *et al*, 2013; Rosenfeldt *et al*, 2013; Rao *et al*, 2014; Yang *et al*, 2014), *Braf^V600E^*‐driven lung cancer (Strohecker *et al*, 2013), and melanoma (upon simultaneous loss of *Pten*) (Xie *et al*, 2015). In the context of *Kras ^G12D^*‐driven pancreatic ductal carcinoma (PDAC), pharmacological inhibition of KRAS or its downstream effector MAPK1/ERK2 (mitogen‐activated protein kinase 1) further increases the autophagic flux, while enhancing the dependency of cancer cells to intact autophagy (Bryant *et al*, 2019; Kinsey *et al*, 2019). Therefore, pharmacological inhibition of autophagy by chloroquine or genetic suppression of autophagy synergistically improves the efficacy of MAPK/ERK inhibitors in restraining PDAC progression (Bryant *et al*, 2019). Autophagy‐deficient tumor lesions are peculiarly characterized by the inability to process and oxidize metabolic substrates (e.g., glutamine, fatty acids) within mitochondria, suggesting that autophagy preserves the metabolic fitness of malignant cells via proficient mitophagy (Karsli‐Uzunbas *et al*, 2014; Kimmelman & White, 2017; Poillet‐Perez & White, 2019; Vara‐Perez *et al*, 2019). In this scenario, accumulating evidence supports the tenet that the removal of specific organelles (Miller & Thorburn, 2021) or proteins (Deng *et al*, 2021) via autophagy contributes to the tumor‐supportive function of autophagy in established tumor lesions. Of note, while deletion of essential autophagic genes impairs the outgrowing performance of cancer cells, autophagy‐deficient tumors evolve the capacity to bypass autophagy loss via the upregulation of NFE2L2/NRF2. Importantly, NFE2L2/NRF2 activation appears to compensate for the loss of proteostasis imposed on neoplastic cells by autophagy deficiency, yet renders autophagy‐deficient cells more sensitive to proteasomal inhibition (Towers *et al*, 2019).

A pro‐oncogenic mechanism has also been described for CMA in established tumor lesions (Arias & Cuervo, 2020). Most types of solid tumor cells display abnormally upregulated levels of CMA that are required to sustain tumor growth (Kon *et al*, 2011; Ding *et al*, 2016; Han *et al*, 2017). Multiple mechanisms seem to contribute to this pro‐tumorigenic function of CMA including the participation of CMA in the regulation of cancer cellular energetics (Kon *et al*, 2011; Lv *et al*, 2011; Xia *et al*, 2015), protein translation (Hao *et al*, 2019) and cell cycle (Hubbi *et al*, 2014; Zhou *et al*, 2016), the direct degradation by CMA of antitumoral proteins such as RND3 (Rho family GTPase 3) or MCL1 (MCL1 apoptosis regulator, BCL2 family member) (Zhou *et al*, 2016; Suzuki *et al*, 2017), and the participation of CMA in the cellular response to stressors (Ali *et al*, 2011; Saha, 2012; Hubbi *et al*, 2013). CMA in cells within the TME has also recently been shown to contribute to tumorigenesis (Valdor *et al*, 2019; Wang *et al*, 2019) although the specific mechanisms require future clarification. Targeting CMA in cancer is gaining growing interest since the development of drugs that selectively activate this type of autophagy (Anguiano *et al*, 2013) that could be used preventively in situation at risk of transformation; some groups have even proposed utilizing further upregulation of CMA in cancer to induce a metabolic crisis (Xia *et al*, 2015). However, because in more cancer types, experimental blockage of CMA has demonstrated to efficiently reduce the tumor size, efforts are now focused on development of drugs capable of selectively inhibiting CMA.

### Autophagy in anticancer immunosurveillance

As discussed above, autophagy operates at the interface between the transformed and non‐transformed compartments of the tumor. Interestingly, perturbations in the autophagic flux paradoxically enable malignant cells to bypass immune system‐mediated control or instead impose on tumor cells a superior control by the immune system, in a highly context‐dependent fashion. Extracellular release of KRAS^G12D^ by cancer cells succumbing to autophagy‐dependent ferroptosis is essential for pancreatic tumor‐associated macrophages (TAM) to switch to an “M2‐like” immunosuppressive phenotype (Dai *et al*, 2020). Importantly, M2 TAMs have been linked to tumor progression, metastases (Han *et al*, 2021), and resistance to conventional chemotherapeutics (Larionova *et al*, 2019) in multiple tumors. Consistent with this finding, chloroquine and its derivative hydroxychloroquine improve TAM‐mediated anticancer immune response by promoting the establishment of an “M1‐like” phenotype (Chen *et al*, 2018a; Sharma *et al*, 2020).

Pancreatic ductal carcinoma tumors expressing an ATG4B dominant‐negative mutant exhibit increased sensitivity to CD8^+^ cytotoxic T lymphocyte (CTL)‐mediated lysis (Yamamoto *et al*, 2020). Of note, PDAC cells in which autophagy is inhibited show an increased expression of MHC class I molecules at the surface, improving antigen presentation. This study found that MHC class I molecules are specific autophagy substrates. Therefore, autophagy promotes immune evasion via the lysosomal degradation of MHC class I molecule (Yamamoto *et al*, 2020). Consistently, *Atg5* deficiency promotes the formation of effector memory CD8^+^ T cells, resulting in production of higher levels of IFNG and TNF/TNF‐α and enhanced tumor rejection (DeVorkin *et al*, 2019). In addition, autophagy restrains anticancer immune response in highly antigenic tumors by limiting a STING1‐dependent type I IFN response, thereby reducing T‐cell infiltration (Poillet‐Perez *et al*, 2020). Similarly, enhanced levels of autophagy in malignant cells are favored by a hypoxic environment, which in turn correlates with increased resistance of tumor cells to natural killer (NK)‐mediated lysis through multipronged mechanisms (Baginska *et al*, 2013; Tittarelli *et al*, 2015). Inhibition of autophagy (i.e., by shRNA silencing *Becn1*) induces a massive CCL5‐dependent infiltration of NK cells into melanoma tumors, thereby reducing tumor volume (Mgrditchian *et al*, 2017). In addition, loss of autophagy in the tumor or in the host modulates the intra‐tumoral infiltration of regulatory T (T_REG_) cells (Ladoire *et al*, 2016; Poillet‐Perez *et al*, 2020), which are associated with poor disease outcome in cohorts of patients affected by multiple tumor types (Tanchot *et al*, 2013). Administration of lysosomotropic agents (e.g., hydroxychloroquine) boosts the activity of an immune checkpoint inhibitor in preclinical models of melanoma (Sharma *et al*, 2020). Similarly, chloroquine also phenocopies the effect of an ATG4B dominant‐negative mutant in PDAC cells by restoring the surface expression of MHC class I molecules and synergizes with immune checkpoint blockade treatment in restraining PDAC outgrowth (Yamamoto *et al*, 2020). This result has been further reinforced in a CRISPR‐Cas9 screen performed across multiple cell lines, indicating that autophagy proficiency entails the inherent ability to evade immune detection (Lawson *et al*, 2020). Supporting this finding, lysosomotropic agents or small molecules targeting the PtdIns3K PIK3C3/VSP34 have been efficiently combined with therapeutic regimens that promote the activation of the immune system against cancer cells (Janji *et al*, 2020; Noman *et al*, 2020). Along similar lines, pharmacological or genetic inhibition of autophagy in syngeneic TS/A breast cancer models is sufficient to enhance the secretion of type I IFN by tumor cells exposed to focal radiation (Yamazaki *et al*, 2020). This effect follows the mtDNA‐mediated activation of the cGAS (cyclic GMP‐AMP synthase)‐STING1 pathway and in turn promotes long‐lasting local and systemic immunosurveillance (Vanpouille‐Box *et al*, 2018; Sprooten *et al*, 2019; Yamazaki *et al*, 2020).

Autophagy‐independent functions of the ATG machinery have also been implicated in the crosstalk between immune and cancer cells. As an example, functional LAP in myeloid cells supports tumor progression by promoting the establishment of an immune tolerant microenvironment upon phagocytosis of dying tumor cells, which eventually hinders T‐cell activation. Accordingly, genetic suppression of LAP in myeloid cells enables an improved immune control over tumor outgrowth (Cunha *et al*, 2018). In addition, the extracellular release of potassium by dying cancer cells leads to the induction of autophagy in CD8^+^ T cells, thus resulting in the acquisition of a stem cell‐like phenotype and ultimately improving tumor clearance. This effect can be further potentiated by treatment with caloric restriction mimetics (Vodnala *et al*, 2019), thus suggesting dietary interventions stimulating autophagy can be combined with certain antineoplastic therapies to achieve durable anticancer immunosurveillance (Levesque *et al*, 2019b; Pietrocola & Kroemer, 2019).

In contrast to these findings, intact autophagy responses regulate (i) the adjuvanticity (e.g., the capacity to emit danger signals that are preliminary to the recruitment of immune cells to the tumor bed) (Michaud *et al*, 2011; Zitvogel *et al*, 2015; Garg *et al*, 2016) and (ii) antigenicity of tumor cells (Caron *et al*, 2011; Ma *et al*, 2013c; Pietrocola *et al*, 2017), thereby promoting the establishment of the cancer‐immunity cycle leading to the CTL‐dependent elimination of malignant cells (Yamazaki *et al*, 2020). In line with this observation, autophagy‐deficient tumors transplanted into immunocompetent mice escape immunosurveillance, due to their inability to secrete immunostimulatory ATP (Michaud *et al*, 2011), and the absence of markers of autophagy (i.e., LC3B) in cancer cells has been correlated to reduced intra‐tumoral infiltration of CTLs (but higher infiltration of T_REG_s and CD68^+^ macrophages) and poor prognosis in women with breast cancer (Ladoire *et al*, 2016). In addition, in this setting, functional autophagy accounts for the ability of selected chemotherapeutics to elicit immunogenic cell death (Galluzzi *et al*, 2015a; Galluzzi *et al*, 2020b), an effect that is intimately related to the autophagy‐dependent release of ATP in the tumor bed (Kroemer *et al*, 2013; Martins *et al*, 2014; Galluzzi *et al*, 2017d) and that in turn promotes the recruitment of DC precursors and the priming of antitumor T cells (Ma *et al*, 2013b; Lee & Radford, 2019; Martinek *et al*, 2019; Galluzzi *et al*, 2020a). Of note, overactivation of autophagy by time‐restricted fasting or fasting mimetic agents potentiates the anticancer activity of immunogenic cell death inducers when used as a standalone regimen (Pietrocola *et al*, 2016; Galluzzi *et al*, 2017b; Castoldi *et al*, 2019) or in combination with antibodies targeting CTLA4 (cytotoxic T lymphocyte‐associated protein 4) or the immunosuppressive molecule CD274/PD‐L1 (Levesque *et al*, 2019a). Likewise, defective autophagy underlies the increased resistance of triple‐negative breast cancer cells to CTL lysis after immune checkpoint blocker treatment (Li *et al*, 2020b), while reducing the radiosensitivity of colorectal CT26 tumors transplanted into immunocompetent (but not immunodeficient) hosts (Ko *et al*, 2014).

### Autophagy and cancer: clinical implications

Targeting autophagy‐dependent vulnerabilities of cancer cells has progressively gained attraction in the last decade, strongly advocating for the use of autophagy inhibitors (Amaravadi *et al*, 2019) in combination with regimens of targeted therapy (Bryant *et al*, 2019; Liu *et al*, 2020a), radiotherapy (Yamazaki *et al*, 2020), and immunotherapy (Galluzzi *et al*, 2018a; Yamamoto *et al*, 2020; Xia *et al*, 2021). Conditional deletion of autophagy essential genes in the host curtails the availability of metabolic substrates for hyperproliferating tumor cells, thereby impairing tumor progression (Karsli‐Uzunbas *et al*, 2014; Poillet‐Perez *et al*, 2018; Poillet‐Perez & White, 2019).

In this scenario, the field would certainly benefit from the expansion of the pharmacological toolbox to restrain autophagy in established neoplasia (Egan *et al*, 2015), in light of the limited specificity of autophagy inhibitors used in clinics (Manic *et al*, 2014). In addition to this aspect, further analyses performed in human studies are in need to assess the safety profile of prolonged/systemic inhibition of autophagy, as stable or transient inhibition of autophagy not only can limit antitumor immune responses mediated by chemotherapy, radiation therapy (Galluzzi *et al*, 2017b; Galluzzi *et al*, 2020a), and/or targeted therapy (Petroni *et al*, 2021), but may accelerate organismal decay (Guo *et al*, 2013; Yang *et al*, 2020), while precipitating episodes of secondary transformation (Cassidy *et al*, 2020). Hence, it is tempting to speculate that research efforts will be re‐energized toward the implementation of pharmacological modalities to selectively modulate autophagy in the transformed compartment.

The translation of autophagy‐targeted therapy into the clinic has just begun. Data from clinical studies are needed to clarify to which degree autophagy is active in specific tumors, either at the basal level or in response to distinct anticancer regimens. Owing to the high context‐dependency of the autophagy pathway in cancer, therapy‐oriented decisions based on autophagy modulation can only be adopted by taking into consideration the type and stage of tumor, and host‐related characteristics.

## Immunity to pathogens, autoimmunity, and inflammation

Autophagy, or selected ATG functional modules, participates in the defensive response to pathogen invasion. Robust evidence demonstrates that maneuvers that hamper the autophagy reaction predispose cells to specific bacterial, protozoan, viral, or fungal infections (Levine *et al*, 2011; Gomes & Dikic, 2014; Keller *et al*, 2020b) (Table 9). The causes underlying the accrued propensity of autophagy‐incompetent cells to microbial infections lay in the multitude of actions exerted by the autophagic machinery within specialized (i.e., adaptive and innate immune cells) and parenchymal cells (Ma *et al*, 2013c; Clarke & Simon, 2019; Deretic, 2021). First, autophagy mediates quintessential (and cell type defining) functions in virtually all the immune cell subtypes, both at sites of hematopoiesis and in peripheral tissues (Ma *et al*, 2013c; Clarke & Simon, 2019). Accordingly, autophagy deficiency affects generation, survival, maturation, and effector properties of central cellular components of innate and adaptive immunity (Ma *et al*, 2013c; Clarke & Simon, 2019; Deretic, 2021). Second, impaired autophagy responses undermine the capacity of infected cells to dispose of invading pathogens (or components thereof) within the lysosome (Levine *et al*, 2011; Gomes & Dikic, 2014; Keller *et al*, 2020b; Deretic, 2021). Pathogen invasion entails the activation of bulk or selective autophagy modalities as a first‐line defense strategy. Nonetheless, infectious microorganisms utilize evasive strategies to bypass autophagy‐dependent degradation, or even subvert autophagosomal membranes as a preferential replication site (Gomes & Dikic, 2014). In addition, certain intracellular parasites such as *Toxoplasma gondii* or bacteria such as *Francisella tularensis* hijack host autophagy to harness nutrients they are auxotrophic for, such as fatty acids or amino acids (Steele *et al*, 2013; Pernas *et al*, 2018). Third, instances of derailed autophagy exacerbate the organismal response to infection, as it alters the extinction of the inflammatory cascade, thereby exacerbating the noxious local and systemic effects tied to invading pathogen infection (Deretic, 2021).

**Table 9:** Immunity, inflammation, and immune‐related disorders associated with genetic intervention of autophagy in mice.

Setting	Genetic intervention	Effects on disease phenotype	Ref.
Bacterial infection	Myeloid cell‐specific deletion of *Atg5*	Enhanced susceptibility to infection mediated by *Mycobacterium tuberculosis*	Watson *et al* (2012), Kimmey *et al* (2015)
Bacterial infection	Whole‐body deletion of *Prkn*	Enhanced susceptibility to infection mediated by *Mycobacterium tuberculosis*	Manzanillo *et al* (2013)
Bacterial infection	Myeloid cell‐specific deletion of *Atg7*	Abrogated autophagic killing of *Mycobacterium tuberculosis* var. *bovis*	Pilli *et al* (2012)
Bacterial infection	Conditional myeloid cell‐specific knock‐in of mutant *Mcu* ^Δmye^	Improved control of *Listeria monocytogenes* infection, linked to enhanced LAP formation improved	Li *et al* (2021)
Bacterial infection	Intestinal epithelial cell‐specific deletion of *Atg16l1*	Enhanced susceptibility to infection mediated by *Listeria monocytogenes*	Tan *et al* (2018)
Bacterial infection	Whole‐body deletion of *Map1lc3b* or knock‐in of hypomorphic *Atg16l1*	Enhanced susceptibility to systemic and lung infection mediated by *Staphylococcus aureus*	Maurer *et al* (2015), Keller *et al* (2020a)
Bacterial infection	Endothelial cell deletion of *Atg16l1*	Enhanced lethality due to exacerbated susceptibility to systemic and lung infection mediated by *Staphylococcus aureus*	Maurer *et al* (2015)
Bacterial infection	T‐cell‐specific deletion of *Lamp2*	Impaired adaptive response to immunization with OVA peptide or *Listeria* infection	Valdor *et al* (2014)
Fungal infection	Whole‐body deletion of *Rubcn*	Enhanced susceptibility to infection mediated by *Aspergillus fumigatus* and granuloma formation, linked to increased pro‐inflammatory cytokines secretion	Martinez *et al* (2015)
Fungal infection	Myeloid cell‐specific deletion of *Becn1* or *Atg7*	Enhanced susceptibility to infection mediated by *A. fumigatus* and granuloma formation, linked to increased pro‐inflammatory cytokines secretion	Martinez *et al* (2015)
IBD	Whole‐body knock‐in of mutant *Atg16l1^T316A^*	Impaired clearance of the ileal pathogen *Y. enterocolitica* and elevated inflammatory cytokine response	Lassen *et al* (2014), Murthy *et al* (2014), Bel *et al* (2017)
IBD	Whole‐body knock‐in of hypomorphic *Atg16l1*	Disruption of the Paneth cell granule exocytosis pathway and enhanced susceptibility to infection by commensal MNV	Cadwell *et al* (2008), Cadwell *et al* (2009), Cadwell *et al* (2010)
IBD	IEC‐specific deletion of *Atg5*	Disruption of the Paneth cell granule exocytosis pathway linked to impaired lipid metabolism	Cadwell *et al* (2008)
IBD	IEC‐specific deletion of *Atg16l1*	More severe colon histopathology and increased susceptibility to GVHD	Matsuzawa‐Ishimoto *et al* (2017), Aden *et al* (2018), Pott *et al* (2018)
IBD	IEC‐specific deletion of *Tsc1*	Disrupted intestinal homeostasis and highly susceptibility to DSS‐induced colitis	Xie *et al* (2020)
IBD	IEC‐specific co‐deletion of *Atg7* and *Xbp1*	Worsening of Crohn disease‐like ileitis linked to defective ER stress response	Adolph *et al* (2013)
IBD	IEC‐specific co‐deletion of *Atg16l1* and *Xbp1*	Worsening of Crohn disease‐like ileitis linked to defective ER stress response	Adolph *et al* (2013), Aden *et al* (2018)
IBD	T‐cell‐specific deletion of *Atg16l1*	Development of spontaneous intestinal inflammation	Kabat *et al* (2016)
IBD	CD4^+^ T‐cell‐specific deletion of *Atg16l1*	Increased susceptibility to T‐cell‐mediated experimental IBD and elevated T_H_2‐mediated responses	Kabat *et al* (2016)
IBD	FOXP3^+^ T‐cell‐specific deletion of *Atg16l1*	Development of spontaneous multiorgan inflammation	Kabat *et al* (2016)
IBD	CD11c^+^ DC‐specific deletion of *Atg16l1*	Increased susceptibility to *Bacteroides fragilis*‐mediated colitis, linked to reduced induction of T_REG_ cells	Chu *et al* (2016)
Lung fibrosis	Whole‐body deletion of *Atg4b*	Exacerbated bleomycin‐induced lung fibrosis, linked to alterations in pro‐inflammatory cytokines, and increased neutrophilic infiltration	Cabrera *et al* (2015)
Multiple sclerosis	Conditional CD11c^+^ DC‐specific deletion of *Atg5*	Reduced development of EAE linked to limited CNS accumulation of CD4^+^ T cells	Keller *et al* (2017)
Multiple sclerosis	CD11c^+^ DC‐specific deletion of *Atg7*	Reduced incidence and severity of EAE by reducing CD4^+^ T‐cell priming	Bhattacharya *et al* (2014)
Multiple sclerosis	Microglia‐specific deletion of *Atg7*	Increased accumulation of phagocytosed myelin and lack of recovery from multiple sclerosis‐like disease	Berglund *et al* (2020)
SLE	B cell‐specific deletion of *Atg5*	Extended OS and reduced markers of SLE in *Tlr7.1* transgenic mice	Weindel *et al* (2015)
SLE	DC‐specific deletion of *Atg5*	Extended OS and reduced markers of SLE in *Tlr7.1* transgenic mice	Weindel *et al* (2017)
SLE	DC and B cell‐specific deletion of *Atg5*	Development of a rapid and lethal inflammatory condition in *Tlr7.1* transgenic mice	Weindel *et al* (2017)
SLE	Whole‐body deletion of *Nox2* or *Rubcn*	Development of symptoms of autoinflammatory disorder	Martinez *et al* (2016)
SLE	Whole‐body deletion of *Nox2* or *Rubcn*	Development of symptoms of autoinflammatory disorder	Martinez *et al* (2016)
Viral infection	Neuron‐specific deletion of *Atg5*	Increased susceptibility of neonatal mice to lethal CNS infection with SIN	Orvedahl *et al* (2010)
Viral infection	Whole‐body deletion of *Fancc*	Increased susceptibility to lethal CNS infection with SIN or HSV‐1, after mitophagy inhibition	Sumpter *et al* (2016)
Viral infection	Whole‐body deletion of *Snx5*	Increased susceptibility of neonatal mice to lethal CNS infection with SIN, CHIKV, or WNV, after virus‐induced autophagy inhibition	Dong *et al* (2021b)
Viral infection	Whole‐body knock‐in of mutant *Atg16l1^E230^*	Increased susceptibility low‐pathogenicity IAV, exacerbated pneumonia, and high mortality, after LAP inhibition	Wang *et al* (2021)
Viral infection	Conditional activated CD8^+^ T‐cell‐specific deletion of *Atg7* or *Atg5*	Impaired CD8^+^ T‐cell memory formation in response to chronic LCMV infection	Wang *et al* (2021)
Viral infection	Conditional CD11c^+^ cDC‐specific deletion of *Atg5*	Increased susceptibility to HSV‐2 infection, linked to impaired antigen presentation and CD4^+^ T‐cell priming by cDCs	Lee *et al* (2010a)
Viral infection	T‐cell‐specific deletion of *Atg7*	Impaired CD8^+^ T‐cell memory formation in response to MCMV infection	Wang *et al* (2021)
Viral infection	Pancreatic acinar cell‐specific deletion of *Atg5*	Reduced CVB3 titer in the pancreas and diminished pancreatic pathology	Alirezaei *et al* (2012)
Viral infection	Whole‐body knock‐in of hypomorphic *Atg16l1*	Limited ZIKV vertical transmission and placental and fetal damage in pregnant mice	Alirezaei *et al* (2012)

CHIKV, chikungunya virus; CNS, central nervous system; CVB3, coxsackievirus B3; cDC, conventional dendritic cell; DSS, dextran sulfate sodium; EAE, experimental autoimmune encephalomyelitis; GVHD, graft‐versus‐host disease; HSV, herpes simplex virus; IAV, influenza A virus; IEC, intestinal epithelial cell; LCMV, lymphocytic choriomeningitis virus; MCMV, murine cytomegalovirus; MNV, murine norovirus; OVA, ovalbumin; SIN, Sindbis virus; SLE, systemic lupus erythematous; WNV, West Nile virus; ZIKV, Zika virus

### Bacterial infections

A large variety of bacterial species with intracellular tropism (including *Shigella flexneri*, *Listeria monocytogenes* and *Group A Streptococcus*) are targeted for autophagy‐mediated elimination (Gomes & Dikic, 2014; Keller *et al*, 2020b). From a mere cell autonomous standpoint, the autophagosome‐generating machinery perceives intracellular microbes of bacterial origin (especially those escaping their membranes of internalization) as a substrate, thereby triggering a selective form of autophagy known as “xenophagy”, which has been extensively typified for infections mediated by *Salmonella enterica* serovar Typhimurium (Birmingham *et al*, 2006) or *Mycobacterium tuberculosis* (Gutierrez *et al*, 2004; Watson *et al*, 2012). In the context of *M. tuberculosis* infection, a positive correlation has been established between successful IFNG and IL17A antibacterial immune response and levels of autophagy in patients (Rovetta *et al*, 2014; Tateosian *et al*, 2017). Along similar lines, *M. tuberculosis*‐induced expression of signaling lymphocytic activation molecule family member 1 (SLAMF1) contributes to the activation of autophagy in neutrophils (Pellegrini *et al*, 2020). Pattern‐recognition receptor sensing of bacterial components is instrumental for the ignition of the autophagy cascade that leads to the sequestration of intracellular pathogens within autophagosomes. As an example, the interaction of lipopolysaccharide with TLR4 precedes the autophagy‐mediated engulfment of *Salmonella* Typhimurium (Liu *et al*, 2019). Likewise, MYD88 (myeloid differentiation primary response gene 88)‐ and TICAM1/TRIF (Toll‐like receptor adaptor molecule 1)‐dependent signaling downstream of TLR activation causes the dissociation of BECN1 from BCL2, hence triggering xenophagy in macrophages (Shi & Kehrl, 2008). Cardiolipin, which recruits LC3 during mitophagy (Chu *et al*, 2013), contributes to Shigella xenophagy by recruiting septins that form cages colocalizing with LC3 (Krokowski *et al*, 2018).

Along similar lines, detection of cytosolic peptidoglycans by NOD1 (nucleotide‐binding oligomerization domain containing 1) and NOD2 enables the spatiotemporal coordinated localization of the autophagy machinery at the site of bacterial ingress (Travassos *et al*, 2010). The mechanistic underpinnings of xenophagy appear to recapitulate key fundamentals of PRKN‐dependent mitophagy, in that host E3 ubiquitin ligases (including PRKN, SMURF1 [SMAD‐specific E3 ubiquitin protein ligase 1] and LRSAM1 [leucine‐rich repeat and sterile alpha motif containing 1]) (Huett *et al*, 2012; Manzanillo *et al*, 2013; Fiskin *et al*, 2016) and linear ubiquitin chain assembly complex (LUBAC) catalyze the ubiquitination of cytoplasmic bacteria prior to their interaction with autophagy receptors, such as SQSTM1/p62 and CALCOCO2 (Fiskin *et al*, 2016; Noad *et al*, 2017; van Wijk *et al*, 2017). Corroborating this finding, *prkn* knockout mice are more sensitive to *M*. *tuberculosis* infection than their wild‐type littermates (Manzanillo *et al*, 2013). Importantly, exposure to LGALS8/galectin‐8 (evoked by pathogen‐induced phagosomal membrane rupture) is preparatory for the recognition by CALCOCO2, which in turn enables the autophagy‐regulated disposal of pathogen‐leaky vacuoles (Thurston *et al*, 2012). In contrast with this finding, *Coxiella burnetii* promotes the recruitment of the autophagy machinery to reseal intracellular damaged membranes (Mansilla Pareja *et al*, 2017).

In settings of *S*. Typhimurium infection, TLR4‐dependent activation of xenophagy involves the sequential activation of ULK1 by MAP3K7/TAK1 (mitogen‐activated protein kinase kinase kinase 7) (Liu *et al*, 2019) and TBK1‐dependent phosphorylation of OPTN, which augments its binding to ubiquitin‐decorated bacteria (Wild *et al*, 2011). A similar sequence of events occurs upon infection of macrophages with *M. tuberculosis*, after the STING1‐dependent recognition of extracellular DNA (Watson *et al*, 2012) and the subsequent recruitment of SQSTM1/p62, CALCOCO2, and TBK1 (Pilli *et al*, 2012). Although pattern‐recognition receptor activation triggers cytoprotective autophagy, the stimulation of autophagy is instrumental to prevent excessive IL1B production by sequestering lipopolysaccharide and preventing its recognition in the cytosol through the CASP4/CASP11 (caspase 4, apoptosis‐related cysteine peptidase) inflammasome (Meunier *et al*, 2014).

Intracellular pathogens have elaborated a variety of mechanisms to evade xenophagy (Mestre *et al*, 2010; Gomes & Dikic, 2014; Cong *et al*, 2020; Keller *et al*, 2020b; Gauron *et al*, 2021). For example, *Salmonella* and mycobacteria restrain the maturation of the phagosome, in order to foster their replication. In the case of *L. monocytogenes* (Birmingham *et al*, 2008) or Legionella (Yang *et al*, 2017a), evasive modalities involve the production of virulence factors that inactivate key components of the ATG machinery, blocking their recruitment to pathogen‐containing vacuoles (Gomes & Dikic, 2014; Cong *et al*, 2020). More recently, it has been reported that *L. monocytogenes* retains the capacity to subvert LAP (through modulation of mitochondrial calcium signaling), as a survival strategy (Li *et al*, 2021).

The induction of canonical autophagy pathway promotes the survival of cells exposed to pore forming cytolysin produced by *Vibrio cholerae* (Gutierrez *et al*, 2007). However, the functions of ATG proteins in non‐canonical processes participate in the immune response against pathogens (Mauthe & Reggiori, 2016). For instance, ATG5 mediates exclusive instances of cell death in neutrophils upon infection by *M*. *tuberculosis* (Kimmey *et al*, 2015). Autophagy‐independent functions of the ATG16L1 complex limit cell‐to‐cell spreading of *L. monocytogenes* infections by repairing listeriolysin O‐mediated rupture in the plasma membrane (Tan *et al*, 2018) and protect cells from α‐toxin‐dependent cytolysis in the context of *Staphylococcus aureus* infection (Maurer *et al*, 2015). In addition to soluble cargo such as IL1B and Aβ, ATG proteins mediate the secretion of toxin‐binding transmembrane receptors through extracellular vesicles in response to bacteria (Keller *et al*, 2020a). Of note, in phagocytic cells several components of the ATG machinery contribute to the internalization and elimination of microbes by participating in the LAP pathway in phagocytic cells (Martinez *et al*, 2015; Cunha *et al*, 2018; Galluzzi & Green, 2019; Heckmann & Green, 2019; Li *et al*, 2021). Unlike canonical autophagy, LAP acquires significant relevance for microbial cargos originating from the extracellular space, and it is thought to boost the rate of delivery of engulfed pathogens to the lysosome, after extracellular TLR stimulation, while simultaneously enabling cytokine production and antigen presentation in myeloid cells (Henault *et al*, 2012; Cunha *et al*, 2018; Galluzzi & Green, 2019; Heckmann & Green, 2019).

### Viral infections

Whereas the mechanistic insights of xenophagy have extensively been characterized in the context of bacterial infections, viruses are also targeted for autophagy‐dependent degradation, often referred to as virophagy (Choi *et al*, 2018; Cong *et al*, 2020). Virophagy has been typified by the lysosomal degradation of the Sindbis virus capsid upon interaction with SQSTM1/p62, an event that is required to protect neurons from virus‐induced death (Orvedahl *et al*, 2010; Sumpter *et al*, 2016). As discussed above in the context of bacterial infections, the selection of the viral cargo impinges on the usage of factors involved in the mitophagic process, including Fanconi anemia‐related proteins (Sumpter *et al*, 2016). Recently, a genome‐wide siRNA screening identified the endosomal protein SNX5 (sorting nexin 5) as an essential factor for virus‐induced autophagy, and knockout of *Snx5* in mice enhances lethality in response to infection by several human viruses (Dong *et al*, 2021b). Supporting the notion that autophagy enables cells to cope with viral infections, interventions that stimulate the autophagy reaction (such as the administration of the Tat‐Beclin 1 peptide) reduce the viral load and enhance the survival of mice infected by chikungunya and West Nile virus (Shoji‐Kawata *et al*, 2013). Besides enhancing the resistance of parenchymal cells to virus‐induced death, the induction of autophagy, which occurs downstream of viral sensing modules (including MAVS [mitochondrial antiviral signaling protein], implicated in cytosolic RNA detection, and STING1), concurrently restrains the excessive activation of type I IFN‐ and IL1B‐dependent signaling pathways, thus limiting tissue‐injury effects linked to an over‐persistent immune response (Cadwell, 2016; Choi *et al*, 2018; Matsuzawa‐Ishimoto *et al*, 2018). Conversely, systemic loss of the wild‐type linker domain of ATG16L1 makes mice more sensitive to lethal influenza A virus, due to LAP deficiency and reduced IFN signaling (Wang *et al*, 2021). Of note, accumulating evidence shows that the production of type I IFN can be influenced by ER stress/UPR during viral infections (Sprooten & Garg, 2020) and that downregulation of autophagy and LAP in leukocytes involved in the adaptive immune response to viral pathogens renders mice susceptible to viral infections. As an example, obliteration of *Atg5* in ITGAX/CD11c^+^ antigen‐presenting cells hinders the efficient presentation of herpes simplex virus type 1 (HSV‐1)‐associated antigens to cognate T cells (Lee *et al*, 2010a). In addition, sustained autophagy responses in B and T cells are required to meet the metabolic demands associated with events of differentiation, clonal expansion, and acquisition of the memory phenotype, as described for CD8^+^ memory T cells generated in response to prolonged lymphocytic choriomeningitis virus infection (Hubbard *et al*, 2010; Ma *et al*, 2013c; Xu *et al*, 2014) and influenza (Puleston *et al*, 2014). CMA is also required for T‐cell activation through selective elimination of the negative regulators ITCH and RCAN (Valdor *et al*, 2014).

Notably, viruses have developed the capacity to block or subvert autophagy at multiple stages of their replication cycle (Cong *et al*, 2020). For example, (i) the murine gammaherpesvirus 68/MHV68 and HSV‐1 have been proposed to exploit BECN1 mimicry strategies to bypass autophagy‐mediated disruption (Orvedahl *et al*, 2007; E *et al*, 2009); (ii) the papain‐like protease domain of CoV‐NL63 binds BECN1 and STING1, thus hindering BECN1‐mediated autophagosome formation and inhibiting IFN production (Devaraj *et al*, 2007; Chen *et al*, 2014); while (iii) the Middle East respiratory syndrome (MERS)‐CoV promotes BECN1 degradation (Oudshoorn *et al*, 2017; Gassen *et al*, 2019); (iv) human papilloma virus inhibits autophagy in oropharyngeal squamous cells through E7‐mediated degradation of AMBRA1 (Antonioli *et al*, 2020); and (v) human cytomegalovirus suppresses autophagy flux in epithelial renal cells (Lopez Giuliani *et al*, 2020). Recently, it has been shown that ORF3a of the COVID‐19 virus SARS‐CoV‐2 may suppress autophagy activity. Individual ORF3a expression causes lysosomal damage, while preventing the interaction between the homotypic fusion and protein sorting (HOPS) complex and the autophagosomal soluble N‐ethylmaleimide‐sensitive factor attachment protein receptor (SNARE) protein STX17 (syntaxin 17), eventually undermining the assembly of the STX17‐SNAP29‐VAMP8 SNARE macro‐complex, which regulates the fusion of the autophagosome with the lysosome (Miao *et al*, 2021). In this scenario, it is tempting to speculate that autophagy hijacking by SARS‐CoV‐2 contributes to exacerbate the inflammatory burden associated with viral infection, possibly contributing to the aberrant type I IFN response observed in COVID‐19 patients (Deretic, 2021). Upon picornavirus (e.g., coxsackievirus and rhinovirus) infection, the host lipid‐modifying enzyme PLAAT3/PLA2G16 promotes the delivery of the single‐stranded RNA viral genome to the cytosol before autophagy‐dependent degradation (Staring *et al*, 2017). In addition, mice in which *Atg5* is selectively deleted in pancreatic acinar cells display resistance to coxsackievirus‐induced pancreatitis (Alirezaei *et al*, 2012). Although it is unclear whether picornavirus and herpesviruses hijack the autophagy pathway, components of the ATG machinery have been found in association with membranous platforms utilized by these viruses for replication. Interestingly, these viruses also appear to even subvert non‐canonical autophagy secretion to promote virion egress (Matsuzawa‐Ishimoto *et al*, 2018; Keller *et al*, 2020b). A pro‐viral function of autophagy has been described in circumstances of Junín virus (JUNV) infection (the etiological agent of Argentine hemorrhagic fever), as suggested by the fact that the replication capacity of JUNV was markedly reduced upon Atg5 or Beclin 1 genetic suppression (Roldan *et al*, 2019). Likewise, proficient autophagy responses appear to support the replicative capacity of Dengue virus (Heaton *et al*, 2010; Lee *et al*, 2018b). In addition, hepatitis C virus (HCV) stimulates the induction of autophagy via multipronged mechanisms to promote its replication and egress from infected cells (Shrivastava *et al*, 2012; Hansen *et al*, 2017).

### Inflammatory disorders of the bowel

In view of the multifaceted implications of autophagy in the systemic and local responses to infectious cues, intense research has been dedicated to delineate the role of the autophagy pathway in non‐infectious inflammatory disorders, with particular emphasis on supraphysiological inflammatory responses affecting the gastrointestinal tract (Table 9). In particular, a significant body of literature has established a robust nexus between defective autophagy and inflammatory bowel disease (IBD), such as Crohn disease and ulcerating colitis (Matsuzawa‐Ishimoto *et al*, 2018). The most common mutant variant ATG16L1^T300A^, which renders the protein a target for CASP3‐dependent cleavage, increases the risk of developing Crohn disease (Lassen *et al*, 2014; Murthy *et al*, 2014). Supporting a role for compromised autophagy in preventing the “leaky gut” and dysbiosis associated with IBD pathogenesis, Crohn disease patients harboring the ATG16L1^T300A^ variant and various autophagy gene mutant mice exhibit defective secretion of antimicrobials and production of secretory granules in Paneth cells, a specialized epithelial cell type that protects the intestinal stem cell niche (Cadwell *et al*, 2008; Cadwell *et al*, 2009; Cabrera *et al*, 2015; Bel *et al*, 2017). Hypomorphic expression of ATG16L1 or knock‐in T300A mutation sensitizes mice to infection by commensal virus, while intensifying the inflammatory response to dextran sulfate sodium‐induced intestinal injury (Cadwell *et al*, 2010; Kernbauer *et al*, 2014; Matsuzawa‐Ishimoto *et al*, 2017). Through preserving organelle homeostasis, ATG proteins have a conserved function in mice and humans in promoting the resilience of the intestinal barrier to metabolic and immune‐mediated damage and preventing necrotic cell death of the epithelium (Matsuzawa‐Ishimoto *et al*, 2017; Aden *et al*, 2018; Matsuzawa‐Ishimoto *et al*, 2020; Xie *et al*, 2020). This concept is reinforced by the finding that Paneth cell‐specific deletion of multiple *Atg* genes, especially when deleted together with the ER stress gene *Xbp1*, leads to intestinal inflammation (Adolph *et al*, 2013). In support of the tenet that autophagy represses the inflammatory cascade in IBD, susceptibility genes associated with Crohn disease (i.e., *Nod2*, see also above) stimulate autophagy downstream of bacterial invasion to dampen inflammasome overactivation (Travassos *et al*, 2010; Matsuzawa‐Ishimoto *et al*, 2018). Because IBD‐sensitizing mutations occur at the germline level, it is presumed that a generalized impairment of autophagy, affecting also immune cells that infiltrate the gastrointestinal tract, contributes to the clinical outcomes of IBD, such as T_REG_ cells (Kabat *et al*, 2016) and epithelial cells (Pott *et al*, 2018). In this scenario, it cannot be discounted that non‐canonical tasks of ATG proteins contribute to the aetiopathogenesis of IBD. As an example, commensal *Bacteroides fragilis*‐induced activation of LAP drives a transcriptionally tolerogenic program of differentiation in antigen‐presenting cells, which is required to generate immunosuppressive T_REG_ cells in the context of colitis (Chu *et al*, 2016). Recently, it has been shown that functional IRGM1 (immunity‐related GTPase family M member 1), a Crohn disease risk factor (Parkes *et al*, 2007) which participates in the autophagy‐dependent elimination of intracellular pathogens (Singh *et al*, 2006; Kumar *et al*, 2020), dampens IL1B maturation by interfering in NRLP3 inflammasome assembly. Mechanistically, IRGM promotes the autophagy‐mediated degradation of NLRP3 and PYCARD/ASC, while reducing signs of accrued inflammation in a mouse model of Crohn disease (Mehto *et al*, 2019).

### Other autoimmune disorders

In contrast with the protective role of autophagy in IBD, overexuberant autophagy may exacerbate autoimmunity in rheumatoid arthritis (Xu *et al*, 2013; Matsuzawa‐Ishimoto *et al*, 2018). Mechanistically, this phenomenon appears to be linked to aberrant self‐antigen presentation, maladaptive survival of T helper 17 (T_H_17)‐CD4^+^ T cells and exacerbated response to IL17‐derived inflammatory signals (Ireland & Unanue, 2011; van Loosdregt *et al*, 2016; Kim *et al*, 2017). In large‐scale genome‐wide association studies, a significant correlation has emerged between multiple *ATG* genes and susceptibility to systemic lupus erythematosus, an autoimmune disorder characterized by autoantibody production, aberrant inflammation and multiorgan injury (Qi *et al*, 2019). In human, autophagy is hyperactive and required for autoantibody‐producing B cells (Clarke *et al*, 2015). Abnormal upregulation of CMA has also been described in systemic lupus erythematosus, and a phosphopeptide that significantly ameliorates clinical manifestations of the disease has CMA‐inhibitory properties (Macri *et al*, 2015; Wang *et al*, 2020b). While these results may highlight the hyperactivation of autophagy as a common feature of different autoimmune disorders, additional studies are required to solve this enigma. As an example, conflicting evidence can be inferred from murine models of systemic lupus erythematous. On the one hand, the activation of autophagy in B cells supports the production of autoantibodies in two distinct murine models of systemic lupus erythematous (Weindel *et al*, 2015); on the other hand, concomitant deletion of *Atg5* in DCs and B cells precipitates the inflammatory phenotype, lending further support to the hypothesis that autophagy can mediate cell type‐exclusive function in distinct autoimmune pathologies (Weindel *et al*, 2017). Adding a further layer of complexity, non‐canonical autophagy is implicated in similar autoimmune processes, as testified to by the fact that LAP is necessary for the type I IFN response during internalization of DNA–antibody complexes by plasmacytoid DCs (Henault *et al*, 2012; Hayashi *et al*, 2018; Leylek & Idoyaga, 2019), while also mediating the turnover of dying cells by myeloid cells to prevent the generation of such antibody complexes (Martinez *et al*, 2016). A non‐canonical role for ATG proteins has been also described in a model of experimental autoimmune encephalomyelitis (a CD4^+^ T‐cell‐mediated mouse model of multiple sclerosis) where targeted knockout of *Atg5* or *Atg7* in DCs abrogates myelin presentation to myelin‐specific CD4^+^ T cells, hence preventing the accumulation of autoimmune T cells within the CNS and the consequent CNS damage (Bhattacharya *et al*, 2014; Keller *et al*, 2017; Berglund *et al*, 2020).

## Ocular diseases

Visual impairment is among the leading disorders in developed countries, being that aging is the major cause for its clinical manifestation. In support of the involvement of autophagy in the age‐dependent decay of eye function, reduced mRNA expression of essential autophagy regulators, accompanied by increased markers of defective autophagy flux, has been reported in the retina of old mice (Rodriguez‐Muela *et al*, 2015). In view of its inherent function of cytoprotection elicited in neuronal precursors and in the multitude of differentiated cell types that form the eyeball, bulk and selective types of autophagy operate at the frontline to preserve visual integrity (Boya *et al*, 2016) (Table 10).

**Table 10:** Ocular diseases associated with genetic intervention of autophagy in mice.

Setting	Genetic intervention	Effects on disease phenotype	Ref.
ADOA	RGC‐specific deletion of *Atg5*	Ameliorated visual defects driven by *Opa1* ablation by normalizing the autophagic flux	Zaninello *et al* (2020)
ARMD	RPE‐specific deletion of *Rubcn*	Prevention of the inflammatory response to chronic blue light exposure by limiting autophagy impairment	Ando *et al* (2021)
ARMD	Whole‐body deletion of *Lamp2*	Accelerated age‐associated formation of basal laminar deposits in the retina	Notomi *et al* (2019)
Cataract	LEC‐specific deletion of *Atg5*	Development of lens clouding by 21 months of age	Morishita *et al* (2013)
Cataract	LEC‐specific deletion of *Vps34*	Development of congenital cataract and microphthalmia, through an autophagy‐independent mechanism	Morishita *et al* (2013)
Glaucoma	Overexpression of mutant *Optn^E50K^*	Increased RGC death and reduced retinal thickness, linked to profound gliosis in the retina	Chi *et al* (2010), Minegishi *et al* (2013)
Retinal development	Whole‐body deletion of *Atg5* or *Bnip3l*	Inhibited RGC differentiation after mitophagy inhibition	Esteban‐Martinez *et al* (2017)
Retinal degeneration	Whole‐body deletion of *Atg4b*	Reduced numbers of surviving RGCs after optic nerve transection	Rodriguez‐Muela *et al* (2012)
Retinal degeneration	Conditional RGC‐specific deletion of *Atg5*	Reduced numbers of surviving RGCs after optic nerve transection	Rodriguez‐Muela *et al* (2012)
Retinal degeneration	Whole‐body allelic loss of *Becn1*	Increased susceptibility to light‐induced retinal damage	Chen *et al* (2013)
Retinal degeneration	Whole‐body deletion of *Prkn*	Exacerbated light‐induced retinopathy linked to accumulation of damaged mitochondria	Chen *et al* (2013)
Retinal degeneration	Conditional rod photoreceptor‐specific deletion of *Atg7*	Increased susceptibility to light‐induced retinal damage linked to increased photoreceptor cell death	Chen *et al* (2013)
Retinal degeneration	Conditional RPE‐specific deletion of *Rb1cc1*	Increased age‐dependent degeneration of the RPE, and secondary degeneration of the overlying photoreceptors	Yao *et al* (2015)
Retinal degeneration	Conditional RPE‐specific deletion of *Atg5*	Decreased photoreceptor responses to light stimuli linked to disrupted lysosomal processing	Kim *et al* (2013a)
Retinal degeneration	Conditional rod photoreceptor‐specific deletion of *Atg5*	Progressive degeneration of rod photoreceptors by 8 weeks of age, independently of light exposure	Zhou *et al* (2015a)
Retinal degeneration	Cone cell‐specific deletion of *Atg5*	Increased susceptibility to light‐induced retinal damage linked to accumulation of damaged mitochondria	Zhou *et al* (2015b)

AOA, autosomal‐dominant optic atrophy; ARMD; age‐related macular degeneration; LEC, lens epithelial cell; RGC, retinal ganglion cell; RPE, retinal pigment epithelium.

Intact autophagy supports the regression of the hyaloid artery that accompanies eye maturation (Kim *et al*, 2010). Because the constitutive knockout of key autophagy genes results in embryonic or perinatal lethality, the retinal phenotype of these animal models has not been characterized in detail, although the specific deletion of *Atg5* in neuronal precursors results in a very dramatic phenotype of photoreceptor death and night vision loss already at 7 weeks of age (Rodriguez‐Muela *et al*, 2013). *Ambra1*‐deficient zebrafish models exhibit ocular dysfunction during embryonic development (Benato *et al*, 2013). In addition, *Atg5*‐deficient mouse retinas display a reduced number of retinal ganglion cells during development and alterations in retina metabolism (Esteban‐Martinez *et al*, 2017). Whereas models of partial autophagy deficiency (i.e., *atg4b^−^* ^/^^−^ mice) do not display visual impairment under baseline conditions, they are characterized by accrued sensitivity to axonal damage (Rodriguez‐Muela *et al*, 2012). Likewise, *Becn1^+^* ^/^^−^ animals exhibit exacerbated retinal damage upon prolonged exposure to bright light (Chen *et al*, 2013), and old *ambra1^+^* ^/^ *^gt^* exhibit accrued sensitivity to optic nerve crush (Bell *et al*, 2020). Conditional *rb1cc1* deletion in retinal pigment epithelium (RPE) leads to severe visual impairment, linked to reduced RPE proteostatic functions (Yao *et al*, 2015). In line with these observations, conditional deletion of *Atg5* in the RPE does not affect eye function at birth, yet manifests as declining photoreceptor functions at old age, linked to impaired lysosomal degradation of photoreceptor outer segments. In this context, autophagy‐independent functions of the ATG machinery are instrumental in regulating the vision cycle, as shown by the fact that the ATG5‐ and BECN1‐dependent (but ULK1 independent) conjugation of LC3 to phagosomal membranes is required for phagocytosis and degradation of photoreceptor outer segments (POS) in RPE (Kim *et al*, 2013a). The conditional knockout of *Atg7* in rod cells causes severe degeneration of the superior retina only upon exposure to bright light (Chen *et al*, 2013). However, conditional *Atg5* deficiency in rod photoreceptors results in age‐dependent rod degeneration, even in animals raised in darkness, implying a gene‐specific degree of severity (Zhou *et al*, 2015a). Along similar lines, deletion of *Atg5* in cone cells progressively affects cone number and function across mouse lifespan, making animals more sensitive to light‐induced degeneration (Zhou *et al*, 2015b). In addition, deletion of *Atg5* in cone cells progressively affects cone number and function across mouse lifespan, making animals more sensitive to light‐induced degeneration (Zhou *et al*, 2015b). In animal models of retinitis pigmentosa, lysosomal membrane rupture and overexuberant MTOR pathway activation causally contribute to photoreceptor decay (Rodriguez‐Muela *et al*, 2015). Conversely, the activation of autophagy promoted by HDAC3 inhibition (Samardzija *et al*, 2020) and trehalose treatment limits photoreceptor degeneration, thus preserving visual acuity (Lotfi *et al*, 2018).

Alterations in the ATG machinery contribute to the pathogenesis of ocular diseases caused by dysfunction in different cellular components forming the eyeball. Mice harboring LEC‐specific *atg5* deletion develop lens clouding by 21 months of age (Morishita *et al*, 2013). A similar effect occurs upon *pik3c3*/*vps34* deletion in LECs, which also leads to age‐dependent cataracts (Morishita *et al*, 2013). Of note, this effect does not rely on the autophagy‐dependent degradation of organelles, which is postulated to be essential to generate an organelle‐free transparent zone. Recent findings rather suggest that organelle degradation in LECs depends upon functional PLAAT/HRASLS (phospholipase A and acyltransferase) phospholipases, which induce organelles rupture followed by their complete degradation (Morishita *et al*, 2021).

Congenital forms of cataracts have been associated with mutations in the LC3 and RAB7 binding protein FYCO1 (FYVE and coiled‐coil domain autophagy adaptor 1), which also takes part in autophagosome trafficking and fusion with lysosomes (Chen *et al*, 2011). Likewise, a knock‐in mouse model bearing the R120G mutation in CRYAB/αB‐crystallin, which leads to human congenital cataracts, displays an impaired autophagy flux (Wignes *et al*, 2013).

Experimental findings (mostly *in vitro* studies) showed that autophagy elicits protective functions in age‐related macular degeneration (ARMD), which manifests in humans in a dry or wet form. ARMD pathogenesis is linked to events of altered proteostasis and aberrant oxidative stress, associated with the prominent accumulation of lysosomal lipofuscin granules and extracellular proteinaceous deposits (known as “drusen”) in RPE of the basal layer, which cause progressive degeneration of post‐mitotic RPE. In two different mouse models of ARDM (*Sod2* knockdown and the *apoe*/*APOE4*‐HFC model), autophagy is upregulated at the early stage of the disease, yet declines at advanced stages of the pathology (Mitter *et al*, 2014; Song *et al*, 2017). In support of this result, the induction of autophagy is required to dispose of the lipofuscin component A2E in RPE, which progressively accumulates with age (Zhang *et al*, 2015). A2E in RPE inhibits autophagy partly through upregulation of RUBCN (Ando *et al*, 2021). In this scenario, treatment with rapamycin improves A2E degradation (Zhang *et al*, 2015). Further corroborating the idea that impaired lysosomal function is pathognomonic to ARMD, animal models deficient in CRYBA1/bA3/A1‐crystallin display impaired lysosomal acidification in RPE, culminating in RPE degeneration and signs of ARMD (Valapala *et al*, 2014). Moreover, the pathogenesis of human dry ARMD is characterized by the loss of LAMP2 expression by RPE cells, and the knockout of *Lamp2* suffices to cause an ARMD‐like disease in mice (Notomi *et al*, 2019).

Glaucoma, a progressive optic neuropathy that leads to retinal ganglion cell (RGC) degeneration, is among the leading causes of blindness. Primary open angle glaucoma (POAG) is commonly associated with elevated intraocular pressure (IOP) and aging. The occlusion of the trabecular meshwork that regulates aqueous humor outflow from the anterior chamber of the eye is a major cause for POAG; yet, genetic factors, vascular alterations, and autoimmune reactions have also ascribed a causative role. A second form of glaucoma, called normal tension glaucoma (NTG), is not associated with elevated IOP. The clinical outcome of both glaucoma subtypes is visual loss caused by RGC degeneration. Autophagy has been implicated in both the etiological phase of elevated IOP generation in POAG and the etiological phase of RGC loss in both POAG and NTG. Commonly, outflow from the eye anterior chamber is inhibited by mutations in MYOC (myocilin) that can be recapitulated in the mouse. Interestingly, stimulation of autophagy can clear mutant MYOC accumulation and correct IOP elevation (Kasetti *et al*, 2021). Decreased autophagy flux has been reported in RGC upon chronic IOP elevation (Hirt *et al*, 2018). In contrast, others have reported that autophagy is chronically activated in RGCs of aged mice with elevated IOP (Nettesheim *et al*, 2020). In line with these controversies, autophagy appears to protect or promote RGC death depending on the experimental model and the time point analyzed (Koch & Lingor, 2016). For example, the expression of a GFP‐LC3 transgene exacerbates optic nerve degeneration in a mouse model of spontaneous IOP, pointing to a detrimental role for excess autophagy (Hirt *et al*, 2018). A similar situation has been reported in the case of autosomal‐dominant optic atrophy (ADOA), a genetic form of RGC degeneration caused by dominant‐negative mutations in, or haploinsufficiency of, the mitochondrial dynamic‐regulating gene *OPA1*. *In vitro* and *in vivo* experiments have demonstrated that the pathological phenotype of ADOA depends on excessive autophagy, and genetic normalization of the autophagy flux fully corrects the visual loss observed in the ADOA mouse model (Zaninello *et al*, 2020). A role for reduced mitophagy has been identified in NTG, associated with mutations in the autophagy receptor gene *Optn* (the most common being E50K and M98K). Transgenic mice overexpressing the OPTN^E50K^ mutation, which instigates the formation of insoluble OPTN aggregates and results in autophagy blockade, display RGC loss and reduced retinal thickness (Chi *et al*, 2010; Minegishi *et al*, 2013). In these settings, pharmacological stimulation of autophagy by rapamycin mitigates OPTN^E50K^‐induced RGC death (Chalasani *et al*, 2014).

Retinal ganglion cell death can be mimicked in mice by optic nerve axotomy (an acute model of glaucoma) and causes retrograde RGC degeneration in a BCL2‐inhibitable manner (Cenni *et al*, 1996; Porciatti *et al*, 1996). Not surprisingly, adenovirus‐mediated depletion of *Atg5* in RGCs sensitizes RGCs to optic nerve axotomy‐induced death (Rodriguez‐Muela *et al*, 2012). Therefore, upon optic nerve axotomy autophagy is activated (via canonical and non‐canonical routes) to promote RGC survival (Rodriguez‐Muela *et al*, 2012). Supporting this finding, pharmacological activation of autophagy by rapamycin shows protective effects in multiple experimental models of glaucoma. (Rodriguez‐Muela *et al*, 2012; Kitaoka *et al*, 2013; Su *et al*, 2014; Russo *et al*, 2018; Wen *et al*, 2019; Lee *et al*, 2021).

As ocular disorders are in the vast majority of the cases multifactorial, or associated with concurrent pathologies, it is tempting to speculate that lifestyle factors or chronic diseases that undermine autophagy (i.e., diabetes) contribute to the pathological phenotype in the eye also via autophagy downregulation, as in the case of diabetic retinopathy (Boya *et al*, 2016).

## Reproductive system dysfunctions

Endometriosis is a benign gynecological disease, associated with dysmenorrhea, pelvic pain, and infertility in women. Accumulating evidence reveals a pivotal role for autophagy in the pathogenesis of endometriosis (Yang *et al*, 2017c). While in normal endometrium autophagy is induced as a pro‐apoptotic mechanism in glandular epithelial and stromal cells during menstruation (Choi *et al*, 2012), increased autophagy mediates hyperplasia of murine endometriotic tissue and stromal cells (Ruiz *et al*, 2016), thus limiting apoptosis and promoting abnormal immune responses (Yu *et al*, 2016). Consistently, genetic or pharmacological inhibition of autophagy prevents the formation of endometriotic lesions (Liu *et al*, 2017) (Table 11).

**Table 11:** Reproductive system dysfunctions.

Setting	Genetic intervention	Effects on disease phenotype	Ref.
Male infertility	Germ cell‐specific deletion of *Atg7*	Reduced motility of spermatozoa with malformed head, linked to impaired cytoskeleton organization	Shang *et al* (2016)
Male infertility	Sertoli cell‐specific deletion of *Atg7* or *Atg5*	Disorganized seminiferous tubules and spermatozoa with malformed heads, linked to impaired cytoskeleton organization	Liu *et al* (2016)

Dysfunctional autophagy has also been linked to ovarian insufficiency due to inflammatory aging and miscarriage, as well as to male infertility. For example, inhibition of the NLRP3 inflammasome leads to increased levels of autophagy markers in the ovary of 12‐month‐old female mice and is linked to improved reproductive pregnancy rate (Navarro‐Pando *et al*, 2021), whereas pharmacological induction of autophagy (by rapamycin) promotes endometrium autophagy (and NK cell infiltration), thus decreasing the risk of spontaneous abortion in mice (Lu *et al*, 2020). In addition, functional autophagy sustains correct spermiogenesis. For example, *atg7* ^−/−^ mice show defects in cytoskeleton organization limiting the differentiation of spermatids (Shang *et al*, 2016) and autophagy disruption in Sertoli cell results in the formation of disorganized tubules and production of low motility malformed spermatozoa (Liu *et al*, 2016; Shang *et al*, 2016).

## Concluding remarks

Taken together, these observations point to autophagy as a primordial determinant of human health, thus delineating autophagy‐modulating interventions as promising approaches to prevent or mitigate phenotypic anomalies of the most common human illnesses. While the introduction of conditional knockout murine models of disease has enabled researchers to shed new light on the cell type inherent functions of autophagy, these models still present important limitations, in that they fall short in capturing the multidimensional relationships among cell types, which often rely upon non‐cell autonomous effects of the autophagy route, at the tissue and systemic level. Moreover, the majority of the genetic models employed in autophagy research are not inducible, and hence establish an autophagy defect either at fecundation or upon activation of the tissue‐restricted promoter employed to control Cre expression. Even in the latter scenario, this generally occurs during development, and hence fails to recapitulate an acute autophagic defect in the adult. Autophagy also intersects with other pathways (e.g., LAP, LANDO, RCD) at multiple signaling nodes. As most of the results discussed herein were obtained upon the deletion or downregulation of single components of the autophagic apparatus, the observed phenotypes may actually originate from non‐autophagic pathways that share core regulators with autophagy. Thus, future studies examining the role of autophagy in disease should rely on genetic deletions of more than one autophagy gene, preferably encompassing early and late functions, and on recently derived genetic models that can differentiate canonical from non‐canonical autophagy phenotypes. Finally, evidence from human clinical studies, possibly inferred at pre‐pathological stages of the diseases, would ignite the field with important insights about autophagy dynamics in relevant human pathologies.

**Figure 2:**
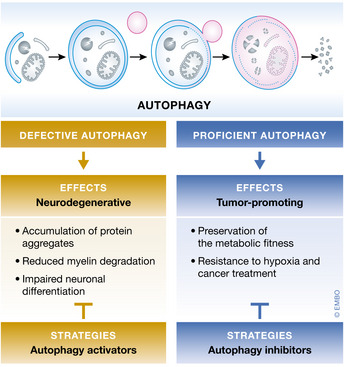
Basic principles of autophagy modulation as a therapeutic strategy for human disease In multiple settings including various neurodegenerative conditions, autophagy defects contribute to disease onset and progression, suggesting that autophagy activators may mediate beneficial effects. Conversely, proficient autophagic responses support tumor progression and resistance to therapy, pointing to autophagy inhibition as an appropriate therapeutic approach. In both scenarios, the effect of autophagy modulation on non‐diseased cells must be carefully considered to enable safety and superior therapeutic efficacy.

Despite these caveats, a few general concepts emerge from the abundant preclinical literature discussed herein. First, autophagy defects are particularly detrimental for post‐mitotic cells (e.g., neurons, cardiomyocytes, memory T cells), largely linked to their accrued demands for long‐term proteostasis. Second, autophagy defects in healthy cells are often connected to disease as a consequence of lost cellular homeostasis rather than failed adaptation to dwindling nutrients. Instead, cancer cells generally harness autophagy as a measure to withstand intracellular stress linked to the malignant status and challenging microenvironmental conditions. Third, autophagic proficiency declines with age, hence contributing to multiple pathologies of the elderly. Finally, a number of commonly accepted lifespan‐ and healthspan‐extending habits (e.g., exercise, caloric restriction) share the ability of activating autophagy. Thus, although much remains to be done, the modulation of autophagy for therapeutic purposes remains a promising strategy for the management of multiple human disorders (Fig 2). The future will tell which specific conditions will be the first to benefit from clinically usable pharmacological autophagy modulators.

## Author contributions

DJK, LG and FP conceived and wrote the manuscript, centralized and integrated comments from co‐authors, and revised the review upon editorial feedback. GP designed the figure, performed bibliographic searches, and helped with table preparation. All authors corrected the article and provided valuable input to obtain a unified view. With the exception of DJK, GP, LG and FP, authors are listed alphabetically, which does not reflect their relative contribution to the preparation of this article.

## Conflict of interest

A.B. is cofounder of CASMA Therapeutics Inc., Advisory Board member of Next Generation Diagnostics and of Avilar Therapeutics. K.C. has received research support from Pfizer, Takeda, Pacific Biosciences, and AbbVie; consulted for or received an honorarium from PureTech Health, Genentech, and AbbVie; and holds U.S. patent 10,722,600 and provisional patents 62/935,035 and 63/157,225. A.M.K.C. is a cofounder, stock holder and serves on the Scientific Advisory Board for Proterris, which develops therapeutic uses for carbon monoxide. A.M.K.C. also has a use patent on CO. G.K is a cofounder and advisor of EverImmune, Samsara Therapeutics, and Therafast Bio as well as advisor for The Longevity Labs (TLL). F.M. is a founder, is advisor, and has equity interests in The Longevity Labs (TLL) and Samara Therapeutics. D.C.R is a consultant for Aladdin Healthcare Technologies SE, Drishti Discoveries, and Nido Biosciences. L.G. has received research funding from Lytix Biopharma and Phosplatin, as well as consulting/advisory honoraria from Boehringer Ingelheim, AstraZeneca, OmniSEQ, Onxeo, The Longevity Labs, Inzen, and the Luke Heller TECPR2 Foundation. RKA is cofounder of Pinpoint Therapeutics and advisor for Deciphera, Sprint Biosciences, Merck, and Immunacell. He gets research funding for clinical trials from Novartis, Bristol Myers Squibb, Pfizer, and Deciphera. J.Y. is a consultant for Denali Therapeutics, Sanofi, and Nido. All other authors have no conflicts of interest to disclose.
